# Synthesized Depside Molecules Suppress the Progression of Colorectal Cancer by Binding VDAC1/PHB/MMP9 Being at the Crossroads of Stemness, Motility, Apoptosis, and Metabolism

**DOI:** 10.1002/mco2.70446

**Published:** 2025-10-31

**Authors:** Mücahit Varlı, Young Hyun Yu, Jieun Yu, Suresh R. Bhosle, Sang Kyum Kim, Yoon Gyoon Kim, Hyung‐Ho Ha, Hangun Kim

**Affiliations:** ^1^ College of Pharmacy Sunchon National University Sunchon Jeonnam Republic of Korea; ^2^ College of Pharmacy Chungnam National University Daejeon Republic of Korea; ^3^ College of Pharmacy Dankook University Cheonan‐si Republic of Korea

**Keywords:** matrix metalloproteinase‐9, probe‐based direct binding identification, prohibitin 1, synthesized depside molecules, voltage‐dependent anion‐selective channel 1

## Abstract

Lichen secondary metabolites have shown potential in cancer therapy, but strategies to enhance cancer‐specific selectivity are needed. Here, we synthesized depside compounds structurally related to tumidulin (TU) and diffractaic acid (DA) and screened them in vitro, identifying SB4 and SB5 as potent hits. Affinity‐based proteomics revealed direct binding to voltage‐dependent anion channel 1 (VDAC1), prohibitin (PHB), and matrix metalloproteinase‐9 (MMP9), which regulate cancer stemness, motility, metabolism, and apoptosis. SB4 and SB5 exhibited strong cytotoxicity, suppressed cancer stem cell characteristics, inhibited cell motility, impaired mitochondrial respiration, induced reactive oxygen species, and promoted apoptosis. Notably, they reversed cetuximab‐induced cancer stemness in colorectal adenocarcinoma‐enriched stem cells. In vivo, SB4 and SB5 displayed higher tumor, liver, and intestinal bioavailability than TU and DA following intraperitoneal administration. Pharmacokinetic analyses indicated SB4 had a comparable absorption profile to SB5 with distinct systemic exposures differences. In a CT26/near‐infrared fluorescent protein tumor model, SB4 markedly inhibited tumor growth and modulated key markers of stemness, motility, metabolism, and apoptosis in tumor tissues. Collectively, these findings demonstrate that SB4 and SB5 are promising candidates for colorectal cancer therapy by targeting VDAC1/PHB/MMP9.

## Graphical abstract

This study suggests that newly synthesized depside compounds suppress the progression of colorectal cancer by directly targeting VDAC1/PHB/MMP9. Especially, SB4 demonstrated significant bioavailability and successfully suppressed the tumor growth.

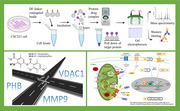

## 1 Introduction

Unfortunately, every year 19 million people are diagnosed with cancer and have to fight against it, while 10 million people die [1]. According to estimates, colorectal cancer (CRC) will account for 162,820 new cases and 54,540 deaths in the United States in 2022, making it the third most frequent disease globally [2]. DNA anomalies in somatic tissues that do not transfer to progeny are referred to as somatic mutations. Typically, CRC starts with normal colonic mucosa accumulating somatic mutations (*APC*, *KRAS*, *P53*, etc.). Then, those mutant cells cause aberrant growth of glandular mucosa and epithelium, which eventually spreads to other body organs like the liver and lung [3]. Advanced CRC often remains refractory to treatment despite advances in modern treatment approaches such as surgery, chemotherapy, and targeted therapies. This calls for further research into new therapeutic targets and the critical need for personalized strategies that take into account the molecular complexity of the disease [2, 4].

Tumorigenesis and metastasis are caused by a multitude of variables such as cancer metabolism and cancer stemness. Cancer cells require bioenergetics to sustain cell proliferation and survival through the metabolism of glucose, fatty acid, and amino acids [5]. Modifications in metabolic gene‐induced regulation, glycolysis/tricarboxylic acid (TCA) cycle intermediates required for macromolecule synthesis, and dysregulated intake of glucose and amino acids are caused several hallmarker of cancer. Additionally, cancer stem cells (CSCs) have been associated with increased mitochondrial oxidative metabolism, which has been attributed to stem‐like properties, DNA damage, and metastatic ability [6]. The capacity of CSCs to self‐renew and to stimulate the formation of heterogeneous cancer cells are well known. There is mounting evidence that CSCs have a role in chemoresistance, which reduces the effectiveness of anticancer medications [7]. Many research reports have reported that chemotherapeutics promote CSCs [8, 9], so it is important to develop new treatment options that may also be effective against CSCs in cancer treatments.

Lichens are distinct from higher plants in that they contain a variety of chemicals, including terpene, aromatic, cyclic aliphatic, and aliphatic ones [10]. Promising natural compounds called depsides and their derivatives are present in lichens. They also exhibit intriguing biological activity such as antibacterial, antiviral, antibiotic, antioxidant, anti‐inflammatory, antipyretic, analgesic, and anticancer [11, 12, 13]. Anziaic acid, norstictic acid, gyrophoric acid, evernic acid, atranorin, tumidulin (TU), and diffractaic acid (DA) are examples of secondary metabolites of depside lichen [14, 15, 16]. In this study, we synthesized analogs based on the structures of TU and DA. In our previous study, we showed that TU suppressed CRC stemness markers and sonic hedgehog (SHH) signaling [17] and DA analogs suppresses the CRC stemness potential [18]. While the general biological activity of depside compounds has been previously reported in research, their molecular mechanisms to suppress anticancer activity, particularly CRC, remain poorly elucidated. Also, depsides have not fully been investigated with respect to the modulation of oncogenic pathways related to cancer stemness, metabolic reprogramming, and apoptosis. This study aimed to investigate how structural analogs of TU and DA interact with key molecular targets (voltage‐dependent anion channel 1 [VDAC1], PHB, and matrix metalloproteinase [MMP]9), while also considering their bioavailability of their potential to modulate CRC development. We show that bioactivity‐guided depside synthesis will produce more selective and more potent compounds against CRC. By employing affinity‐based proteomics and mass spectrometry‐directed target identification, we revealed a novel functional axis through which synthesized depside molecules modulate mitochondrial function, extracellular matrix (ECM) dynamics, and survival signaling. Not only does this integrative approach provide mechanistic insight into the manner in which depside structures modulate colorectal tumor biology, but it also provides a platform for rational design of depside‐inspired drugs. To the best of our knowledge, this is the first study to delineate direct protein targets of depside analogues with unbiased chemical biology tools within the context of CRC, which attests to the novelty significance of our findings.

VDAC1/PHB/MMP9, the target of the synthetic depside compounds used in this study, play critical roles in the regulation of mitochondrial functions, survival, metabolism, stem cell potential, and invasion capacity in cancer cells, and changes in the expression levels of these targets can have decisive effects on tumor progression and treatment responses [19, 20, 21, 22, 23, 24, 25]. Therefore, pharmacological strategies targeting the function of cotargeting VDAC1/PHB/MMP9 are being considered as a potential avenue for the treatment of CRC. In this context, our study aims to elucidate the molecular effects of depside‐derived compounds by simultaneous targeting of VDAC1, PHB, and MMP9 as a promising strategy for the treatment of CRC.

## 2 Results

### 2.1 Synthesized Compounds Show Higher Suppression Capacity Compared with Parent Natural Products

**FIGURE 1:**
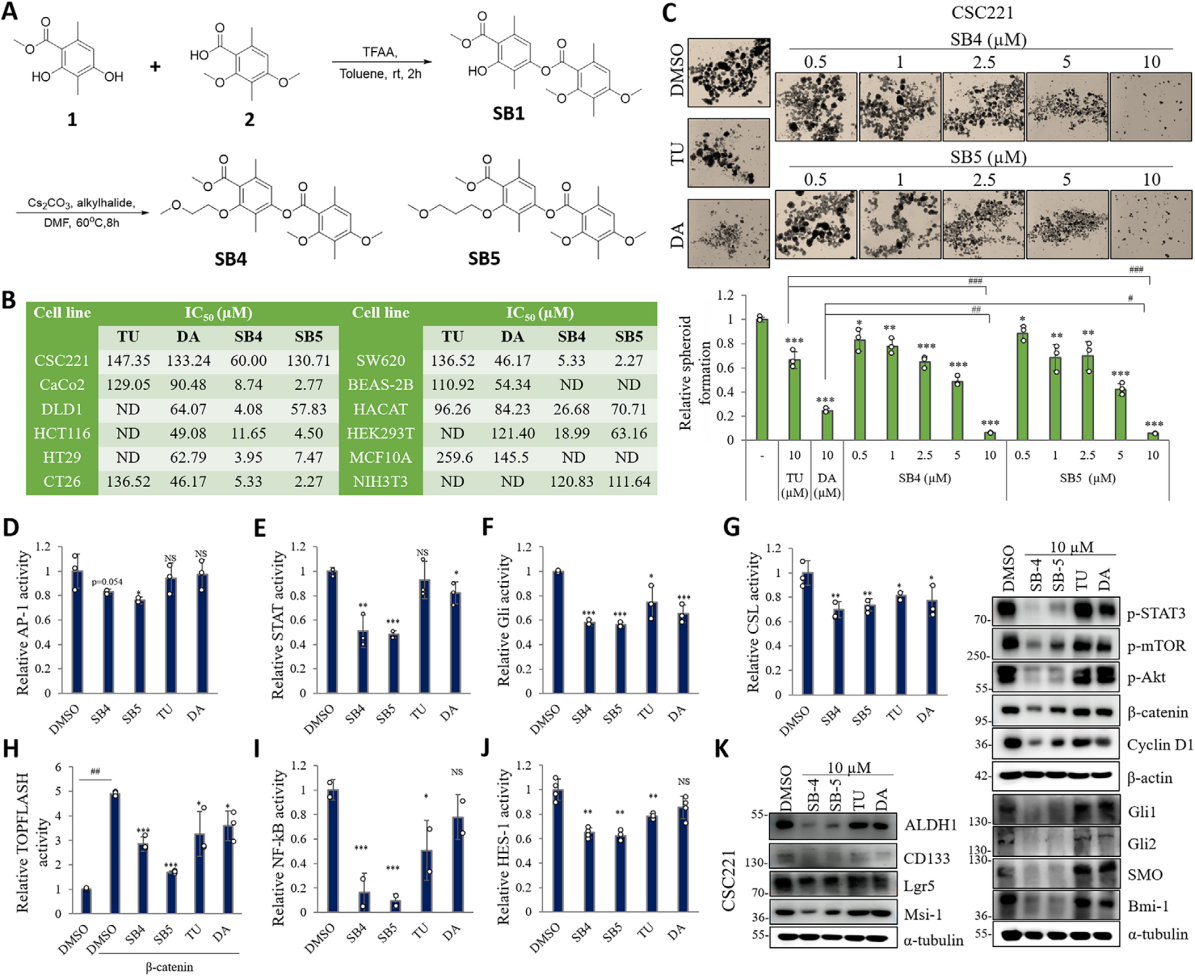
Newly synthesized depside molecules exhibit more inhibition activity on cell viability, colorectal cancer stemness markers, and cellular signaling compared the tumidulin and diffractaic acid. (A) Synthesis of SB4 and SB5 compounds. (B) IC_50_ values of tumidulin (TU), diffractaic acid (DA), SB4 and SB5 in CSC221, CaCo2, DLD1, HCT116, HT29, CT26, SW620, BEAS‐2B, HaCaT, HEK293T, MCF10A, NIH3T3. Cells were treated with compounds for 48 h, and cell viability was measured by MTT assay. (ND: not determined). (C) Cells were exposed to the compound at the indicated concentrations (up to 10 µM) for 14 days. The histogram represents spheroid formation, calculated as rate relative to vehicle‐treated control, and represented as bar graphs. Data are presented as the mean ± standard deviation, *n* = 3. The asterisk indicates a significant difference compared with the DMSO control, *p < 0.05; **p < 0.01; ***p < 0.001; NS, not significant. The hashtag indicates a significant difference between treatment groups, #*p* < 0.05; ##*p* < 0.01; ###*p* < 0.001; NS, not significant. Effect of compounds on (D) AP‐1, (E) STAT, (F) Gli, (G) CSL, (H) β‐catenin‐mediated TOP‐Flash, (I) NF‐κB, and (J) Hes‐1 luciferase activity. HEK293T cells were treated with indicated luciferase plasmid for 24 h, after that cells were treated with compounds for 24 h. Data are presented as the mean ± standard deviation, *n* = 2–4. The asterisk indicates a significant difference compared with the DMSO control, *p < 0.05; **p < 0.01; ***p < 0.001; NS, not significant. (K) CSC221 cells were treated with compounds for 48 h. Proteins were analyzed by immunoblotting to detect indicated markers. Western blots were repeated three times.

In this study, we synthesized analogs derived from TU and DA, which have a two‐depside molecule structure. We obtained the synthesized molecules as shown in Figure 1A. Cytotoxicity of the TU, DA, SB4, and SB5 compounds were tested against several human CRC cell lines: CSC221, CaCo2, DLD1, HCT116, HT29, and SW620, a mouse colon cancer cell line CT26, and noncancerous cell lines BEAS‐2B, HaCaT, HEK293T, NIH3T3, and MCF10A. Compared with TU and DA, SB4 and SB5 exhibited superior inhibitory activity and pronounced cytotoxic effects on human CRC cell lines. However, SB4 and SB5 showed low cytotoxic effects on the CSC221 (human colorectal adenocarcinoma‐enriched CSCs) cell line compared with other CRC cell lines. However, according to cytotoxicity screenings in noncancerous cell lines, SB4 and SB5 compounds have selective cytotoxic effects on cancer cell lines (Figures 1B, S1, and S2). Moreover, we tested the effects of SB4 and SB5 on spheroid formation by comparing them with TU and DA on the CSC221 cell line (Figure 1C). Phenotypically, SB4 and SB5 suppress spheroid formation more strongly than TU and DA. According to the results of AP‐1, STAT, Gli, CSL, β‐catenin‐mediated TOP‐Flash, NF‐κB, and Hes‐1 luciferase reporter assay, SB4 and SB5 exhibit a better inhibition ability, confirming the phenotypic results (Figure 1D–J). To further characterize, we tested the protein levels of ALDH1, CD133, Lgr5, Msi1, p‐STAT3, p‐mTOR, p‐Akt, β‐catenin, CyclinD1, Gli1, Gli2, SMO, and Bmi‐1 (Figure 1K). Our results showed that SB4 and SB5 had stronger effects on stem cell markers and stem cell regulating signaling.

### 2.2 Identification of Cellular Targets of SB4 and SB5 by Chemical Probe

**FIGURE 2:**
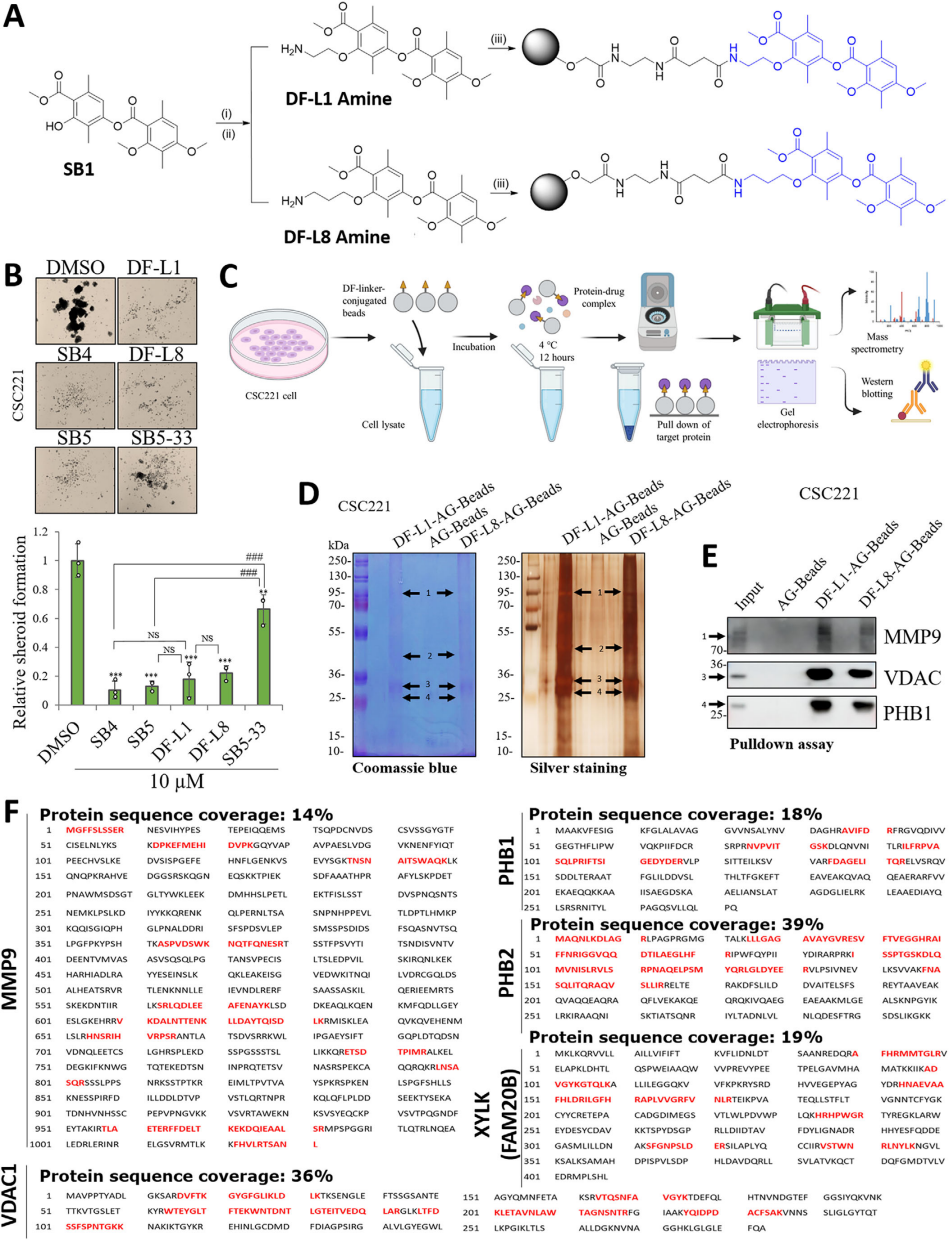
Identification of direct target of SB4 and SB5. (A) Synthetic scheme of DF‐L1 and DF‐L8 immobilized with affigel‐10; (i) tert‐butyl (2‐bromoethyl)carbamate (DF‐L1 Amine), tert‐butyl (3‐bromopropyl)carbamate(DF‐L8 Amine), K_2_CO_3_, DMF, 50°C, 10 h. (ii) TFA, DCM, rt, 12 h. (iii) Affigel‐10, DMSO, rt, 4 h. The compounds were constructed by covalently attaching SBs linker‐amine. (B) Spheroid formation assay was performed in CSC221 cells, comparing DF‐L1/8, SB5‐33, and SB4/5 itself. Data are presented as the mean ± standard deviation, *n* = 3. *p < 0.05; **p < 0.01; ***p < 0.001; NS, not significant versus the DMSO‐treated group. ^#^ *p* < 0.05; ^##^ *p* < 0.01; ^###^ *p* < 0.001; NS, not significant versus the treatments groups. (C) Schematic representation of experimental processes for protein identification (created with BioRender.com (https://biorender.com)). CSC221 cells were incubated with DF‐linker conjugated beads (4°C, 12 h). Then, the beads were washed six times and the bound proteins pulled down were resolved on SDS‐PAGE. (D) After the SDS‐PAGE and gels staining with Coomassie blue (CBBG‐250) or silver (left panel). Marked regions on the gels indicate bound proteins. Only beads containing Affi‐Gel were used for control purposes. (E) Detection of target proteins through analysis of pull‐down with the chemical probe confirmed by immunoblot analysis using PHB1, VDAC, and MMP9 antibodies. (F) Results of the protein sequence mapping are shown, along with sample peptides from the target protein (shown in red).

**FIGURE 3:**
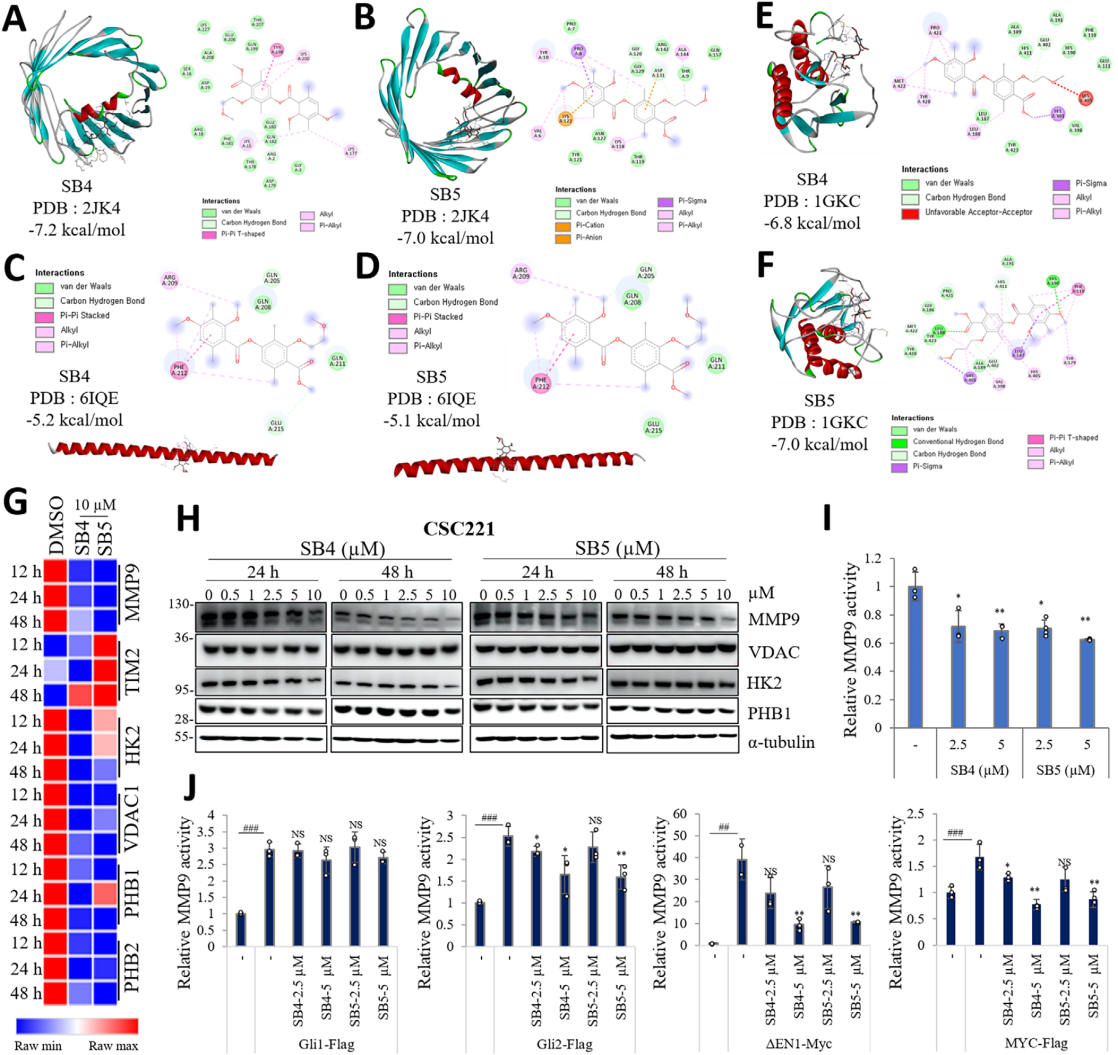
Newly synthesized depside molecules bind to the VDAC1/PHB/MMP9. (A) SB4, (B) SB5 binding the VDAC1 (PDB: 2JK4), (C) SB4, (D) SB5 binding the PHB (PDB: 6IQE). (E) SB4, (F) SB5 binding the MMP9 (PDB: 1GKC). The predicted binding poses were given. Results showed significant interactions (e.g., van der Waals interaction, hydrophobic interactions, hydrogen bonding) in by BIOVIA Discovery Studio visualizer. (G) CSC221 cells were treated for 12, 24, and 48 h with SB4 and SB5 at 10 µM concentration. mRNA expression of MMP9, TIM2, HK2, VDAC1, PHB1, and PHB2 were measured by qRT‐PCR. Heatmap displaying gene expressions. (H) Immunoblots are shown. Relative expression of each target protein after cells were exposed SB4 and SB5 for 24 and 48 h. Western blots were repeated three times. (I) MMP9 promoter assay in HEK293T cells. Cells were transfected with MMP9 promoter plasmid for 24 h, after that treated with 2.5 and 5 µM of SB4 and SB5 for 24 h. Data are presented as the mean ± standard deviation, *n* = 3. The asterisk indicates a significant difference compared with the DMSO control, *p < 0.05; **p < 0.01; ***p < 0.001; NS, not significant. (J) MMP9 reporter assay stimulated with Gli1, Gli2, ΔEN1, and MYC plasmid for 24 h in HEK293T cells. After that cells were treated with DMSO, SB4, and SB5. Data are presented as the mean ± standard deviation, *n* = 3. The asterisk indicates a significant difference compared with the DMSO control, *p < 0.05; **p < 0.01; ***p < 0.001; NS, not significant.

To identify the binding protein of SB4 and SB5, we synthesized linkers of DA analog (DF‐L1‐DF‐L8, SB5‐33) (Figure 2A). To find the appropriate linker position for SB4 and SB5 under the guidance of bioactivity, we investigated the effects of DF‐L compounds on spheroid formation. DF‐L1 and DF‐L8 are compounds with the same linker position with mutually corroborating inhibition ability. We tested these two linker compounds, which were successfully screened, in a spheroid formation experiment that reconfirmed them with SB4, SB5, and the compound SB5‐33, which had linker binding in the opposite direction. Results showed that DF‐L1 and DF‐L8 are correctly positioned linker compounds with similar inhibition activity as SB4 and SB5 (Figures 2B, S3, and S4). In the next step, CSC221 cell lysates were incubated with affigel beads to linker‐bound compounds (DF‐L1‐linker‐Affi‐Gel, DF‐L8‐linker‐Affi‐Gel) and probe‐bound proteins were pulled down and transferred to gel electrophoresis (Figure 2C). Coomassie brilliant blue G250 staining and silver staining were performed to visualize the bound proteins. The visualizations confirmed each other. Bands excised from Coomassie brilliant blue G 250 staining gel were isolated at the kDa marked on the Figure 2D and identified by peptide mass fingerprinting (PMF) analysis. Mass spectra are shown in Figures S5 and S6. PHB1, VDAC1, and MMP9, among the proteins identified to bind, were then confirmed by immunoblotting (Figure 2E). Protein sequencing coverage rates for identified proteins and matching peptides are shown in Figure 2F. Then, we then conducted an in silico study to confirm these results. Our results show that SB4 and SB5 bind to VDAC1, PHB, and MMP9 with similar binding scores (Figure 3A–F). We checked the expression of all bound proteins in CRC subtypes and in the different stage. *PHB1*, *VDAC1*, and *MMP9* were significantly overexpressed in tumorous tissues compared with normal tissues in CRC subtypes, whereas *PHB2* and *FAM20B* were not statistically significant. *PHB1*, *VDAC1*, *MMP9*, and *FAM20B* did not show statistical significance in the stages of cancer, but *PHB2* clearly showed decreased expression in advanced cancer types (stage III and stage IV). Furthermore, the disease‐free survival gradient for all targeted markers was lower in the high expression groups (Figure S7). To compare the expression of the target markers of SB compound in CRC, we checked their expression in The Human Protein Atlas (https://www.proteinatlas.org/), *PHB1*, *PHB2*, *VDAC1*, and *FAM20B* were generally expressed in CRC, whereas *MMP9* showed more marked differences in expression in some CRC cell lines (Figure S8).

Furthermore characterization of the binding proteins, we performed qRT‐PCR and Western blotting. *VDAC1*, *PHB1*, and *PHB2* decrease by a time‐dependent manner; however, *MMP9* dramatically decreases from early time points. We also wanted to confirm the mRNA expression of neighboring genes. While *TIM2* tended to be upregulated, *HK2* was significantly downregulated at 48 h of treatment (Figure 3G). After that, we check the protein level of MMP9, VDAC1, HK2, and PHB1. While MMP9, HK2, and PHB1 levels were downregulated at high concentration, no change was observed in VDAC1 protein level (Figure 3H). After that, we observed that MMP9 activity was downregulated by SB4 and SB5 in HEK293T cells transfected with the *MMP9* promoter plasmid (Figure 3I). Furthermore, we used a reporter assay to measure how molecules affect to MMP9 activity induced by the stem cell‐associated markers MYC, Gli1, Gli2, and ΔEN1 (Figure 3J). The results showed that while Gli2, ΔEN1, and MYC‐induced MMP9 activity was downregulated by the molecules, the activities of the compound were blocked in Gli1‐mediated MMP9 activity.

**FIGURE 4:**
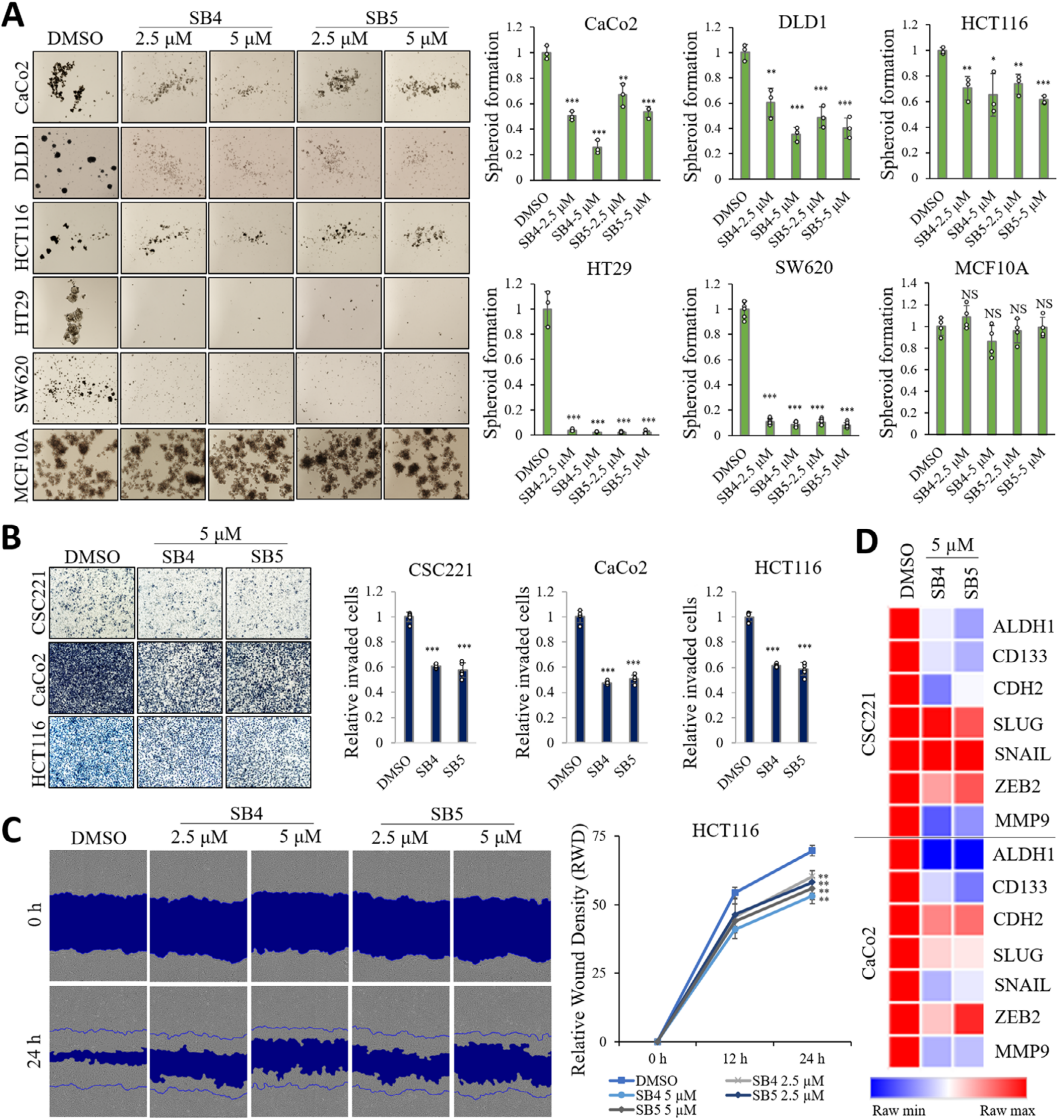
Inhibition of stemness and motility in colorectal cancer by VDAC1/PHB/MMP9‐binding compounds. (A) Representative images of spheroid formation by CRC cells (CaCo2, DLD1, HCT116, HT29, and SW620) and normal human breast cells (MCF10A) cells treated with SB4 and SB5 for 10–14 days, and quantitative analysis of the number of spheroids formed following each treatment. Data are presented as the mean ± standard deviation, *n* = 3–5. The asterisk indicates a significant difference between compared with the DMSO control, *p < 0.05; **p < 0.01; ***p < 0.001; NS, not significant. (B) Inhibitory effect on cell invasiveness by VDAC1/PHB/MMP9‐binding compound. After suspending 5–7 × 10^4^ CRC cells in medium containing 0.2% BSA, a Transwell invasion assay was performed. Cells were exposed to the compounds for 24 h at the 5 µM concentrations. The histogram represents invading cells. Data are presented as the mean ± standard deviation, *n* = 5. The asterisk indicates a significant difference between compared with the DMSO control, *p < 0.05; **p < 0.01; ***p < 0.001; NS, not significant. (C) To demonstrate the effects of VDAC1/PHB/MMP9‐binding compound on the motility of HCT116 cells, we performed a scratch wounding migration assay using the IncuCyte HD system. Cells were treated with SB4 and SB5 at 2.5 and 5 µM concentration for 24 h. Quantitative data of relative wound density in scratch wound migration assay using the IncuCyte system are given. Data are presented as the mean ± standard deviation, *n* = 3. The asterisk indicates a significant difference between compared with the DMSO control, **p < 0.01. (D) Heatmap displaying gene expressions related to stemness and EMT using data from the CSC221 and CaCo2 cancer cell line.

To test how these molecular changes are affected in different CRC cell lines, we performed spheroid formation testing. Our results showed that SB4 and SB5 at concentrations of 2.5 and 5 µM suppressed spheroid formation in different CRC cell line. In a noncancerous cell line, MCF10A, we observed that the treatment did not change the inhibition or induction of spheroid formation in a statistically significant way. These results are further evidence that the compounds have a specific effect on cancer (Figure 4A). In addition, we tested the activities of SB4 and SB5 in different cancer types. SB5 suppressed both MMP9 and spheroid formation in prostate cancer (RV1) cell line and breast cancer (MCF‐7) cell line differently than SB4 but both compounds did not affect the VDAC1 protein expression. SB4 and SB5 suppressed the spheroid formation of lung cancer (H1975) cell line, glioma (U87) cell line, and gastric cancer (AGS) cell line and, SB4 and SB5 suppressed the MMP9 protein level in the AGS (Figure S9). However, we compared compounds SB4 and SB5 with a known MMP inhibitor. Our results demonstrate that the MMP inhibitor (GM 6001) has no effect on inhibition of spheroid formation, revealing the potential of novel VDAC1/PHB/MMP9 binding compounds (Figure S10).

To evaluate the antimetastatic potential of our VDAC1/PHB/MMP9‐binding compounds, we first assessed their effect on the invasive capacity of CRC cells using gelatin coated Transwell assays. As shown in Figure 4B, treatment with the SB4 and SB5 significantly reduced the number of invaded cells compared with the vehicle‐treated control in CRC cells. Furthermore, we examined the impact of SB4 and SB5 on cell migration using a wound‐healing assay in HCT116 cells (Figure 4C). Time‐lapse imaging at 0 and 24 h revealed that compounds markedly impaired wound closure. To gain mechanistic insights into the observed phenotypes, we performed qRT‐PCR to analyze the mRNA expression of selected genes associated with stemness (*ALDH1*, *CD133*) and epithelial‐mesenchymal transition (EMT: *CDH2*, *SLUG*, *SNAIL*, *ZEB2*). MMP9, a key ECM‐degrading enzyme implicated in invasion and metastasis, was also transcriptionally suppressed by compounds. The heatmap illustrates relative mRNA expression changes in response to treatment with SB4 and SB5 (Figure 4D). These results suggest that SB4 and SB5 exert their antimetastatic effects by downregulating stemness and EMT‐related genes, thereby impairing the invasive and migratory abilities of CRC cells.

### 2.3 VDAC1/PHB/MMP9 Targeted Therapy Suppresses Mitochondrial Respiration, Induces ROS and Apoptosis

**FIGURE 5:**
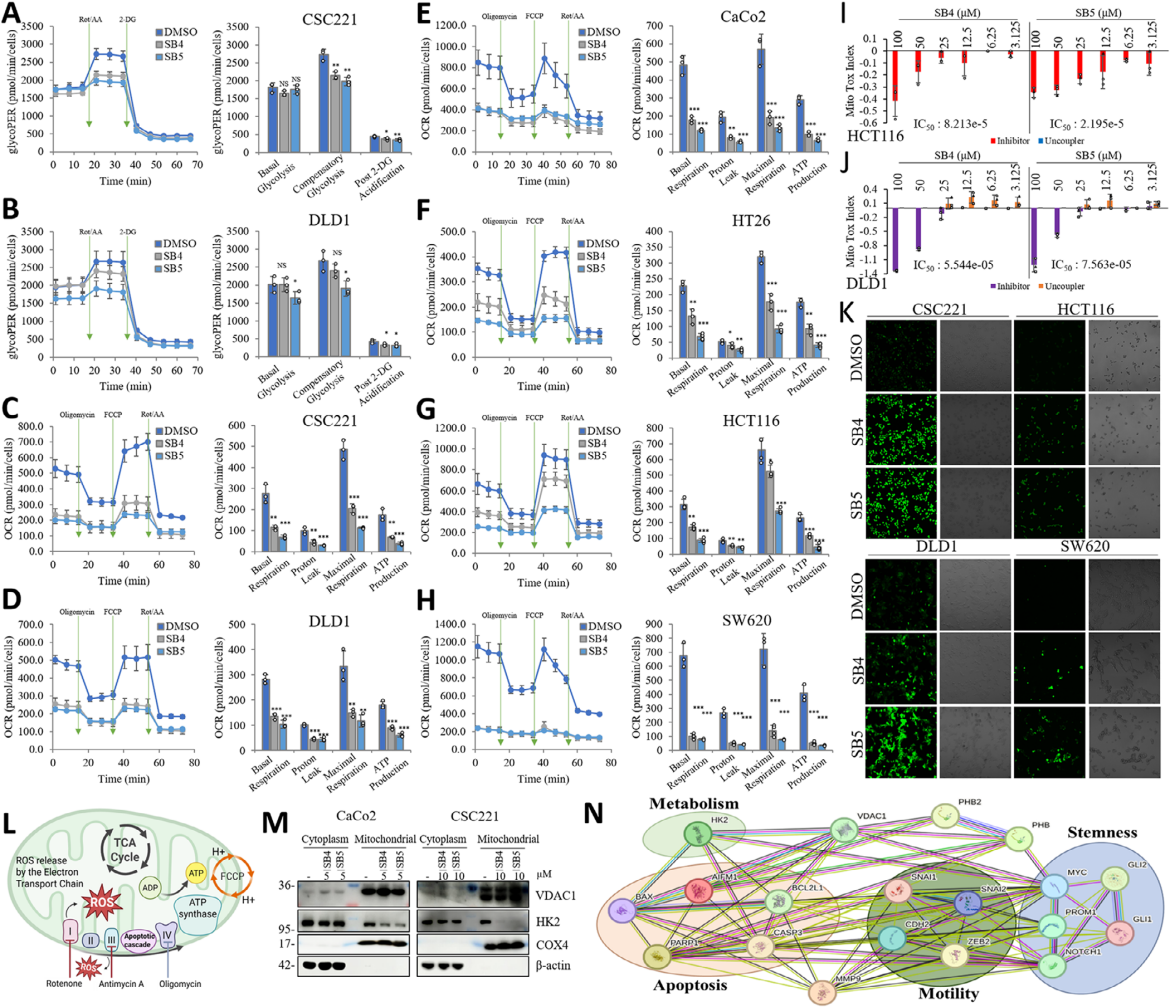
Synthesized molecules that meet at the VDAC1/PHB/MMP9 crossroads control ROS generation and ATP production by regulating the electron transport chain. (A and B) The extracellular acidification rate (ECAR) and oxygen consumption rate (OCR) were detected using the Glycolytic Rate Assay Kit on a Seahorse XF96 extracellular flux analyzer. CSC221 and DLD1 cells were treated with the SB4 (5 µM) or SB5 (5 µM) for 48 h. To measure glycolytic rates, the assay utilizes both ECAR and OCR measurements to determine the glycolytic proton efflux rate (glycoPER). glycoPER was measured at two‐time points, followed by sequential injection of rotenone/antimycin A, and 2‐DG. Basal and compensatory glycolysis and post‐2‐DG acidification parameters were given in the figure. Data are presented as the mean ± standard deviation, *n* = 3. The asterisk indicates a significant difference between compared with the DMSO control, *p < 0.05; **p < 0.01; ***p < 0.001; NS, not significant. (C–H) OCR was detected using the Cell Mito Stress Test Kit on a Seahorse XF96 extracellular flux analyzer. CSC221, DLD1, CaCo2, HT29, HCT116, and SW620 cells were treated with the SB4 (5 µM) or SB5 (5 µM) for 48 h. OCR was measured at three‐time points, followed by sequential injection of oligomycin, FCCP, and rotenone/antimycin A to assess basal respiration, proton leak, maximal respiration, and ATP production, respectively. Data are presented as the mean ± standard deviation, *n* = 3–4. The asterisk indicates a significant difference between compared with the DMSO control, *p < 0.05; **p < 0.01; ***p < 0.001; NS, not significant. (I and J) A dose–response investigation of mitochondrial toxicity of SB4 and SB5 at indicated concentrations (100–3.125 µM) in HCT116 and DLD1 cell lines. Kinetic OCR was measured by Seahorse XF96 Pro extracellular flux analyzer by using the Mito Tox assay kit. MTI (Mito Tox Index) values were assessed across a range of SB4 and SB5 concentrations, revealing dose‐dependent toxicity profiles and corresponding IC_50_ values. The graph also shows the MTI values of the uncoupler dose response. Data are presented as the mean ± standard deviation, *n* = 3. (K) Cells were treated with SB4 (10 µM) or SB5 (10 µM) for 12 h for ROS generation. ROS was detected by fluorescence microscopy using DCFH‐DA (10 µM) in CSC221, HCT116, DLD1, and SW620 cell lines. (L) Representative illustration of the domain of reagents used in bioenergetic experiments and mitochondrial ROS production. (M) VDAC1 protein levels in mitochondrial fraction and downregulated HK2 protein levels in both cytosolic and mitochondrial fraction upon 10 µM of SB4 or SB5 treatment. Western blots were repeated three times. (N) To investigate the act area of VDAC1/PHB/MMP9 binding compound, the network of protein–protein interactions associated with metabolism, apoptosis, stem cells, and motility was investigated (STRING; https://stringdb.org).

Seahorse XF glycolytic rate assay was used for calculated basal glycolysis and compensatory glycolysis. SB4 (5 µM) or SB5 (5 µM) treatment suppresses the glycolysis in light manner (Figure 5A,B). Furthermore, we used the Seahorse XF Mito Stress Kit and Mito Tox Kit tests to investigate mitochondrial respiration and toxicity. Many kinds of mitochondrial function can be modulated by the molecules. These mitochondrial functions include TCA cycle enzymes, ATP synthase, and other oxidative phosphorylation components, electron transport chain (ETC) protein complexes, mitochondrial transcription, and various mitochondrial transporters. SB4 (5 µM) or SB5 (5 µM) treatment dramatically suppresses the oxygen consumption rate (OCR) in the CSC221, DLD1, CaCo2, HT29, HCT116, and SW620 cells. Basal respiration, proton leak, maximal respiration, and ATP production are parameters of this assay, treatments significantly suppress the parameters in all CRC cell lines (Figure 5C–H). Mitochondrial toxicity experiment was performed with HCT116 and DLD1 cells. A negative MTI score (usually between 0 and –1) is caused by mitochondrial toxicity due to inhibition, which is defined and observed as a decrease in FCCP OCR by the test compound compared with the maximal FCCP OCR of the vehicle group. While the compounds showed an inhibitory effect on HCT116. A positive MTI score (usually between 0 and 1) is caused by mitochondrial toxicity caused by uncoupling, which is characterized and measured as an increase in the test compound's Oligo OCR relative to the vehicle group's minimal Oligo OCR. Moreover, DLD1 cells were shown an uncoupling effect at low concentrations (Figure 5I,J). We next examined how ROS production responded to the decline in mitochondrial respiration. CSC221, HCT116, DLD1, and SW620 cells increased ROS production after treatment (Figure 5K). In order to maintain proliferative and metastatic signals in cancer cells, mitochondria regulate redox homeostasis through Complex I and III activities in the ETC [26] (Figure 5L). Moreover, treatment approaches that interfere with the atypical mitochondrial metabolism include blocking the connection between hexokinase 2 (HK2) and VDAC [27]. Our results showed that mitochondrial VDAC1 trendily upregulated and mitochondrial HK2 dramatically decreased in SB4 and SB5‐treated cells (Figure 5M). We created a network of linked tokens at the VDAC1/PHB/MMP9 crossroad (Figure 5N). Important players in the field of stemness include MYC, Gli1, Gli2, Notch1, and CD133 (PROM1) [18, 28, 29]. Bax, CASP3 (caspase 3), PARP1, AIFM1, and BCL2L1 control the thin line that separates life from death. Overlaying both metabolism and apoptosis, HK2 performs a delicate dance that controls both the dynamics of cellular energy and the precarious equilibrium between life and death [30]. *CDH2*, *ZEB2*, *SNAI2*, and *SNAI1* are important regulatory genes that increase the motility and invasion capacity of cancer cells. These transcription factors trigger processes such as cytoskeletal reorganization, disruption of cell polarity, and dissolution of adhesion complexes, leading to a more motile and aggressive phenotype. These molecules, particularly those associated with the EMT, promote the spread of tumor cells into surrounding tissues and the ability to metastasize to distant organs [31, 32, 33].

**FIGURE 6:**
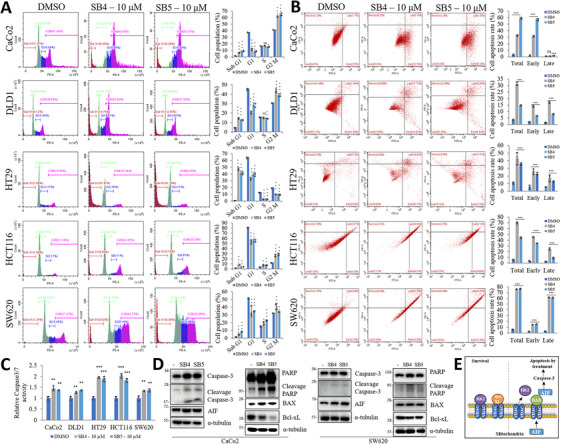
SB4 and SB5 exert cell cycle disruption and apoptotic cell death by VDAC1/HK2 interaction blockade in the mitochondria. (A) Using flow cytometry, the cell‐cycle distribution of CaCo2, DLD1, HT29, HCT116, and SW620 cells. Cells were treated with SB4 or SB5 (10 µM) for 48 h. Quantitative measurements of the sub‐G1, G1, S, and G2/M populations were given in the graph. Data are presented as the mean ± standard deviation, *n* = 3. The asterisk indicates a significant difference compared with the DMSO control, *p < 0.05; **p < 0.01; ***p < 0.001; NS, not significant. (B) To analyze necrotic and apoptotic cell populations using a CytoFLEX Flow Cytometer, cells were stained with Annexin V–FITC/PI. Quantification of the percentage of total, early, and late apoptotic cells treated with the indicated compound for 48 h. Data are presented as the mean ± standard deviation, *n* = 3. The asterisk indicates a significant difference compared with the DMSO control, *p < 0.05; **p < 0.01; ***p < 0.001; NS, not significant. (C) CRC cells treated with the SB4 or SB5 at 10 µM for 48 h. After that caspase 3/7 activity was determined with a Caspase‐Glo 3/7 assay kit. Results are given relative to DMSO. Data are presented as the mean ± standard deviation, *n* = 3–4. The asterisk indicates a significant difference compared with the DMSO control, *p < 0.05; **p < 0.01; ***p < 0.001; NS, not significant. (D) Western blot study of the apoptosis proteins PARP, caspase‐3, Bax, Bcl‐XL, AIF in SB4 or SB5‐treated CaCo2 and SW620 cells for 48 h. Tubulin acted as a loading control. Western blots were repeated three times. (E) According to the known information, the N‐terminal region mobility functions as an interaction site for hexokinase (HK) and apoptosis‐regulating proteins of the Bcl‐2 family, including Bax, Bcl‐2, and Bcl‐xL, as well as channel gating and interaction with antiapoptotic proteins and VDAC1 dimer formation [25]. The illustration summarizes the literature and our finding information for the interpretation of the SB compound's mechanism on apoptosis.

To furthermore characterize the response of direct binding to VDAC1/PHB/MMP9 in cancer cells, we examined the effects of CRC cells on cell death and cell cycle. We determined cell cycle regulation by SB4 and SB5 on CRC cells. SB4 and SB5 induced Sub G1 and G2/M populations while G1 population decreased on all the CRC cell lines (Figure 6A). We have also shown in Figure S11 that SB4 and SB5 regulate cell cycle in other cancer cell lines, and we also observed that in noncancerous cell lines, treatments decreased sub G1 population and increased G1 accumulation. This finding suggests that in noncancerous cell lines, SB4 and SB5 either indicate that cells are protected them from entering the apoptosis phase. Then, to determine apoptotic cell death, we subjected the cells treated with SB4 and SB5 to Annexin V–PI and Annexin V–FITC staining and analyzed them by flow cytometry. Compounds were induced in the apoptotic cell population in the CRC cell lines after 48‐h treatment at 10 µM concentration (Figure 6B). Furthermore confirmation, we performed caspase 3/7 activity to confirm whether apoptotic cell death is regulated by caspase dependent. Results showed that caspase 3/7 activity was induced by the SB4 and SB5 treatment in different cell lines (Figure 6C). We used western blot analysis to measure the levels of apoptosis‐related proteins (PARP, caspase‐3, BAX, Bcl‐xL, AIF) in order to verify the induction of apoptosis. As a shown in the Figure 6D, SB4 and SB5 induced caspase‐3, cleaved caspase‐3, PARP, Bax, AIF and decreased the Bcl‐xL protein level on CaCo2 and SW620 cells (Figure 6D). Overall, our results showed that SB4 and SB5 have cell death ability at ∼10 µM concentration on CRC cell lines (Figure 6E).

### 2.4 Cotreatment with SB4 or SB5 Reverses the Promoting Effects of Cetuximab Against Human Colorectal Adenocarcinoma‐Enriched CSCs

**FIGURE 7:**
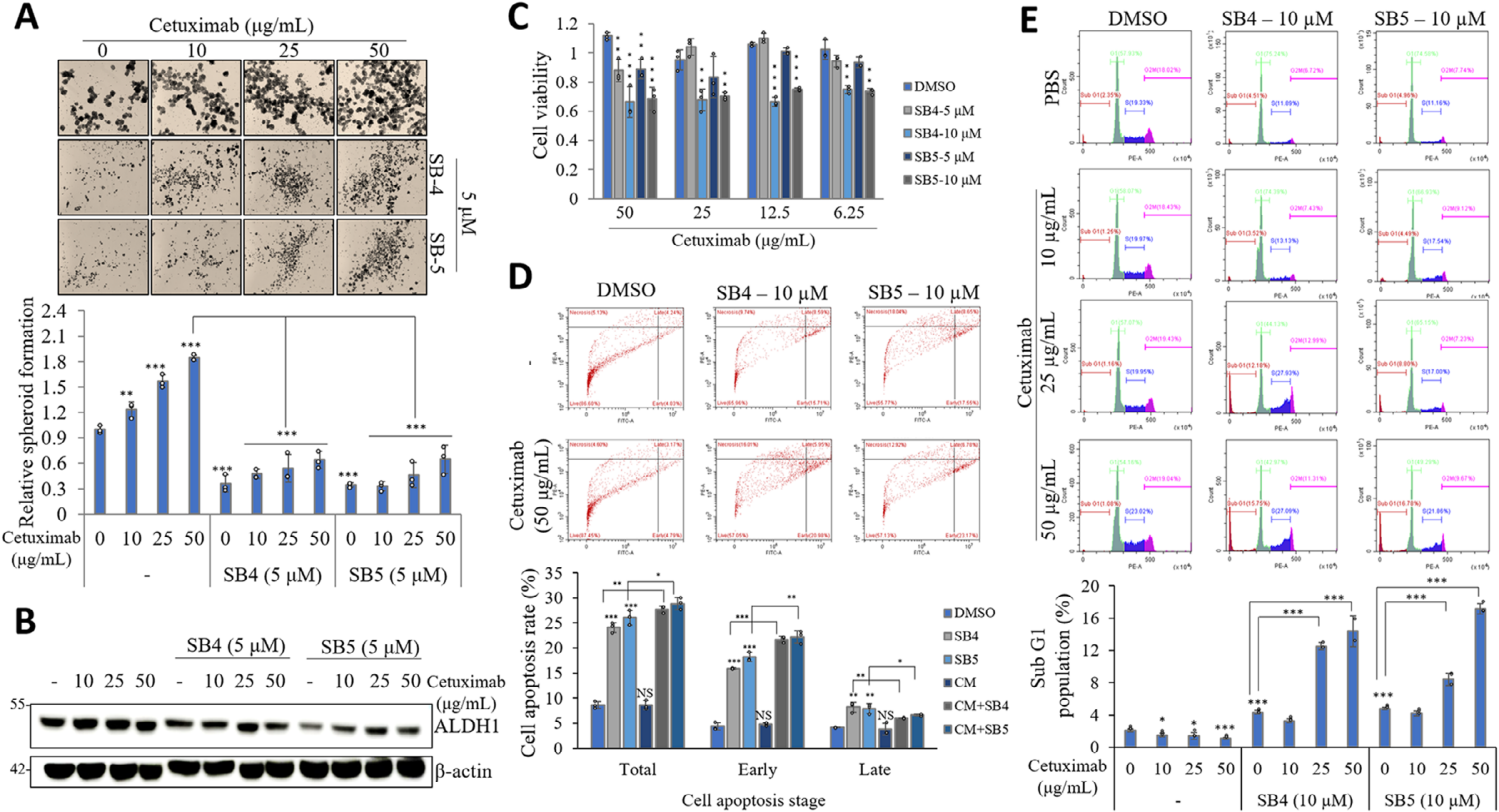
Treatment of cetuximab, which has a promotion effect on the CSC221 cell line, together with SB4 or SB5 eliminates these effects. (A) Representative images of spheroid formation by CSC221 cells treated with cetuximab (10, 25, and 50 µg/mL) and SB4 (5 µM) or SB5 (5 µM) for 2 weeks, and quantitative analysis of the number of spheroids formed following each treatment. Data are presented as the mean ± standard deviation, *n* = 3. The asterisk indicates a significant difference between treatment groups, *p < 0.05; **p < 0.01; ***p < 0.001; NS, not significant. (B) CSC221 cell line treated with indicated concentration of cetuximab with or without SB4 and SB5 for 48 h. After that cell lysates were subjected to the SDS‐PAGE for ALDH1 protein level. Western blots were repeated three times. (C) CSC221 cells were treated with cetuximab and SB4 or SB5 at the indicated concentration. Cell viability was measured by MTT assay. Cell viability was given relative to the DMSO control group. Data are presented as the mean ± standard deviation, *n*  = 3. *p < 0.05; **p < 0.01; ***p < 0.001; NS, no significant difference between compared groups. (D) Annexin V–FITC/PI was used to label cells for analysis of apoptotic cell populations using a CytoFLEX Flow Cytometer. The percentage of total, early, and late apoptotic cells was quantified after 48 h of indicated compounds with or without combination treatment. Data are presented as the mean ± standard deviation, *n* = 3. The asterisk indicates a significant difference between treatment groups, *p < 0.05; **p < 0.01; ***p < 0.001; NS, not significant. (E) Using flow cytometry, the cell cycle distribution of CSC221 cells treated with cetuximab, SB4, SB5, and the indicated combinations for 48 h was assessed. Quantitative measurements of sub‐G1 population are given in the graph. Data are presented as the mean ± standard deviation, *n* = 3. The asterisk indicates a significant difference between treatment groups, *p < 0.05; **p < 0.01; ***p < 0.001; NS, not significant.

Here, we investigated the response of cetuximab, an EGFR‐targeted therapy approved by the United States Food and Drug Administration in 2004 [34], in combination with SB4 or SB5 in the CSC221 cell line. The results on spheroid formation were quite surprising, as single treatment with cetuximab significantly induced spheroid formation of CSC221 cells. Cotreatment of SB4 or SB5 with cetuximab significantly reduced the adverse effects of cetuximab and even preserved the strong effects of SB4 and SB5 on spheroid formation compared with control (Figure 7A). We then analyzed the protein level of ALDH1, a stemness marker, to evaluate the formation of the spheroid formation. ALDH1 level was induced by cetuximab treatment, SB4 and SB5 treatment suppressed the induced levels in the CSC221 cell line (Figure 7B). Moreover, treatment of cetuximab alone did not show cytotoxic effects on CSC221 cells, while treatment of SB4 or SB5 with cetuximab triggered the cytotoxic ability of the components (Figure 7C). These findings were further validated by an apoptosis assay employing Annexin V–FITC/PI double staining, which demonstrated that treatment with SB4 or SB5 in combination with cetuximab significantly increased early apoptotic cell death (Figure 7D). Likewise, in the cell cycle test performed by flow cytometry, while the Sub G1 population tends to decrease with single cetuximab treatment in a dose‐dependent manner. However, SB4 or SB5 treatment with cetuximab creates a more effect and causes induction in the Sub G1 population (Figure 7E). The therapeutic effect of cetuximab varied depending on KRAS status: no benefit was observed in patients harboring mutant KRAS, while patients with wild‐type KRAS derived advantage from the treatment [35]. Therefore, we tested the response of the CaCo2 cell line, known as *KRAS*‐wild type, to cetuximab and cetuximab + SB4 or SB5 in the formation of spheroid formation. Similarly to CSC221 cells, cetuximab‐induced spheroid formation in CaCo2 cells was effectively suppressed in groups cotreated with SB4 and SB5 (Figure S12). Furthermore, we examined cell cycle modulation by cetuximab and the combination of cetuximab with SB4. Cotreatment with SB4 and cetuximab synergistically induced an increase in both the Sub‐G1 and G2/M populations (Figure S13).

### 2.5 Pharmacokinetic Characterization and Tissue Distribution Profile of VDAC1/PHB/MMP9‐Binding Compounds

**FIGURE 8:**
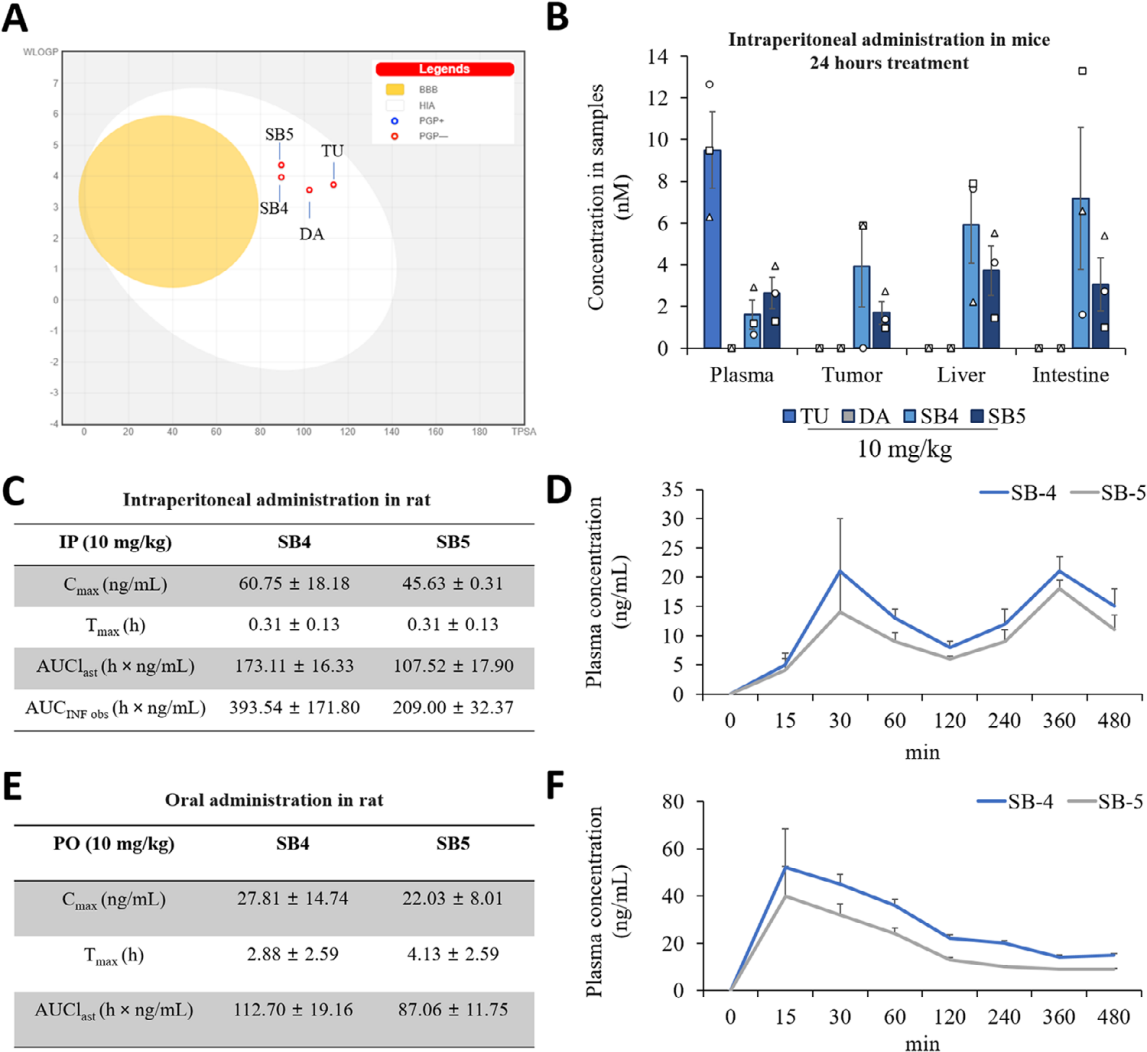
In silico pharmacokinetic profiling, tissue distribution, and plasma PK parameters of SB4 and SB5. (A) In silico pharmacokinetic analysis of compounds. The graph represents the WLOGP versus TPSA for SB4, SB5, TU, and DA. The yellow area represents the region of BBB permeability, while white represents HIA. Red circles indicate P‐gp‐negative compounds—meaning minimum eflux risk. Thus, SB4 and SB5 had optimal HIA with low P‐gp interaction and are promising candidates against CRC. (B) Tissue distribution profile of compounds TU, DA, SB4, and SB5 after 24 h at 10 mg/kg dose. Levels of each compound were measured in plasma, tumor, liver, and intestine samples. Tumors were generated with 1 × 10^6^ CT26 cells for 2 weeks. Data are presented as the mean ± standard error, *n* = 3. (C and D) Plasma concentration–time curves in Sprague–Dawley rats following intraperitoneal administration of SB4 and SB5 (10 mg/kg). Data are presented as mean ± standard deviation in panel C and as mean ± standard error in panel D, *n* = 4, with samples collected at predose and 15, 30, 60, 120, 240, 360, and 480‐min postdose. Pharmacokinetic parameters derived from these profiles include indicated parameters. (E and F) Plasma concentration–time curves in Sprague–Dawley rats following a single oral gavage of SB4 or SB5 at 10 mg/kg. Data are presented as mean ± standard deviation in panel E and as mean ± standard error in panel F, *n* = 4, with samples collected at predose and 15, 30, 60, 120, 240, 360, and 480‐min postdose.

Regarding the in silico pharmacokinetic profiles of the compounds targeting CRC, both SB4 and SB5 demonstrate favorable characteristics. As illustrated in Figure 8A, both compounds fall within the white ellipse, indicating high potential for human intestinal absorption (HIA). Additionally, the red circles signify that neither SB4 nor SB5 are substrates of P‐glycoprotein (P‐gp), suggesting efficient cellular uptake and a reduced likelihood of efflux‐mediated excretion. SB4, in particular, exhibits a well‐balanced profile in terms of WLOGP and TPSA, supporting its suitability for oral administration. Although both compounds lie outside the yellow region associated with blood–brain barrier (BBB) permeability, this is not a limitation in the context of CRC, which does not involve the central nervous system. When compared with TU and DA, SB4 and SB5 possess more advantageous pharmacokinetic properties and lower efflux risks, indicating their potential as more effective therapeutic agents for CRC.

Plasma, tumor, intestinal, and liver tissues were then collected from Balb/c mice treated with the four compounds 24 h later and drug distribution was analyzed. The low quantities of SB4 and SB5 in plasma (less than 2 nM) may suggest that these compounds have a propensity to enter target tissues rapidly and stay in plasma for shorter periods of time. Quick removal from the bloodstream could help minimize adverse effects and off‐target consequences. SB4 and SB5 are present in the liver at concentrations of approximately 1–2 nM. This indicates that both compounds are retained in the liver to some extent for biotransformation or metabolism, but do not tend to accumulate. Therefore, it can be said that the potential risk of toxicity in the liver may be low. The distribution of synthesized SB4 and SB5 compounds in tumor and intestines indicates that their bioavailability is improved compared with TU and DA. In particular, their distribution in intestinal tissue suggests that these compounds may be more suitable treatment options for intestinal‐orientated diseases (Figure 8B).

The pharmacokinetic properties of SB4 and SB5 were investigated after intraperitoneal administration at a dose of 10 mg/kg. *C*_max_ of SB4 was higher (60.75 ± 18.18 ng/mL) compared with SB5 (45.63 ± 0.31 ng/mL), while *T*_max_ of both compounds was comparable at 0.31 ± 0.13 h. The area under the plasma concentration–time curve from 0 to the last measurable concentration and extrapolated to infinity was also higher for SB4: 173.11 ± 16.33 h × ng/mL for AUCI_ast_ and 393.54 ± 171.80 h × ng/mL for AUC_INF obs_ versus the values after administration of SB5: 107.52 ± 17.90 h × ng/mL for AUC_ast_ and 209.00 ± 32.37 h × ng/mL for AUC_INF obs_ (Figure 8C,D). Following oral administration of SB4 and SB5 (10 mg/kg) in rats, plasma concentration–time profiles were evaluated. Both compounds showed rapid absorption with peak plasma concentrations (*C*_max_) observed within 30 min. SB4 displayed a slightly higher *C*_max_ and AUC compared with SB5, suggesting improved oral bioavailability (Figure 8E,F).

### 2.6 VDAC1/PHB/MMP9‐Binding Compound Suppresses Tumor Growth of CRC Xenograft in Vivo

**FIGURE 9:**
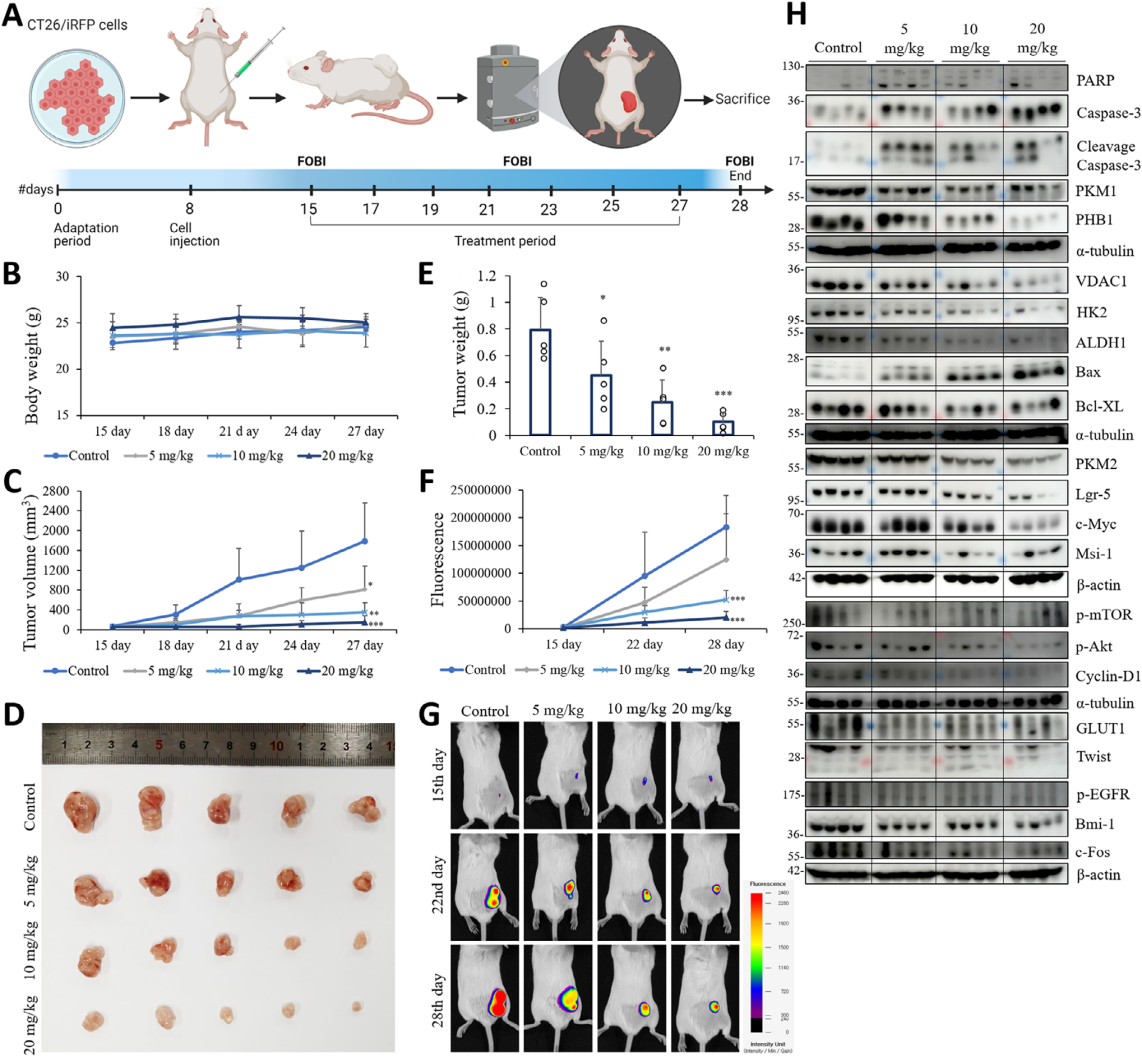
VDAC1/PHB/MMP9‐binding compound effectively suppresses the tumor growth. (A) Experimental timeline of CT26/iRFP cells in vivo study. Each mouse was implanted with 1 × 10^6^ CT26 cells beneath the skin. Subsequently, mice were administered SB4 intraperitoneally at doses of 5, 10, and 20 mg/kg, with injections given every other day. (B) Body weight of control and treated mice. (C) Tumor volumes. (D) Tumors after subcutaneous injection of CT26 cells. (E) Tumor weight. (F) The fluorescence area of tumors in mice, measured using the FOBI (fluorescence‐labeled organism bioimaging instrument) system, shows dose‐dependent tumor growth inhibition across different time points. (G) Representative FOBI images of mice from the control and treatment groups on days 15, 22, and 28. The fluorescence signal intensity, depicted by a heat map ranging from low to high, demonstrates the inhibitory effect of increasing compound doses on tumor growth. Data are presented as the mean ± standard deviation, *n* = 5. *p < 0.05; **p < 0.01; ***p < 0.001; NS, not significant versus the DMSO‐treated group. (H) The protein levels of PARP, caspase, cleavage caspase‐3, PKM1, PHB1, VDAC1, HK2, ALDH1, Bax, Bcl‐XL, PKM2, Lgr‐5, c‐Myc, Msi‐1, p‐mTOR, p‐Akt, cyclin D1, GLUT1, Twist, p‐EGFR, Bmi‐1, and c‐Fos were examined via immunoblot analyses tumors collected from control and SB4‐treated groups (5, 10, 20 mg/kg). Blots shown protein samples from four different tumor tissues from each treatment group.

Before CT26‐derived mouse model, we first observed downregulated changes in several biomarker (ALDH1, β‐catenin, c‐Myc, PHB1) levels to determine the activity of SB4 and SB5 compounds in the CT26 cell line (Figure S14). After that, to assess the in vivo efficacy of the VDAC1/PHB/MMP9‐binding compound, a xenograft model was established by inoculating mice with CT26–iRFP cells (Figure 9A). One week after inoculation, mice bearing tumors were treated intraperitoneally with either vehicle control (DMSO) or different doses of SB4 (5, 10, or 20 mg/kg) every other day. As indicated in Figure 9B–E, SB4 treatment significantly reduced both tumor volume and weight in a dose‐dependent manner compared with the control group. Moreover, we showed that the treatment with SB4 did not influence mouse body weight; this indicated the absence of toxic effects. To further confirmation bioimaging by the fluorescence‐labeled organism bioimaging instrument (FOBI) system was performed to visualize the tumor mass in each mouse in relation to fluorescence intensity Figure 9F,G. The results confirmed the dose‐dependent effect.

Furthermore investigation, ex vivo protein analysis shows a dose‐dependent effect due to the treatment that is evident at 10 and 20 mg/kg, showing clear induction of apoptosis following the increase in cleaved caspase‐3 and Bax levels; on the contrary, there was a decrease in the antiapoptotic expression of Bcl‐XL. While markers of proliferation, such as c‐Myc and Cyclin D1, are decreased at higher concentrations, suggesting an inhibition of cell growth, survival pathways, as evidenced by decreased levels of p‐mTOR, p‐Akt, p‐EGFR, and c‐Fos. The expression of stem cell markers such as ALDH1, Lgr5, Msi‐1, and Bmi‐1 was also reduced at higher concentrations, possibly relating to an alteration in some aspect of stem cell dynamics. Moreover, protein levels of metabolic markers PKM1, PKM2, and GLUT1, including HK2, which is both a proapoptotic marker and a metabolic marker, are reduced compared with the control group. In addition, the protein level of the Twist marker responsible for EMT transcription decreased in the treatment groups. The in vivo efficacy of VDAC1/PHB/MMP‐binding compounds was also confirmed by ex vivo data (Figure 9H). Unlike in vitro based conditions, we observed downregulation of VDAC1 level here. In addition, levels of PHB decreased in a dose‐dependent manner. In summary, this VDAC/PHB/MMP9‐binding compound treatment causes apoptosis and inhibits proliferation, stemness, motility, and metabolism.

## 3 Discussion

Lichens are unique compared with higher plants and show interesting biological and pharmacological activities [36, 37]. A kind of polyphenolic molecule known as a depside is produced by esterifying two (didepsides) or three (tridepsides) or more orcinol units. These kinds of compounds are created when two or more hydroxybenzoic acids condense and the carboxyl group of one molecule is esterified with the phenolic hydroxyl group of another molecule. The wide range of biological activities of depside‐structured compounds stand out in reported studies [16, 18, 38]. In this study, we derived analogs based on lichen secondary metabolites constructed in two depsides. One of them, DA, is a derivative of depside and possesses cytotoxic, analgesic, antipyretic, and antitumoral qualities [39]. Another depside lichen secondary metabolite, TU, exhibited antistemness properties in CRC [17]. We aimed to synthesize more compounds and evaluate stronger activity to see the structure–activity relationship with the desired compound. That is why, we attach the linker to the o‐ortho hydroxy group and replace the linker part. Here, we identified compounds SB4 and SB5 as hit compounds and identified the proteins they directly target by creating linker‐conjugated bead structures. Our findings were as follows: (1) Hit compounds (SB4 and SB5) show stronger selective cytotoxic and antistemness activities than the original compounds from which they are derived; (2) compounds (SB4 and SB5) directly bind to the VDAC1, PHB1, PHB2, and MMP9; (3) SB4 and SB5 induce cell death by weakening the expression of the proapoptotic marker HK2 associated with the binding proteins and inducing *caspase 3* and *PARP*; (4) SB4 and SB5 suppress mitochondrial respiration, while triggering ROS production in CRC cell lines; (5) combination treatment with SB4 or SB5 reverses the stimulating effects of cetuximab on human colorectal adenocarcinoma‐enriched CSCs and *KRAS*‐wild type CRC cell line; (6) SB4 and SB5 exhibit pharmacokinetic and tissue distribution properties as viable treatment options for CRC; (7) the efficacy of the VDAC1/PHB/MMP9 binding compound was demonstrated in vivo. Collectively, our results demonstrate that structure and bioactivity‐guided synthesized new compounds intersect at the VDAC1/PHB/MMP9 crossroads and exhibit cancer‐specific cytotoxic properties, inducing apoptosis and suppressing the energy metabolism and stemness of CRC.

Mitochondria is an important organelle that plays a role in important vital processes from life to death. The electron transport system modulates cellular processes, including ATP and ROS production and cellular signaling [40]. In cancer, mitochondrial physiology inhibits the accumulation of ROS and prevents the triggering of ROS‐mediated cancer progression [41]. VDAC is a one of the multifunctional mitochondrial protein, as a acting cell life/cell death, energy metabolism, and stemness. Additionally, VDAC1 controls apoptosis through its interactions with Bcl‐xL, hexokinase, and other apoptosis‐regulating proteins, some of which are also highly expressed in a number of malignancies. Therefore, VDAC1 represents an emerging cancer drug target [25]. Reported study have revealed that VDAC1 is associated with prohibitin (PHB). The membrane proteins known as PHBs (PHB1 and 2) are found in mitochondria as multimeric ring complexes and are very conserved. They are engaged in a number of vital functions, including biogenesis, mitochondrial DNA preservation, cell survival, apoptosis, and sculpting of the mitochondria [42, 43]. Also, PHB localized in the cell membrane, nuclear, and cytoplasm [44]. The c‐KIT/phospho‐PHB axis has been reported to increase ovarian cancer stemness and chemoresistance via Notch3–PBX1 and β‐catenin–ABCG2 signaling [45]. In addition, the promotion of cellular metastasis by Raf activation by Ras is reported to require phosphorylation of PHB in the raft domain of the plasma membrane [46]. A study conducted in prostate cancer showed that PHB may have a nuclear regulatory role in cell cycle progression [47]. To put it succinctly, PHBs integrate a multitude of signaling pathways, including Akt, C‐RAF–MEK–ERK, CaMK, to regulate a range of cellular physiological processes, including as transcription, metabolism, apoptosis/survival, cytoskeletal assembly, and differentiation [43]. Our study shows that future studies need to elucidate the potential mechanisms between VDACs, PHBs, and MMPs. However, a study has linked the expression of prohibitinin with the expression of MMP9 and ERK signaling in gallbladder cancer [48]. Among the MMPs that have been studied the most, MMP9 is an important protease that is essential to several biological processes such as rearrange on the composition of niches, apoptosis, nonproteolytic manner via their hemopexin domain, proliferation, tissue invasion, genetic changes in MMPs linked the cancer. Several ECM proteins can be cleaved by MMP9 to control the remodeling of the ECM [49, 50]. Therefore, cotargeting VDAC1/PHB/MMP9, which is involved in cell death, stem cell, and metabolic crossroads, may be a new promising approach in the treatment of CRC.

Our data suggest that the synthesized compound engages VDAC1, PHB, and MMP9, as evidenced by pull‐down assays and mass spectrometry. This interaction leads to reduced mRNA and protein levels of the targets. The downregulation may result from disrupted transcriptional feedback loops VDAC transcription is sustained by NRF‐1 and HIF‐1α [51], while PHB2 modulates transcription via MyoD and MEF2 [52]. Compound binding may interfere with these regulatory pathways. Additionally, conformational changes induced by the compound can expose degradation motifs or misfold proteins, enhancing ubiquitination and proteasomal degradation. VDAC1 and MMP9 are known substrates of E3 ligases such as TRIM31, Parkin, or UBE2C [53, 54, 55]. Posttranscriptional mechanisms may also be affected; for instance, compound treatment could disrupt RNA‐binding proteins like HuR and HuB that stabilize MMP9 mRNA [56] or alter microRNA‐mediated regulation of VDAC1 [57]. Taken together, these mechanisms suggest a functional link between compound binding and suppression of target expression. This multilevel inhibition likely contributes to the observed anticancer effects and warrants further mechanistic investigation.

Disruption of cellular signaling pathways is directly related to the pathogenesis of CRC [58]. Hedgehog signaling, Notch, Wnt, Myc, EGFR/MAPK, PI3K/AKT/PTEN, and STAT signaling pathways play key roles in the development and progression of CRC [59]. A important stemness signaling of Wnt signaling pathway abnormal activation is associated with mutations in tumor suppressor genes such as APC (adenomatous polyposis coli) or other mutations affecting the complex that cleaves β‐catenin [60]. One of the stemness marker leucine‐rich repeat‐containing G protein‐coupled receptor 5 (Lgr‐5) related with the Wnt/β‐catenin signaling pathway and promotes the EMT and metastasis [61]. A crucial route in the development of CRC stemness is the SHH pathway. The beginning of cell growth, differentiation, and tissue patterning are all dependent on the embryonic development factor SHH. Abnormal activation of this pathway has been demonstrated to have a role in the growth, survival, and metastasis of cancer cells [62, 63]. Furthermore, many kind of stemness markers play role for the progression of CRC such as CD44, CD133, Msi‐1, Bmi‐1, and ALDH1 [60]. High ALDH1 expression in colon cancer tissues is associated with poor differentiation, an advanced tumor stage, and metastasis [64]. VDAC1/PHB/MMP9‐binding compounds (SB4 and SB5) reversed activated cancer stemness markers and related signaling pathways.

Our findings indicate that the synthesized depside molecules interact with VDAC1/PHB/MMP9; however, we cannot exclude the possibility that their anticancer effects may also involve additional targets. Further investigations using advanced proteomics or other unbiased screening approaches could provide deeper insights into their target specificity and broader mechanisms of action. However, RNA sequencing (RNA‐seq) and spatial biology‐based approaches should be integrated to further investigate the broader interaction networks and biological contexts of target molecules.

Pharmacokinetic and tissue distribution profiles of SB4 and SB5 underline that both compounds are potential therapeutics for CRC as novel synthetic compounds with improved pharmacokinetic and tissue distribution profiles compared with TU and DA through in silico prediction and in vivo assays. Although these findings are based on early preclinical models, future work will be needed to determine long‐term pharmacokinetics, metabolism, and potential toxicity in higher‐order systems. Advancing formulation technologies, including nanoparticle delivery and prodrug design, may further improve the pharmacological profile and clinical applicability of VDAC1/PHB/MMP9‐binding compounds.

In summary, we introduced that structurally optimized novel synthetic depsides (SB4 and SB5) bind to the VDAC1/PHB/MMP9 can effectively interfere with key hallmarks of CRC. This molecular crossroad is a strategic target by SB4 and SB5 to suppress stemness, cell motility, metabolism, mitochondrial function, and survival signaling in tumor cells. Therefore, cotargeting VDAC1/PHB/MMP9 also offers translational potential to develop patient‐specific therapies. This approach may contribute to overcoming chemotherapy resistance and distant metastasis, particularly in CRC, and may be adapted for precision oncology strategies across multiple tumor types.

## 4 Materials and Methods

### 4.1 Synthesis of SB Compounds

All reactions were performed under proper conditions as stated. Commercially available reagents and solvents were used for synthesis without further purification. Completion of the reaction was monitored by thin‐layer chromatography (TLC) using E. Merck silica gel F254 TLC plates. Purification of synthesized compound was performed with flash column chromatography using Merck silica gel 60 (230–400 mesh). The ^1^H and ^13^C NMR of all the synthesized compounds was obtained using JEOL 400 and 600 spectrometer. LC–MS‐related data were obtained from HPLC (Agilent; 1260 series) with diode array detector and single quadrupole mass (Agilent; 6100 series). All compounds are >76.945% pure by HPLC analysis. Detailed synthetic procedures and characterization data for the synthesized compounds are provided in the Additional Information.

### 4.2 Cell Culture

Human CRC cell lines (CSC221, CaCo2, DLD1, HCT116, HT29, SW6200), immortalized human keratinocyte line (HaCaT), human embryonic kidney 293T (HEK293T), murine colorectal carcinoma cell line (CT26), mouse fibroblast cell line (NIH3T3) and human epithelial cell line (Beas‐2B), RV (prostate cancer), AGS (gastric), MCF‐7 (hormone sensitive breast cancer cell line), MDA‐MB‐231 (triple‐negative breast cancer), U87 (glioma), H1975 (lung cancer), and MCF10A (human mammary epithelial cell line) were cultivated in Dulbecco's modified Eagle's medium (DMEM) or Roswell Park Memorial Institute medium supplemented with 10% fetal bovine serum (FBS), 50 units/mL penicillin, and 50 mg/mL streptomycin. Cells were cultivated at 37°C in a water‐saturated environment with. 5% CO_2_. Harboring different genetic mutations information was given in Table S1 for CRC cell line.

### 4.3 Cell Viability

Cells (3 × 10^3^ cells/well) were seeded on a 96‐well plate, grown overnight, and then treated with compounds at indicated concentrations for 48 h. After incubation with MTT for 4 h at 37°C, cells were lysed with 150 µL of DMSO (Sigma–Aldrich, St. Louis, USA). Absorbance was measured at 570 nm on a microplate reader with the Gen5 software (v.2.03.1; BioTek, Winooski, VT, USA).

### 4.4 Spheroid Formation Assay

Monolayer cells were separated into single‐cell suspensions at around 70% confluence using a solution of trypsin. After that, the cells were added to N2‐supplemented DMEM/F12 that contained human basal fibroblast growth factor and human recombinant epidermal growth factor. Cells seeded at a density of 5 × 10^3^ cells/well in ultra‐low attachment 24‐well plates (Corning Inc., Corning, NY, USA). After 10–14 days of culture, spheres were quantitated by inverted phase contrast microscopy. The relative sphere formation ability was calculated through the IMT iSolution software (IMT iSolution Inc., Northampton, NJ, USA) measuring the pixel intensity of the sphere area randomly in each plate.

### 4.5 Pulldown Assay and Identification of Target Protein

DF‐L1 and DF‐L8 beads were incubated with CSC221 cell lysates in pulldown buffer (50 mM HEPES, 30 mM NaCl, 1 mM EDTA, 2.5 mM EGTA, 0.1% Tween‐20, cocktail inhibitor, pH 7.5) at 4°C for 12 h. Beads were washed thoroughly using wash buffer (pulldown buffer), and bead‐bound proteins were resolved on SDS‐PAGE. Coomassie brilliant blue G 250 or silver staining was performed for the visualization of proteins separated by SDS‐PAGE. Protein bands visualized in gel stained with Coomassie brilliant blue G 250 were removed by punching out for in‐gel digestion followed by matrix‐assisted laser desorption/ionization time‐of‐flight mass spectrometry (MALDI–TOF MS). All digested peptides were verified by PMF analysis (Genomine, Korea). After that, identified candidate targets were confirmed by immunoblot analysis with antibodies.

### 4.6 Immunoblot Analysis

The cells were lysed in a buffer (50 mmol/L Tris–HCl, pH 7.4, containing 150 mmol/L NaCl, 1% Nonidet P‐40, 0.1% deoxycholic acid, 0.1% sodium dodecyl sulfate, 10 mmol/L NaF, 0.4 mmol/L Na_3_VO_4_, 10 mmol/L Na_4_P_2_O_7_, and protease inhibitors) for 15 min on ice. The total protein content of the cells was determined by the bicinchoninic acid method. Proteins were separated by gel electrophoresis. Proteins were transferred onto a blotting membrane at a constant current of 0.20 A for 6 h using a transfer buffer containing 14.425 g/L glycine, 3.025 g/L Tris base, and 20% methanol. The membranes were incubated for 1 h at room temperature in a blocking buffer (5% of skim milk). After that probed with antibodies. Antibody information is given in Table S2. Secondary antibodies conjugated with peroxidase were used to develop the blots. Reacted proteins were visualized using Amersham ImageQuantTM 800 Western Blot Imaging System.

### 4.7 Metabolic Flux Assays

Seahorse XF96 extracellular flux analyzer (Agilent, Santa Clara, CA, USA) was used for glycolytic rate and cell mito stress test assays. Cells were seeded at 1 × 10^4^ cells/well, incubated overnight in culture medium (2% FBS), and then treated with DMSO or compound for 48 h. The plated cells were twice washed and loaded (180 µL) with assay media enhanced with glutamine, sodium pyruvate, and glucose on the day of analysis. Before analysis, the cells were incubated for 1 h at 37°C in a non‐CO_2_ incubator. Then, cell mito stress, mito tox, or glycolytic rate assay was tested. Agilent Seahorse XF Mito Stress Test Kit was utilized in accordance with Agilent Seahorse's standard methodology. This test examines the OCR of cells directly, which provides important information on mitochondrial function. First, 1 µM oligomycin (inhibits ATP synthase) was injected. The second injection was FCCP (1 µM), an uncoupling agent that compresses the proton gradient and disturbs the mitochondrial membrane potential. Last injection with 0.5 µM Rotenone (Rot; a complex I inhibitor) + antimycin A (AA; a complex III inhibitor) was loaded for OCR analysis. Agilent Seahorse XF Glycolytic Rate Assay was utilized in accordance with Agilent Seahorse's standard methodology. The assay employs a sequential injection 0.5 µM Rot + AA and 50 mM 2‐deoxy‐d‐glucose (2‐DG; a glucose analog, that inhibits glycolysis through competitive binding to glucose hexokinase). The results were analyzed using the Wave software (Agilent).

Mito Tox assay was performed according to the instructions protocol. Cells were seeded and incubated overnight in culture medium (2% FBS). After that, culture media was replaced with assay media, and cells were treated with the DMSO, Rot/AA, or indicated concentrations of compound for 2 h. The assay employs a sequential injection 1.5 µM oligomycin and 1 µM FCCP. After the data were exported from Wave Pro software, it was analyzed in Agilent XF Seahorse Analytics, a web‐based software platform.

### 4.8 Apoptosis and Cell Cycle Analysis Using Flow Cytometry

Cells were cultured in a six‐well plate at the density of 2 × 10^5^ cells/well until adherence. Cells were treated with DMSO or compound for 48 h. Apoptosis or cell cycle experiments were then performed. For apoptosis analysis, cells were harvested and washed with PBS, resuspended in 100 µL of 1× binding buffer followed by staining with 4 µL of Annexin V–FITC (BD, Biosciences, San Jose, CA, USA), and 8 µL of 50 µg/mL propidium iodide (PI; BD Biosciences, San Jose, CA, USA) for 30 min in the dark. For cell cycle analysis, cells were washed with FACS washing buffer, incubated with trypsin solution, followed by RNase A for 10 min at room temperature. Cells were centrifuged and pellets were collected and stained with 100 µL of 4 mg/mL PI (Sigma–Aldrich) for 2 h in the dark at 4°C. Cells were detected by flow cytometry on a CytoFLEX instrument (Beckman Coulter Life Sciences, Indianapolis, IN, USA).

### 4.9 CT26–iRFP Derived Tumor Growth Model

The antitumor efficacy of VDAC1/PHB/MMP‐binding compound was evaluated using a xenograft model. Six‐week‐old male BALB/C mice were obtained from OrientBio. 1 × 10⁶ CT26 cells were subcutaneously implanted to each mouse. After 1 week inoculation, mice were treated with intraperitoneal injections of either vehicle (DMSO/PBS) or SB4 at a dose of 5, 10 and 20 mg/kg, administered every other day. Tumor volumes and body weight was measured every 2 days. Bioimaging of near‐infrared fluorescence was obtained using a FOBI system (Cellgentek, Osong, Korea) for three time point. After euthanizing the mice, tumor tissues were successfully collected, and the tumor weight was measured for each sample. According to the Guiding Principles in the Care and Use of Animals (DHEW publication, NIH 80–23), the in vivo experiments were performed with the approval of the Sunchon National University Research Institutional Animal Care and Use Committee (SCNU IACUC‐2024‐17).

### 4.10 Measurement of TU, DA, SB4, and SB5 in Tissues and Plasma Using LC–MS/MS

Concentrations of TU, DA, SB4, and SB5 in tissues (liver, intestine, and tumor) and plasma were measured using LC–MS/MS. Samples were prepared by 40 µL of plasma or the supernatant separated from the tissue pellet and added 120 µL of acetonitrile (ACN) containing internal standard (IS). The samples were vortexed, sonicated for 30 min, and then left on ice for 15 min. Subsequently, the samples were centrifuged at 3000×*g* for 20 min, and the supernatant was analyzed. Calibration curves were prepared by matrix matched with tissue supernatants‐treated DMSO. The standard concentrations were set at 0, 62.5, 250, and 1000 nM for each compound. The LC–MS/MS system comprised a 40D‐XS UHPLC system (Shimadzu), and API 4000 mass spectrometer equipped with a Turbo V IonSpray source (Applied Biosystems). The sample separation was performed on a Waters Atlantis dC18 column (2.1 × 100 mm, 3 µm) using a mobile phase consisting of 0.1% formic acid in water (mobile phase A) and 0.1% formic acid in ACN (mobile phase B). A linear gradient of the two solvents was used (0 min, 0% B; 4 min, 50% B; 7 min, 95% B; 8 min, 0% B), and the equilibration time was 2 min. The flow rate was set at 0.4 mL/min, the column temperature was maintained at 30°C, and the injection volume was 10 µL.

MS/MS analysis was conducted in multiple reaction monitoring (MRM). In positive ion mode transitions were set for DA (m/z 375 → 193), SB4 (m/z 447 → 193), SB5 (m/z 461 → 193), and the IS carbamazepine (100 nM, m/z 237 → 197). In negative ion mode, MRM transitions were monitored for TU (m/z 339 → 217) and the IS 4‐methylumbelliferone (300 nM, m/z 175 → 133). The Turbo V Ion Spray interface was operated in 5500 V (pos) or −4500 V (neg), with a source temperature of 600°C. Nebulizing gas flow, auxiliary gas flow, and curtain gas flow were set at 50, 40, and 30 L/min, respectively, while the collision gas pressure was maintained at 3.4 × 10^−^⁵ Torr. Data were analyzed by analyst 1.6.2 (AB Sciex, Framingham, USA).

### 4.11 Pharmacokinetic Study

#### 4.11.1 Preparation Stock and Standard Solution, Quality Control Solution

The stock solution of SB4 and SB5 was prepared at a concentration of 1.0 mg/mL in DMSO. Additionally, the standard solutions for SB4 and SB5 were prepared by diluting with 60% ACN (0.1% formic acid) in distilled water (DW). The final concentrations of the prepared standard solutions were 5, 10, 25, 50, 100, 250, 500, and 1000 ng/mL, and they were prepared in 1.5 mL polyethylene microtubes (Axygen Inc., Union City, CA, USA). Furthermore, quality control samples were manufactured using DMSO, with final concentrations of 15 ng/mL (low quality control), 80 ng/mL (mid quality control), and 800 ng/mL (high quality control) using 60% ACN (0.1% formic acid) in DW. The IS solution (Deucravacitinib) was also prepared in DMSO and diluted to a final concentration of 10 ng/mL in 60% ACN (0.1% formic acid) in DW.

#### 4.11.2 Sample Preparation

The sample stored at −80°C was dissolved at room temperature, vortexed for 3 s, and a protein precipitation method using ACN was employed. In a 1.5 mL polyethylene microtube, 10 µL of the IS (10 ng/mL) was added to 45 µL of plasma, and 500 µL of ACN was added. Next, the sample was mixed in a vortex (M37610‐33; Barnstead International, USA) for 3 min. Then, 500 µL of the supernatant was centrifuged at 10000 g for 10 min and transferred to a LC vial, from which 10 µL was injected into the LC–MS/MS system.

#### 4.11.3 Apparatus and Chromatographic Conditions

The LC–MS/MS system comprises a SCIEX Triple Quad 6500+ system (Melbourne, Australia) coupled with a Shimadzu Nexera series SCL‐40 system controller, DGU‐405 degassing unit, LC‐40D X3 solvent delivery module, SIL‐40C X3 autosampler, and CTO‐40C column oven (Melbourne, Australia).

Chromatographic separations were performed using a Synergi 4 µm Hydro‐RP 80 Å, LC Column (30 × 2 mm). The mobile phase consisted of 0.1% FA in DW and ACN in 0.1% FA. The optimal flow rate was 0.5 mL/min, and the linear gradient elution profile was as follows: 0–0.5 min 20% B, 0.5–1.0 min 80% B, 1.0–2.0 min 80% B, 2.0–2.5 min 20% B, 2.5–3.0 min 20% B. The injection volume was 5 µL, and the total analysis time per sample was 3.0 min. The column oven temperature was maintained at 40°C.

The sample was ionized by an electrospray ionization source. The mass spectrometer was operated in positive ion mode with MRM scan mode for high sensitivity and selectivity. The turbo ion gas temperature was set at 400°C. To optimize the source parameters and compound parameters, a standard solution of 10 ng/mL was injected into the mass spectrometer at a rate of 7 µL/min through an infusion pump. The MRM transitions selected for quantification were m/z 375.075 to 150.100 for DA, m/z 447.113 to 415.200 for SB‐04, m/z 461.119 to 429.100 for SB‐05, and m/z 426.093 to 358.000 for the IS (Deucravacitinib). The system operating programs were Analyst software (version 1.7.2) and Chromelon.

#### 4.11.4 Pharmacokinetic Study in Rat

The animal testing procedure was approved by the Animal Management Committee of Dankook University (21‐038). Seven‐week‐old male Sprague–Dawley rats (weighing 250–290 g) were purchased from Samtaco Inc. (Osan, South Korea). All rats were housed randomly in groups of four, maintained under appropriate lighting and room conditions (22 ± 2°C, relative humidity 50 ± 10%, with a 12‐h light/dark cycle). Prior to the experiments, the rats had free access to food (Samtaco Inc.) and water in their cages. The animal experiments were conducted after a minimum environmental adaptation period of 1 week. On the day of the experiments, the jugular vein of each rat was cannulated individually with polyethylene tubing (Clay Adams, Parsippany, NJ, USA) under anesthesia. The cannula was exteriorized to the dorsal side of the neck, where the cannula terminated with a long silastic tubing (Dow Corning, Midland, MI, USA). The silastic tube was covered with a wire to allow free movement of the rat. Each rat was housed individually in a rat metabolic cage (Daejong Scientific Company, Seoul, Korea) and was allowed to recover from anesthesia for 4–5 h before the study began. The rats were not restrained during the experimental period. Heparinized 0.9% NaCl injectable solution (10 U/mL), 0.3 mL, was used to flush the cannula to prevent blood clotting. SB4 and SB5 were dissolved in a solution of 10% DMSO, 40% PEG 400, and 50% saline, and administered intraperitoneally or orally at a dose of 10 mg/kg (*n* = 4 for each route). Blood samples of approximately 0.25 mL were collected through the jugular vein at 0, 15, 30, 60, 120, 240, 360, and 480 min after both oral and intraperitoneal administration. The blood samples were immediately centrifuged to obtain the supernatant, which was stored at −80°C for LC–MS/MS analysis.

### 4.12 Statistics Analysis

Experimental differences were tested for statistical significance by using Sigma Plot 12.5 software (RRID:SCR_003210; Systat Software, Erkrath, Germany) followed Student's *t*‐test. *p* values of less than 0.05 were assigned to statistical significance.

## Author Contributions

M.V. and H.K. conceived and designed the study. M.V. performed comprehensive experimental work involving cellular, animal, computational. Y.H.Y., S.R.B., and H.‐H.H. prepared the chemicals. J.Y. and S.K.K. analyzed the drug distribution. Y.G.K. investigated pharmacokinetic of compounds. M.V. and H.K. analyzed the data and wrote the manuscript. All authors have read and approved the final submitted manuscript.

## Ethics Statement

Animal handling was conducted by ethical guidelines approved, adhering to relevant regulations (SCNU IACUC‐2024‐17).

## Conflicts of Interest

The authors declare no conflicts of interest.

## Supporting information




**Scheme 1**. Synthetic procedure of monomer for atraric acid analogs. (i) K_2_CO_3_, iodomethane, DMF, 50°C, 10 h; (ii) K_2_CO_3_, Benzyl bromide, DMF, 50°C, 10 h; (iii) KOH, DMSO, H_2_O, 95°C, 5 h; (iv) Cs_2_CO_3_, alkyl halide, DMF, 60°C, 8 h.
**Scheme 2**. Synthetic scheme of SB01‐SB05.
**Scheme 3**. Synthetic scheme of SB06.
**Scheme 4**. Synthetic scheme of SB07.
**Scheme 5**. Synthetic scheme of SB08.
**Scheme 6**. Synthetic scheme of SB09 & SB10.
**Scheme 7**. Synthetic scheme of SB11.
**Scheme 8**. Synthetic scheme of SB12.
**Scheme 9**. Synthetic scheme of SB13.
**Scheme 10**. Synthetic scheme of SB14.
**Scheme 11**. Synthetic scheme of SB15.
**Scheme 12**. Structure of linker‐conjugated compounds.
**Scheme 13**. Synthetic scheme of DF‐L analogs.
**Scheme 14**. Synthetic scheme of DF‐L‐01 & 08 immobilized with affigel‐10; (i) tert‐butyl (2‐bromoethyl)carbamate (DF‐L‐01 amine), tert‐butyl (3‐bromopropyl)carbamate (DF‐L‐08 amine), K_2_CO_3_, DMF, 50°C, 10 h. (ii) TFA, DCM, rt, 12 h. (iii) Affigel‐10, DMSO, rt, 4 h.
**Supplementary Figure S1**. Apoptosis test of SB compounds on CaCo2. Apoptotic cell populations using a CytoFLEX Flow Cytometer, cells were stained with Annexin V–FITC/PI. Quantification of the percentage of total apoptotic cells treated with the indicated compounds at 10 µM concentration for 48 h. Data are presented as the mean ± standard deviation, *n* = 3. *p < 0.05; **p < 0.01; ***p < 0.001; NS, no significant difference between compared DMSO.
**Supplementary Figure S2**. Cytotoxic screening in cancerous and noncancerous cell lines. CSC221, CaCo2, DLD1, HT29, HCT116, SW620, BEAS‐2B, HEK293T, HaCaT, MCF10A CT26, and NIH3T3 cells were treated with compounds for 48 h, and cell viability was measured by MTT assay. Data are presented as the mean ± standard deviation, *n* = 3.
**Supplementary Figure S3**. Synthesis of linker‐conjugated compounds (DF‐L1–DF‐L8) effect on CRC stemness. Cells were exposed to the compound at the indicated concentrations (10 µM) for 14 days. The histogram represents spheroid formation, calculated as rate relative to vehicle‐treated control, and represented as bar graphs. Data are presented as the mean ± standard deviation, *p < 0.05; **p < 0.01; ***p < 0.001; NS, no significant difference between compared DMSO.
**Supplementary Figure S4**. Characterization of the effects of the linker compound DF‐L1 on CRC stem cell inhibition. CSC221 cells were treated for 48 h with DF‐L1 (10 µM). (A–C) Protein level and quantitative analysis of mRNA encoding cancer stem markers aldehyde dehydrogenase‐1 (ALDH1), cluster of differentiation 133 (CD133), CD44, Lgr5, Musashi‐1. (D) mRNA level of *β‐catenin, c‐Myc, Cyclin‐D1*, and *EphrB‐1*. (E) *Hes‐1* mRNA levels in CSC221 cells treated with DF‐L1 (10 µM). (F‐G) Western blot analysis of Gli1, Gli2, SMO, and Bmi‐1 protein levels in CSC221 cells treated with DF‐L1 (10 µM) and incubated for 48 h. Quantitative analysis of protein expression were given in the figure. Data are presented as the mean ± standard deviation. *p < 0.05; **p < 0.01; ***p < 0.001; NS, no significant difference between compared groups.
**Supplementary Figure S5**. The identification of the target protein SB compounds by using DF‐L‐01 immobilized Affigel‐10. Indicated protein spots in the Figure 2 (DF‐L1 panel) were excised to identify the proteins using peptide mass fingerprinting and subjected MALDI TOF analysis. Mass spectra data are given in the figure.
**Supplementary Figure S6**. The identification of the target protein SB compounds by using DF‐L‐08 immobilized Affigel‐10. Indicated protein spots in the Figure 2 (DF‐L8 panel) were excised to identify the proteins using peptide mass fingerprinting and subjected MALDI TOF analysis. Mass spectra data are given in the figure.
**Supplementary Figure S7**. Revealing the clinical significance of targets. (A) Comparison of the expressions of *PHB1*, *PHB2*, *VDAC1*, *MMP9* and *FAM20B* in tumor tissues and normal tissues according to CRC subtypes using the GEPIA web tool. (B) Expression of *PHB1*, *PHB2*, *VDAC1*, *MMP9* and *FAM20B* in different clinical stages of CRC patients. (C) GEPIA web tool was searched for the disease‐free survival of high vs low levels of SB's compounds target gene.
**Supplementary Figure S8**. *PHB1*, *PHB2*, *VDAC1*, *MMP9*, and *FAM20B* expression level in CRC cell lines.
**Supplementary Figure S9**. (A) VDAC1 and MMP9 immunoblots are shown. Expression of each target protein after cells were exposed SB4 and SB5 for 48 h. (B) Cells were exposed to the compound at 2.5 and 5 µM concentration for 10–14 days. The histogram represents spheroid formation, calculated as rate relative to vehicle‐treated control, and represented as bar graphs. Data are presented as the mean ± standard deviation. *p < 0.05; **p < 0.01; ***p < 0.001; NS, no significant difference between compared groups.
**Supplementary Figure S10**. Comparison of the effects of novel VDAC1/PHB/MMP9 binding compounds and known MMP inhibitor on spheroid formation. Cells were exposed to the compound at 5 µM concentration of SB4 or SB5 and 10 µM of MMP inhibitor (GM 6001, ilomastat) for 10–14 days. The histogram represents spheroid formation, calculated as rate relative to vehicle‐treated control, and represented as bar graphs. *p < 0.05; **p < 0.01; ***p < 0.001; NS, no significant difference between compared groups.
**Supplementary Figure S11**. Using flow cytometry, the cell‐cycle distribution was evaluated on RV, AGS, MCF‐7, MDA‐MB‐231, U87, H1975, and BEAS‐2B. Cells treated with SB4 or SB5 (10 µM) for 48 h was evaluated.
**Supplementary Figure S12**. VDAC1/PHB/MMP9‐binding compounds counteracts cetuximab‐induced spheroid formation. Representative images of spheroid formation by CaCo2 cells treated with cetuximab (50 µg/mL) and SB4 (5 µM) or SB5 (5 µM) for 2 weeks, and quantitative analysis of the number of spheroids formed following each treatment. Data are presented as the mean ± standard deviation, *n* = 4. The asterisk indicates a significant difference between treatment groups, *p < 0.05; **p < 0.01; ***p < 0.001; NS, not significant.
**Supplementary Figure S13**. SB4 synergistically modulates the cell cycle. Using flow cytometry, the cell cycle distribution of CaCo2 cells treated with cetuximab, SB4 at the indicated combinations for 48 h was assessed. Quantitative measurements of sub‐G1 and G2/M populations are given in the graph. Data are presented as the mean ± standard deviation, *n* = 3. The asterisk indicates a significant difference between treatment groups, *p < 0.05; **p < 0.01; ***p < 0.001; NS, not significant.
**Supplementary Figure S14**. Determination of the activity of SB4 and SB5 on murine colorectal cancer cells on several biomarker. CT26 cells were treated with SB4 and SB5 for 48 h. After that, ALDH1, β‐catenin, c‐Myc, and PHB1 protein levels were analyzed by immunoblot assay.
**Supplementary Table S1**. Mutations of the cell lines used in the study.
**Supplementary Table S2**. Antibody information.
**Supplementary Table S3**. Primer (forward/reverse) sequences.

## Data Availability

All the data generated or analyzed during this study are included in this published article and its supplementary files.
