# Supplementary material for: Synthesized Depside Molecules Suppress the Progression of Colorectal Cancer by Binding VDAC1/PHB/MMP9 Being at the Crossroads of Stemness, Motility, Apoptosis, and Metabolism
Source: MedComm (2020). 2025 Oct 31;6(11):e70446. doi: 10.1002/mco2.70446 (PMC12579162; doi:10.1002/mco2.70446)
Supplement: Supplementary file 1 — Scheme 1. Synthetic procedure of monomer for atraric acid analogs. (i) K2CO3, iodomethane, DMF, 50°C, 10 h; (ii) K2CO3, Benzyl bromide, DMF, 50°C, 10 h; (iii) KOH, DMSO, H2O, 95°C, 5 h; (iv) Cs2CO3, alkyl halide, DMF, 60°C, 8 h. Scheme 2. Synthetic scheme of SB01‐SB05. Scheme 3. Synthetic scheme of SB06. Scheme 4. Synthetic scheme of SB07. Scheme 5. Synthetic scheme of SB08. Scheme 6. Synthetic scheme of SB09 & SB10. Scheme 7. Synthetic scheme of SB11. Scheme 8. Synthetic scheme of SB12. Scheme 9. Synthetic scheme of SB13. Scheme 10. Synthetic scheme of SB14. Scheme 11. Synthetic scheme of SB15. Scheme 12. Structure of linker‐conjugated compounds. Scheme 13. Synthetic scheme of DF‐L analogs. Scheme 14. Synthetic scheme of DF‐L‐01 & 08 immobilized with affigel‐10; (i) tert‐butyl (2‐bromoethyl)carbamate (DF‐L‐01 amine), tert‐butyl (3‐bromopropyl)carbamate (DF‐L‐08 amine), K2CO3, DMF, 50°C, 10 h. (ii) TFA, DCM, rt, 12 h. (iii) Affigel‐10, DMSO, rt, 4 h. Supplementary Figure S1. Apoptosis test of SB compounds on CaCo2. Apoptotic cell populations using a CytoFLEX Flow Cytometer, cells were stained with Annexin V–FITC/PI. Quantification of the percentage of total apoptotic cells treated with the indicated compounds at 10 µM concentration for 48 h. Data are presented as the mean ± standard deviation, n = 3. *p < 0.05; **p < 0.01; ***p < 0.001; NS, no significant difference between compared DMSO. Supplementary Figure S2. Cytotoxic screening in cancerous and noncancerous cell lines. CSC221, CaCo2, DLD1, HT29, HCT116, SW620, BEAS‐2B, HEK293T, HaCaT, MCF10A CT26, and NIH3T3 cells were treated with compounds for 48 h, and cell viability was measured by MTT assay. Data are presented as the mean ± standard deviation, n = 3. Supplementary Figure S3. Synthesis of linker‐conjugated compounds (DF‐L1–DF‐L8) effect on CRC stemness. Cells were exposed to the compound at the indicated concentrations (10 µM) for 14 days. The histogram represents spheroid formation, calculated as rate rela [file MCO2-6-e70446-s001.docx]

**Synthesized depside molecules suppress the progression of colorectal cancer by binding VDAC1/PHB/MMP9 being at the crossroads of stemness, motility, apoptosis, and metabolism**

Mücahit Varlı ^1^, Young Hyun Yu ^1^, Jieun Yu ^2^, Suresh R. Bhosle ^1^, Sang Kyum Kim ^2^, Yoon Gyoon Kim ^3^, Hyung-Ho Ha ^1,*^, Hangun Kim ^1,*^

^1^College of Pharmacy, Sunchon National University, 255 Jungang-ro, Sunchon, Jeonnam, Korea; mucahitvarli@s.scnu.ac.kr (M.V.); chmyyh@gmail.com (Y.H.Y.); bhoslesuresh1005@gmail.com (S.R.B.); hhha@sunchon.ac.kr (H.-H.H.)

^2^College of Pharmacy, Chungnam National University, Daejeon, Korea; rhdwn0117@hanmail.net (J.Y.); sangkim@cnu.ac.kr (S.K.K.)

^3^College of Pharmacy, Dankook University, 119 Dandaero, Dongnam-gu, Cheonan-si, Korea; nicebw@gmail.com (Y.G.K.)

* Correspondence: hangunkim@sunchon.ac.kr; Tel.: +82-61-750-3761, hhha@sunchon.ac.kr; Tel.: +82-61-750-3754

**Additional Materials and Method**

**1. Synthesis of SB compounds**

**1.1. Preparation of Monomer** **for Atraric acid analogs**


**Scheme 1.**Synthetic Procedure of monomer for Atraric acid analogs. (i) K_2_CO_3_, Iodomethane, DMF, 50^o^C,10h; (ii) K_2_CO_3_, Benzyl bromide, DMF, 50^o^C,10h; (iii) KOH, DMSO, H_2_O, 95^o^C,5h; (iv) Cs_2_CO_3_, Alkyl halide, DMF, 60^o^C,8h

**1.1.1. General Procedure of A-M Monomer Compounds for Atraric acid analogs**

(i) Methylation procedure ; To a solution of methyl 2,4-dihydroxy-3,6-dimethylbenzoate (1eq.) in DMF were added K_2_CO_3_ (4eq.) and methyl iodide (4eq.) at the room temperature under an argon atmosphere. After being stirred at 50^o^C for 10 h, the reaction mixture was filtered through a pad of celite. The filtrate was concentrated in a vacuum, and the resulting residue was purified by flash column chromatography on silica gel.

(ii) Benzylation procedure ; To a solution of methyl 2,4-dihydroxy-3,6-dimethylbenzoate (1eq.) in DMF were added K_2_CO_3_ (4eq.) and benzylbromide (4eq.) at the room temperature under an argon atmosphere. After being stirred at 50^o^C for 10 h, the reaction mixture was filtered through a pad of celite. The filtrate was concentrated in a vacuum, and the resulting residue was purified by flash column chromatography on silica gel. In case of mono-benzylation, using K_2_CO_3_ (2eq.) and benzylbromide (1eq.).

(iii) Hydrolysis procedure ; To a solution of methyl ester (1eq.) and KOH (5eq.) in DMSO(4 mL) and H_2_O(1 mL) was stirred at 95^o^C for 5 h. Then the solution was cool at room temperature, acidified with 1 N HCl and extracted with ethylacetate. The organic phase was washed with brine and dried over anhydrous sodium sulfate. The filtrate was concentrated in a vacuum, and the resulting residue was purified by flash column chromatography on silica gel.

(iv) Alkylation procedure ; To a solution of methyl 4-(benzyloxy)-2-hydroxy-3,6-dimethylbenzoate (1eq.) in DMF were added Cs_2_CO_3_ (2eq.) and Alkyl halide (2eq.) at the room temperature under an argon atmosphere. After being stirred at 60^o^C for 8 h, the reaction mixture was filtered through a pad of celite. The filtrate was concentrated in a vacuum, and the resulting residue was purified by flash column chromatography on silica gel.

**1.1.2. Monomer B. Methyl 2,4-dimethoxy-3,6-dimethylbenzoate**

^1^H-NMR (400 MHz, CDCl3-d) δ 6.44 (s, 1H), 3.88 (s, 3H), 3.79 (s, 3H), 3.73 (s, 3H), 2.28 (s, 3H), 2.08 (s, 3H). ^13^C-NMR (151 MHz, CDCl3-d): δ 169.01, 159.49, 156.75, 134.77, 120.81, 117.25, 107.91, 61.87, 55.67, 52.03, 19.86, 8.72, 1.08. MS (ESI+, m/z): 225 [M+H]

**1.1.3. Monomer C. 2,4-Dimethoxy-3,6-dimethylbenzoic acid**

^1^H-NMR (400 MHz, CDCl3-d) δ 6.55-6.70 (m, 1H), 3.91 (d, J = 70.6 Hz, 3H), 3.83 (s, 3H), 2.41-2.88 (m, 3H), 2.02-2.30 (m, 3H).^13^C-NMR (151 MHz, CDCl3-d): δ 160.65, 158.12, 140.56, 117.39, 109.96, 62.48, 55.77, 22.35, 8.86. MS (ESI+, m/z): 211 [M+H]

**1.1.4. Monomer D. Methyl 2,4-bis(benzyloxy)-3,6-dimethylbenzoate**

^1^H-NMR (400 MHz, CDCl3-d) δ 7.48 (m, 4H), 7.41-7.44 (m, 4H), 7.37 (q, J = 1.7 Hz, 2H), 6.61 (s, 1H), 5.10 (s, 2H), 4.94 (s, 2H), 3.83 (s, 3H), 2.35 (s, 3H), 2.25 (s, 3H). ^13^C-NMR (151 MHz, CDCl3-d): δ 169.18, 158.70, 155.63, 141.15, 137.46, 137.10, 134.98, 128.71, 128.61, 128.59, 128.13, 128.05, 127.62, 127.28, 127.08, 121.56, 118.22, 109.64, 76.40, 70.32, 65.23, 52.16, 20.00, 9.41. MS (ESI+, m/z): 377 [M+H]

**1.1.5. Monomer E. 2,4-Bis(benzyloxy)-3,6-dimethylbenzoic acid**

^1^H-NMR (400 MHz, CDCl3-d) δ 7.49-7.34 (10H), 6.67-6.63 (1H), 5.26-5.06 (2H), 5.06-4.83 (2H), 4.83-4.60 (1H), 2.62-2.41 (3H), 2.41-2.15 (3H). ^13^C-NMR (151 MHz, CDCl3-d): δ 169.05, 159.62, 156.57, 140.88, 139.37, 136.67, 135.93, 128.78, 128.73, 128.63, 128.17, 127.76, 127.27, 127.09, 118.26, 117.64, 111.02, 70.32, 65.46, 21.91, 9.42. MS (ESI+, m/z): 363 [M+H]

**1.1.6. Monomer F. 2,4-Dihydroxy-3,6-dimethylbenzoic acid**

^1^H-NMR (400 MHz, DMSO-d6) δ 10.01 (s, 1H), 6.21 (s, 1H), 2.35 (s, 3H), 1.88 (s, 3H). ^13^C-NMR (151 MHz, DMSO-d6): δ 174.61, 163.55, 160.45, 140.04, 110.82, 108.42, 104.08, 24.25, 8.47. MS (ESI+, m/z): 183 [M+H]

**1.1.7. Monomer G. Benzyl 2,4-dihydroxy-3,6-dimethylbenzoate**

^1^H-NMR (600 MHz, CDCl3-d) δ 12.11-11.95 (1H), 7.50-7.40 (2H), 7.40-7.36 (2H), 7.36-7.31 (1H), 6.26-6.08 (1H), 5.39-5.36 (2H), 2.48-2.39 (3H), 2.12-2.08 (3H). ^13^C-NMR (151 MHz, CDCl3-d): δ 171.99, 163.39, 158.21, 140.36, 135.57, 128.73, 128.49, 110.70, 108.69, 105.26, 67.09, 24.48, 7.73. MS (ESI+, m/z): 273 [M+H]

**1.1.8. Monomer H. Methyl 4-(benzyloxy)-2-hydroxy-3,6-dimethylbenzoate**

^1^H-NMR (600 MHz, CDCl3-d) δ 11.83 (s, 1H), 7.42 (d, J = 7.2 Hz, 2H), 7.37-7.40 (m, 2H), 7.32 (t, J = 7.2 Hz, 1H), 6.34 (s, 1H), 5.11 (s, 2H), 3.94 (d, J = 29.3 Hz, 3H), 2.50 (s, 3H), 2.14 (d, J = 8.6 Hz, 3H). ^13^C-NMR (151 MHz, CDCl3-d): δ 72.61, 162.33, 160.67, 140.13, 136.94, 128.65, 128.00, 127.14, 111.50, 107.20, 105.78, 69.94, 51.89, 24.70, 8.15. MS (ESI+, m/z): 287 [M+H]

**1.1.9. Monomer I. 4-(Benzyloxy)-2-hydroxy-3,6-dimethylbenzoic acid**

^1^H-NMR (400 MHz, DMSO-D6) δ 7.41 (d, J = 7.2 Hz, 2H), 7.35-7.38 (m, 2H), 7.29 (t, J = 7.2 Hz, 1H), 6.53 (s, 1H), 5.14 (s, 2H), 2.44 (s, 3H), 1.97 (s, 4H). ^13^C-NMR (151 MHz, DMSO-d6): δ 174.38, 162.29, 160.40, 140.64, 137.50, 129.02, 128.37, 127.85, 110.31, 107.71, 106.24, 69.82, 24.46, 8.57. MS (ESI+, m/z): 273 [M+H]

**1.1.10. Monomer J. Methyl 4-(benzyloxy)-3,6-dimethyl-2-propoxybenzoate**

^1^H-NMR (600 MHz, CDCl3-d) δ 7.42-7.46 (m, 2H), 7.35-7.40 (m, 2H), 7.30-7.35 (m, 1H), 6.52 (s, 1H), 5.03-5.09 (m, 2H), 3.85-3.93 (m, 3H), 3.74-3.85 (m, 2H), 2.24-2.31 (m, 3H), 2.13-2.19 (m, 3H), 1.71-1.80 (m, 2H), 0.98-1.05 (m, 3H). ^13^C-NMR (151 MHz, CDCl3-d): δ 169.16, 158.56, 155.92, 137.12, 134.58, 128.63, 127.96, 127.20, 121.41, 117.92, 109.15, 70.22, 52.04, 23.53, 19.86, 10.61, 9.17. MS (ESI+, m/z): 329 [M+H]

**1.1.11. Monomer K. 4-(Benzyloxy)-3,6-dimethyl-2-propoxybenzoic acid**

^1^H-NMR (600 MHz, CDCl3-d) δ 7.38-7.43 (m, 4H), 7.32-7.35 (m, 1H), 6.63 (s, 1H), 5.10 (s, 2H), 3.85-3.87 (m, 2H), 2.56 (d, J = 11.7 Hz, 3H), 2.17 (d, J = 8.6 Hz, 3H), 1.83-1.89 (m, 2H), 1.02-1.07 (m, 3H). ^13^C-NMR (151 MHz, CDCl3-d): δ 159.82, 157.28, 136.57, 128.72, 128.19, 127.26, 117.92, 111.37, 70.28, 23.29, 22.62, 10.32, 9.22. MS (ESI+, m/z): 315 [M+H]

**1.1.12. Monomer L. Methyl 4-(benzyloxy)-2-ethoxy-3,6-dimethylbenzoate**

^1^H-NMR (600 MHz, CDCl3-d) δ 7.44-7.40 (2H), 7.40-7.36 (2H), 7.34-7.30 (1H), 6.54-6.49 (1H), 5.09-5.05 (2H), 3.96-3.86 (5H), 2.30-2.26 (3H), 2.20-2.15 (3H), 1.38-1.32 (3H). ^13^C-NMR (151 MHz, CDCl3-d): δ 169.16, 158.57, 155.95, 137.10, 134.64, 128.71, 128.64, 128.60, 128.31, 127.97, 127.21, 121.41, 117.97, 109.19, 70.31, 70.23, 66.91, 52.07, 19.91, 15.68, 15.50, 9.23. MS (ESI+, m/z): 315 [M+H]

**1.1.13. Monomer M. 4-(Benzyloxy)-2-ethoxy-3,6-dimethylbenzoic acid**^1^H-NMR (600 MHz, CDCl3-d) δ 7.38-7.43 (m, 4H), 7.33-7.36 (m, 2H), 6.64 (s, 1H), 5.10 (s, 2H), 3.99 (q, J = 7.0 Hz, 2H), 2.57 (d, J = 11.4 Hz, 3H), 2.16 (d, J = 11.0 Hz, 3H), 1.44-1.47 (m, 3H). ^13^C-NMR (151 MHz, CDCl3-d): δ 159.83, 157.21, 136.55, 128.73, 128.20, 127.27, 117.96, 111.44, 71.75, 70.28, 22.69, 15.41, 9.29. MS (ESI+, m/z): 301 [M+H]

**1.2. General Procedure of SB-01 to SB-15 compounds**

(a) Esterification with TFAA(Trifluoroacetic anhydride); To a solution of substituted phenol (1eq.) and benzoic acid (1eq.) in anhydrous toluene was added TFAA (8eq.) by dropwise addition at 0°C. After completing of additions transfer the reaction mixture to rt. The mixture was stirred for 5h. After completion of the reaction, the mixture was extracted with ethyl acetate, washed with brine, and dried over anhydrous Na_2_SO_4_, concentrated under a high-pressure vacuum. The residue was purified by column chromatography.

(b) Debenzylation with H_2_,Pd/C; To a solution of in ethyl acetate, 10% palladium on carbon was added, and the suspension was stirred in a hydrogen atmosphere for 8h. Then, the solution was filtered through a pad of celite, concentrated and purified by column chromatography.

**Scheme 2.**Synthetic Scheme of SB01-SB05.

**1.2.1. Synthesis of 3-hydroxy-4-(methoxycarbonyl)-2,5-dimethylphenyl 2,4-dimethoxy-3,6-dimethylbenzoate (SB-01)**

^1^H-NMR (400 MHz, CDCl3) δ 11.90 (s, 1H), 6.58 (s, 1H), 6.54 (s, 1H), 3.97 (s, 3H), 3.85 (d, J = 6.9 Hz, 6H), 2.54 (s, 3H), 2.47 (s, 3H), 2.17 (d, J = 6.4 Hz, 6H) ^13^C-NMR (151 MHz, DMSO-D6) δ 170.33, 166.13, 160.01, 158.21, 156.91, 152.10, 137.33, 135.28, 119.90, 116.95, 116.91, 116.09, 115.50, 108.96, 62.28, 56.32, 52.83, 21.73, 19.99, 9.66, 9.26. MS (ESI+, m/z): 389 [M+H]

**1.2.2. 3-Methoxy-4-(methoxycarbonyl)-2,5-dimethylphenyl 2,4-dimethoxy-3,6-dimethylbenzoate (SB-02)**

^1^H-NMR (400 MHz, CDCl3) : δ 6.82 (s, 1H), 6.54 (s, 1H), 3.93 (s, 3H), 3.86-3.81 (m, 9H), 2.47 (s, 3H), 2.31 (s, 3H), 2.21 (s, 3H), 2.16 (s, 3H). ^13^C-NMR (101 MHz, CDCl3): δ 168.5, 166.5, 160.1, 157.2, 156.8, 151.1, 135.4, 134.6, 126.5, 122.3, 119.8, 119.4, 117.6, 108.2, 62.2, 62.1, 55.8, 52.3, 20.3, 19.3, 9.7, 9.0. MS (ESI+, m/z): 403 [M+H]

**1.2.3. 3-Ethoxy-4-(methoxycarbonyl)-2,5-dimethylphenyl 2,4-dimethoxy-3,6-dimethylbenzoate (SB-03)**

^1^H-NMR (400 MHz, CDCl3) : δ 6.81 (s, 1H), 6.53 (s, 1H), 3.97 (d, J = 7.3 Hz, 2H), 3.92 (s, 3H), 3.85 (d, J = 9.1 Hz, 6H), 2.46 (s, 3H), 2.30 (s, 3H), 2.19 (s, 3H), 2.16 (s, 3H), 1.36 (t, J = 7.1 Hz, 3H). ^13^C-NMR (151 MHz, DMSO-D6) δ 168.19, 166.27, 160.04, 156.94, 155.67, 150.89, 135.33, 134.42, 127.20, 122.43, 119.83, 119.55, 116.96, 109.02, 70.62, 62.34, 56.37, 52.78, 20.04, 18.99, 15.92, 10.02, 9.31. MS (ESI+, m/z): 417 [M+H]

**1.2.4. 4-(Methoxycarbonyl)-3-(2-methoxyethoxy)-2,5-dimethylphenyl 2,4-dimethoxy-3,6-dimethylbenzoate (SB-04)**

^1^H-NMR (400 MHz, CDCl_3_) : δ 6.81 (s, 1H), 6.52 (s, 1H), 4.05 (t, J = 4.6 Hz, 2H), 3.90 (s, 3H), 3.83 (d, J = 12.7 Hz, 6H), 3.66 (t, J = 4.6 Hz, 2H), 3.42 (s, 3H), 2.45 (s, 3H), 2.29 (s, 3H), 2.21 (s, 3H), 2.14 (s, 3H) ^13^C-NMR (151 MHz, DMSO-D_6_) δ 168.09, 166.26, 160.04, 156.95, 155.38, 150.92, 135.34, 134.50, 127.13, 122.53, 119.82, 119.70, 116.96, 109.02, 73.99, 71.42, 62.34, 58.71, 56.37, 52.77, 20.04, 18.98, 9.84, 9.30. MS (ESI+, m/z): 447 [M+H]

**1.2.5. 4-(Methoxycarbonyl)-3-(3-methoxypropoxy)-2,5-dimethylphenyl 2,4-dimethoxy-3,6-dimethylbenzoate (SB-05)**

^1^H-NMR (400 MHz, CDCl_3_) : δ 6.80 (s, 1H), 6.52 (s, 1H), 3.97 (t, J = 6.2 Hz, 2H), 3.91 (d, J = 3.1 Hz, 3H), 3.85 (s, 3H), 3.83 (d, J = 3.1 Hz, 3H), 3.55 (t, J = 6.4 Hz, 2H), 3.35 (s, 3H), 2.45 (s, 3H), 2.29 (s, 3H), 2.18 (s, 3H), 2.14 (s, 3H), 2.00 (q, J = 6.3 Hz, 2H) ^13^C-NMR (151 MHz, DMSO-D_6_) δ 168.11, 166.27, 160.04, 156.94, 155.48, 150.92, 135.34, 134.48, 127.14, 122.40, 119.81, 119.62, 116.96, 108.99, 71.82, 68.92, 62.31, 58.43, 56.33, 52.72, 30.27, 20.01, 18.95, 9.82, 9.27. MS (ESI+, m/z): 461 [M+H]

**Scheme 3.**Synthetic Scheme of SB06.

**1.2.6. Benzyl 4-((2,4-dimethoxybenzoyl)oxy)-2-hydroxy-3,6-dimethylbenzoate(GN)**

^1^H-NMR (400 MHz, CDCl_3_) : δ 11.88 (s, 1H), 8.07 (d, J = 8.6 Hz, 1H), 7.35-7.44 (m, 5H), 6.54 (d, J = 17.2 Hz, 3H), 5.40 (s, 2H), 3.90 (s, 3H), 3.88 (s, 3H), 2.49 (s, 3H), 2.11 (s, 3H). ^13^C-NMR (101 MHz, CDCl_3_): δ 171.86, 165.24, 163.14, 162.96, 162.39, 153.74, 139.43, 135.30, 134.64, 128.78, 128.60, 128.56, 117.07, 110.92, 109.42, 105.03, 99.14, 77.36, 67.45, 56.07, 55.68, 24.38, 9.17. MS (ESI+, m/z): 437 [M+H]

**1.2.7. 4-((2,4-Dimethoxybenzoyl)oxy)-2-hydroxy-3,6-dimethylbenzoic acid (SB-06)**

^1^H-NMR (400 MHz, DMSO-D_6_) : δ 7.90 (d, J = 8.6 Hz, 1H), 6.67 (d, J = 2.1 Hz, 1H), 6.63 (dd, J = 8.8, 2.2 Hz, 1H), 6.56 (s, 1H), 3.83 (s, 3H), 3.83 (s, 3H), 2.43 (s, 3H), 1.93 (s, 3H). ^13^C-NMR (151 MHz, DMSO-D_6_) : δ 173.79, 165.40, 162.94, 162.13, 161.86, 153.22, 139.37, 134.36, 116.87, 116.32, 111.40, 110.54, 106.32, 99.59, 56.53, 56.25, 23.36, 9.46. MS (ESI+, m/z): 347 [M+H]

**Scheme 4.**Synthetic Scheme of SB07.

**1.2.8. Methyl 4-((2,4-dihydroxybenzoyl)oxy)-2-hydroxy-3,6-dimethylbenzoate (SB-07)**

^1^H-NMR (600 MHz, METHANOL-D_4_) δ 7.84 (d, J = 9.0 Hz, 1H), 6.60 (s, 1H), 6.42 (dd, J = 8.6, 2.4 Hz, 1H), 6.34 (d, J = 2.4 Hz, 1H, 3.58 (s, 3H), 2.30 (s, 3H), 1.96 (s, 3H) ^13^C-NMR (151 MHz, METHANOL-D_4_) δ 167.93, 167.45, 165.18, 164.20, 158.08, 148.42, 136.71, 131.96, 117.00, 116.04, 114.19, 108.32, 103.45, 102.33, 74.74, 19.09, 7.87. MS (ESI+, m/z): 332 [M+H]

**Scheme 5.**Synthetic Scheme of SB08.

**1.2.9. Benzyl 4-((2,4-bis(benzyloxy)-3,6-dimethylbenzoyl)oxy)-2-hydroxy-3,6-dimethylbenzoate (SB-08)**

^1^H-NMR (600 MHz, DMSO-D_6_) δ 10.47 (s, 1H), 7.49-7.33 (m, 15H), 6.92 (s, 1H), 6.36 (d, J = 8.6 Hz, 1H), 5.35 (s, 2H), 5.19 (s, 2H), 4.91 (d, J = 3.8 Hz, 2H), 2.40 (d, J = 3.4 Hz, 3H), 2.16 (s, 6H), 1.91 (s, 3H). ^13^C-NMR (151 MHz, DMSO-D_6_) δ 169.50, 166.24, 159.06, 157.76, 155.51, 151.83, 137.43, 137.31, 136.82, 136.11, 135.14, 129.06, 128.92, 128.89, 128.80, 128.59, 128.44, 128.22, 127.94, 120.53, 117.84, 116.88, 116.01, 110.57, 76.33, 70.22, 67.39, 21.44, 20.07, 9.80, 9.66. MS (ESI+, m/z): 617 [M+H]

**Scheme 6.**Synthetic Scheme of SB09 & SB10.

**1.2.10. 3-Hydroxy-4-(methoxycarbonyl)-2,5-dimethylphenyl 2,4-bis(benzyloxy)-3,6-dimethylbenzoate (SB-09)**

^1^H-NMR (400 MHz, CDCl_3_) : δ 11.87 (s, 1H), 7.46-7.35 (m, 10H), 6.65 (s, 1H), 6.36 (s, 1H), 5.13 (s, 2H), 4.99 (s, 2H), 3.95 (s, 3H), 2.48 (s, 3H), 2.38 (s, 3H), 2.27 (s, 3H), 2.06 (s, 3H). ^13^C-NMR (151 MHz, DMSO-D_6_) δ 172.36, 170.19, 166.25, 162.15, 160.52, 159.06, 157.90, 155.51, 151.86, 139.28, 137.43, 137.32, 136.99, 135.14, 129.06, 128.92, 128.59, 128.43, 128.22, 127.94, 120.54, 117.84, 116.78, 116.01, 115.73, 111.05, 110.57, 108.68, 104.62, 76.33, 70.22, 52.83, 52.47, 23.96, 21.53, 20.08, 9.78, 9.66, 8.52. MS (ESI+, m/z): 541 [M+H]

**1.2.11. 3-Hydroxy-4-(methoxycarbonyl)-2,5-dimethylphenyl 2,4-dihydroxy-3,6-dimethylbenzoate (SB-10)**

^1^H-NMR (400 MHz, CDCl_3_) : δ 11.93 (s, 1H), 11.72 (s, 1H), 6.51 (s, 1H), 6.31 (s, 1H), 5.12 (s, 1H), 3.98 (s, 3H), 2.62 (s, 3H), 2.53 (s, 3H), 2.13 (s, 3H), 2.08 (s, 3H). ^13^C-NMR NMR (151 MHz, DMSO-D_6_) δ 170.26, 169.87, 162.49, 161.29, 157.90, 151.60, 139.56, 137.12, 116.64, 116.45, 115.76, 111.50, 109.11, 104.19, 52.86, 24.01, 21.59, 9.84, 8.54. MS (ESI+, m/z): 360 [M+H]

**Scheme 7.**Synthetic Scheme of SB11.

**1.2.12. Benzyl 4-((4-(benzyloxy)-2-hydroxy-3,6-dimethylbenzoyl)oxy)-2-hydroxy-3,6-dimethylbenzoate (GI)**

^1^H-NMR (400 MHz, CDCl_3_) : δ 11.92 (s, 1H), 11.53 (s, 1H), 7.36-7.44 (m, 10H), 6.50 (s, 1H), 6.45 (s, 1H), 5.43 (s, 2H), 5.17 (s, 2H), 2.67 (s, 3H), 2.52 (s, 3H), 2.18 (s, 3H), 2.09 (s, 3H). ^13^C-NMR (101 MHz, CDCl_3_): δ 171.70, 170.25, 163.26, 163.09, 161.53, 152.71, 140.74, 139.87, 136.75, 135.20, 128.81, 128.73, 128.69, 128.57, 128.12, 127.16, 117.12, 116.51, 111.95, 110.02, 107.79, 104.68, 70.08, 67.58, 25.14, 24.39, 9.42, 8.18. MS (ESI+, m/z): 527 [M+H]

**1.2.13. 4-((2,4-Dihydroxy-3,6-dimethylbenzoyl)oxy)-2-hydroxy-3,6-dimethylbenzoic acid (SB-11)**

^1^H-NMR (600 MHz, METHANOL-D_4_) : δ 6.48 (s, 1H), 6.29 (s, 1H), 2.55 (s, 3H), 2.53 (s, 3H), 2.01 (s, 3H), 2.00 (s, 3H). ^13^C-NMR (151 MHz, METHANOL-D_4_) δ 173.80, 170.29, 163.77, 162.90, 161.18, 152.41, 140.25, 140.13, 116.21, 115.93, 110.99, 110.03, 109.12, 102.71, 78.02, 77.80, 77.59, 23.63, 22.86, 8.32, 6.84. MS (ESI+, m/z): 346 [M+H]

**Scheme 8.**Synthetic Scheme of SB12.

**1.2.14. Benzyl 4-((2,4-dihydroxy-3,6-dimethylbenzoyl)oxy)-2-hydroxy-3,6-dimethylbenzoate (SB-12)**

^1^H-NMR (400 MHz, CDCl_3_) : δ 11.91 (s, 1H), 11.70 (s, 1H), 7.36-45 (d, J = 10.1 Hz, 5H), 6.50 (s, 1H), 6.30 (s, 1H), 5.43 (s, 2H), 2.61 (s, 3H), 2.52 (s, 3H), 2.13 (s, 3H), 2.08 (s, 3H). ^13^C-NMR (151 MHz, DMSO-D_6_) δ 169.86, 169.55, 162.44, 161.27, 157.72, 151.57, 139.54, 136.95, 136.13, 129.05, 128.86, 128.79, 116.73, 116.46, 116.09, 111.49, 109.12, 104.24, 67.39, 23.98, 21.51, 9.86, 8.54. MS (ESI+, m/z): 437 [M+H]

**Scheme 9.**Synthetic Scheme of SB13.

**1.2.15. 3-Hydroxy-4-(methoxycarbonyl)-2,5-dimethylphenyl 4-(benzyloxy)-3,6-dimethyl-2-propoxybenzoate (SB-13)**

^1^H-NMR (600 MHz, CDCl_3_) :δ 11.88 (s, 1H), 7.44 (d, J = 7.2 Hz, 2H), 7.38-7.41 (m, 2H), 7.34 (d, J = 7.2 Hz, 1H), 6.59 (s, 2H), 5.10 (s, 2H), 3.96 (d, J = 5.2 Hz, 3H), 3.88 (t, J = 6.9 Hz, 2H), 2.53 (d, J = 7.9 Hz, 3H), 2.44 (s, 3H), 2.20 (s, 3H), 2.15 (s, 3H), 1.81 (q, J = 7.2 Hz, 2H), 1.00 (t, J = 7.4 Hz, 3H). ^13^C-NMR NMR(151 MHz, CDCl_3_) : δ 172.44, 162.95, 159.12, 156.45, 153.37, 151.68, 139.46, 136.98, 135.07, 128.68, 128.03, 127.20, 120.47, 118.22, 117.14, 116.45, 109.74, 109.35, 70.29, 52.22, 24.13, 23.47, 20.26, 10.49, 9.32. MS (ESI+, m/z): 493 [M+H]

**Scheme 10.**Synthetic Scheme of SB14.

**1.2.16. Benzyl 4-((4-(benzyloxy)-3,6-dimethyl-2-propoxybenzoyl)oxy)-2-hydroxy-3,6-dimethylbenzoate (SB-14)**

^1^H-NMR (600 MHz, CDCl_3_) : δ 12.11-11.95 (1H), 7.50-7.40 (2H), 7.40-7.36 (2H), 7.36-7.31 (1H), 6.26-6.08 (1H), 5.39-5.36 (2H), 2.48-2.39 (3H), 2.12-2.08 (3H). ^13^CNMR(151 MHz, CDCl_3_) : δ 171.77, 166.40, 163.15, 159.15, 156.48, 153.48, 139.61, 137.00, 135.28, 135.09, 128.80, 128.69, 128.64, 128.56, 128.04, 127.22, 120.49, 118.23, 117.25, 116.54, 109.72, 109.38, 70.30, 67.49, 24.46, 23.49, 20.28, 10.51, 9.37, 9.35. MS (ESI+, m/z): 569 [M+H]

**Scheme 11.**Synthetic Scheme of SB15.

**1.2.17. Benzyl 4-((4-(benzyloxy)-2-ethoxy-3,6-dimethylbenzoyl)oxy)-2-hydroxy-3,6-dimethylbenzoate (SB-15)**

^1^H-NMR (600 MHz, CDCl_3_) δ 11.88 (s, 1H), 7.43 (dd, J = 6.9, 5.2 Hz, 4H), 7.38-7.41 (m, 4H), 7.32-7.37 (m, 2H), 6.59 (s, 1H), 6.57 (s, 1H), 5.41 (s, 2H), 5.07 (d, J = 28.9 Hz, 2H), 4.00 (q, J = 7.0 Hz, 2H), 2.52 (s, 3nH, 2.45 (d, J = 9.3 Hz, 3H), 2.20 (s, 3H), 2.16 (s, 3H), 1.36-1.41 (m, 3H). ^13^C-NMR (151 MHz, CDCl_3_) : 171.76, 166.40, 163.12, 159.13, 156.39, 153.46, 139.61, 136.96, 135.26, 135.11, 128.78, 128.68, 128.63, 128.54, 128.04, 127.21, 120.48, 118.27, 117.29, 116.49, 109.72, 109.39, 70.56, 70.29, 67.47, 29.78, 24.45, 20.23, 15.58, 9.36, 9.34, 1.09. MS (ESI+, m/z): 555 [M+H]

**1.3. HR-MS & Purity Characterization of SB compounds**

**1.3.1. SB-01 (Purity 96.069%)**: HR-MS [M]^+^: *m/z* calcd for C_21_H_25_O_7_ 388.1600, found 389.1591

**
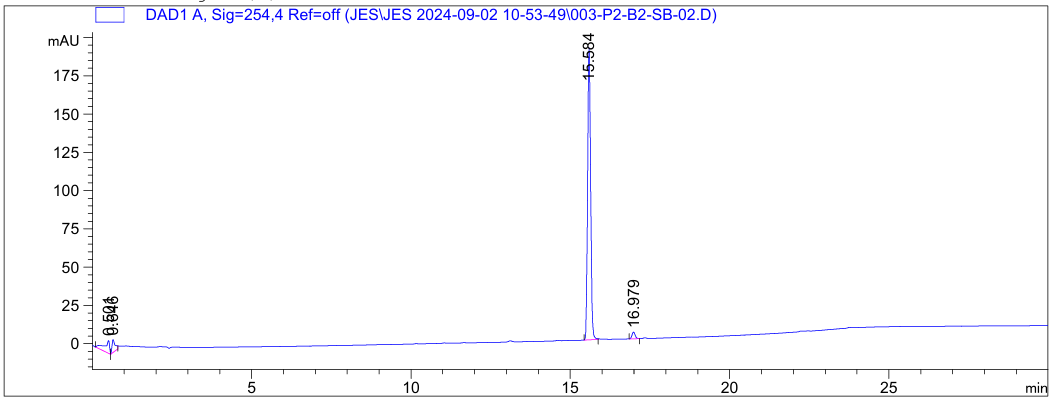
**

**
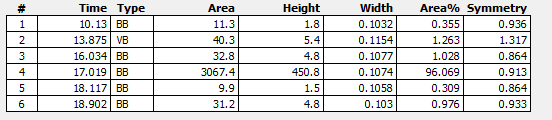
**

**1.3.2.** **SB-02 (Purity 97.653%)**: HR-MS [M]^+^: *m/z* calcd for C_22_H_27_O_7_ 403.1757, found 403.1746


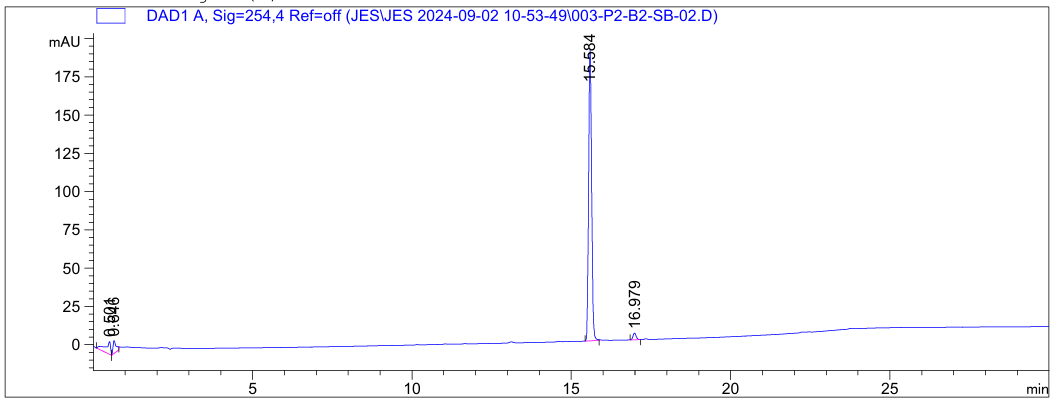


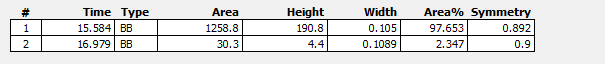


**1.3.3. SB-03 (Purity 99.99%)**: HR-MS [M]^+^: *m/z* calcd for C_23_H_29_O_7_ 417.1913, found 417.1904


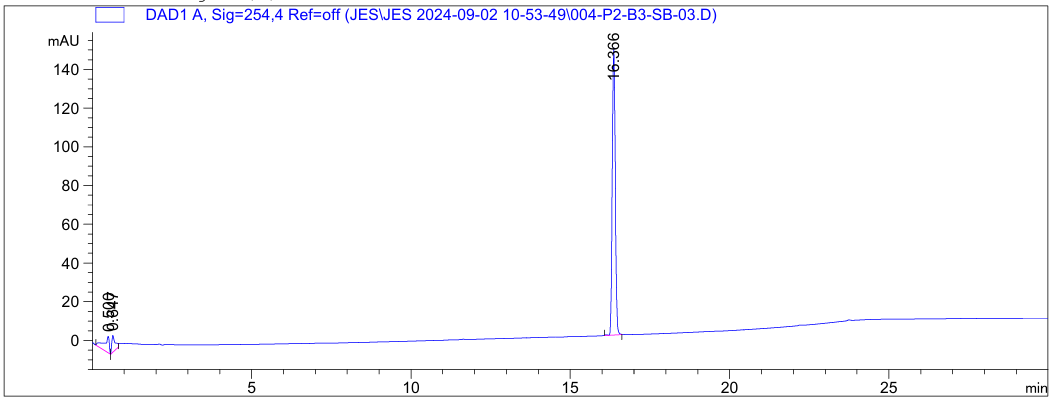


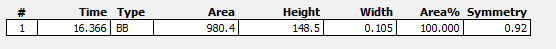


**1.3.4. SB-04 (97.625%)**: HR-MS [M]^+^: *m/z* calcd for C_24_H_31_O_8_ 447.2019, found 447.2009

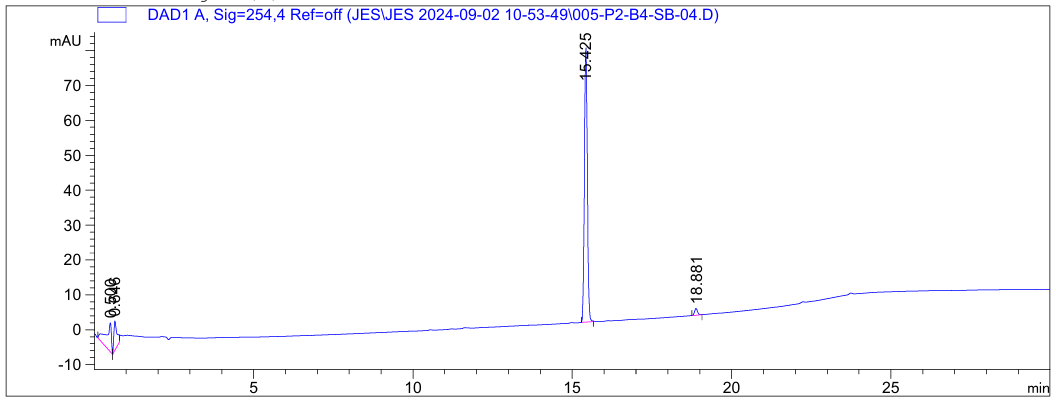


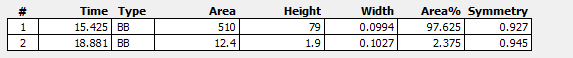


**1.3.5. SB-05 (97.558%)**: HR-MS [M]^-^: *m/z* calcd for C_25_H_31_O_8_ 459.2019, found 459.2020


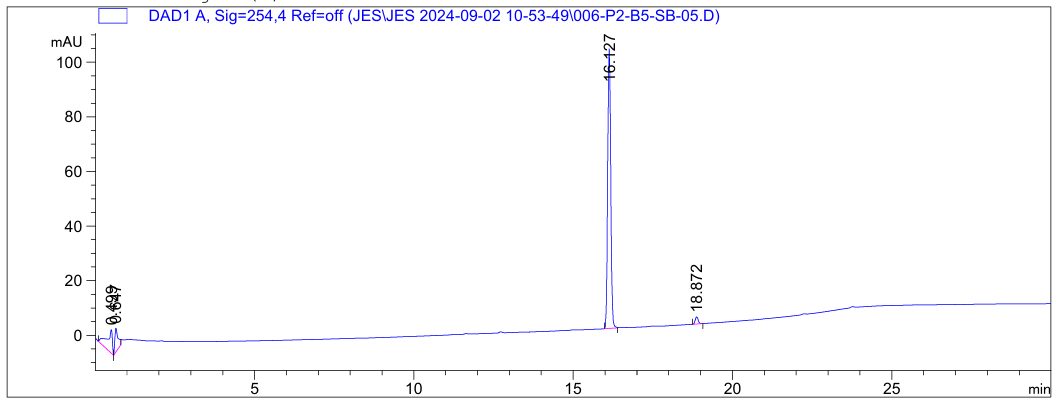


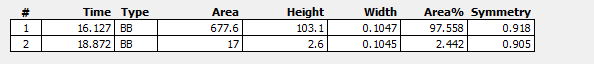


**1.3.6. SB-06 (92.349%)**: HR-MS [M]^+^: *m/z* calcd for C_18_H_19_O_7_ 347.1131, found 347.1124


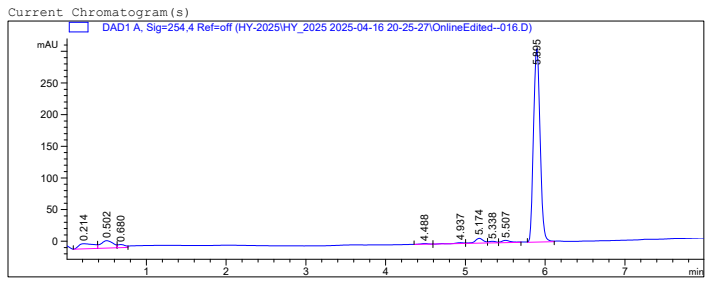


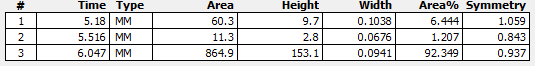


**1.3.7. SB-07 (84.970%)**: HR-MS [M]^+^: *m/z* calcd for C_17_H_17_O_7_ 333.0974, found 333.0965


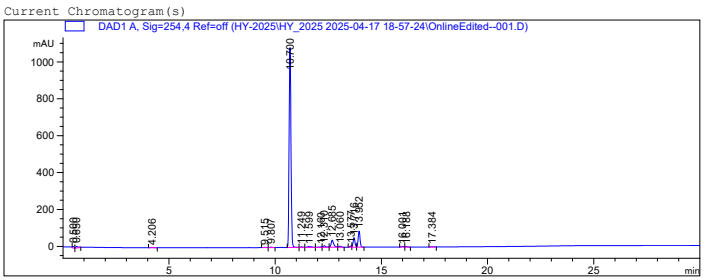


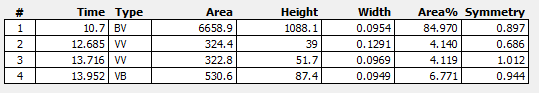


**1.3.8. SB-08 (Purity 76.945%)**: HR-MS [M]^+^: *m/z* calcd for C_39_H_37_O_7_ 617.2539, found 617.2523


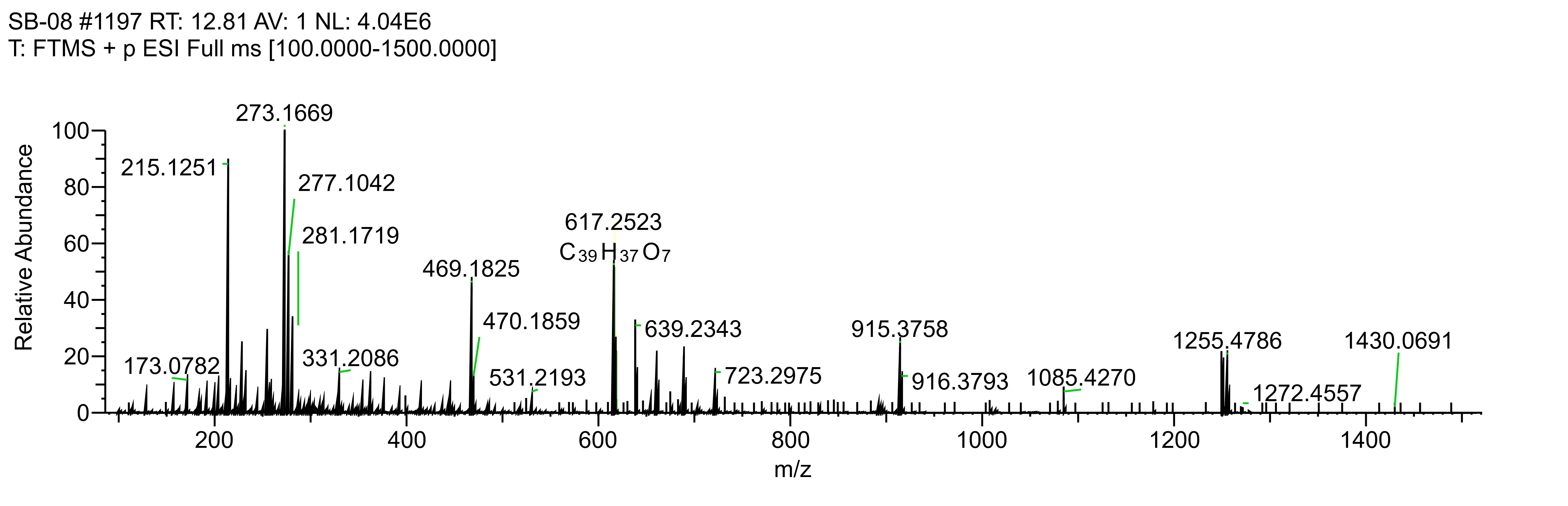


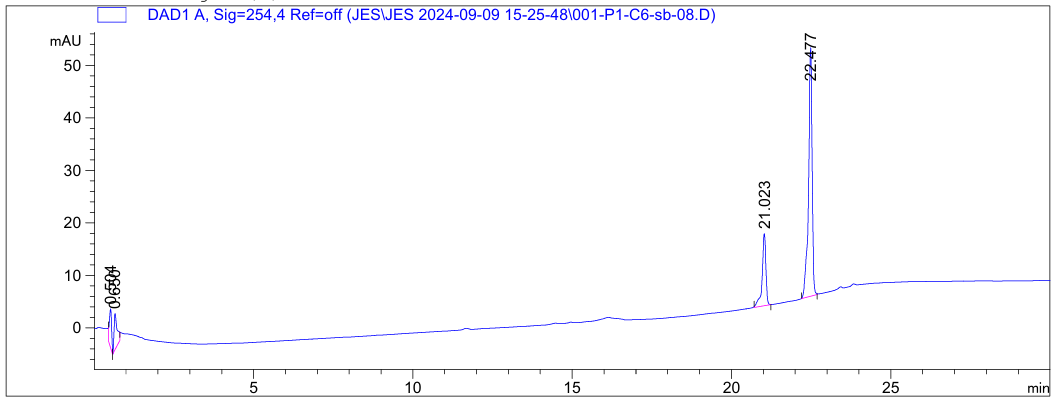


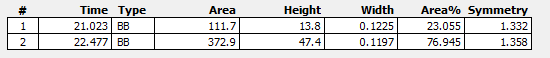


**1.3.9. SB-09 (94.356%)**: HR-MS [M]^+^: *m/z* calcd for C_33_H_33_O_7_ 541.2226, found 541.2213


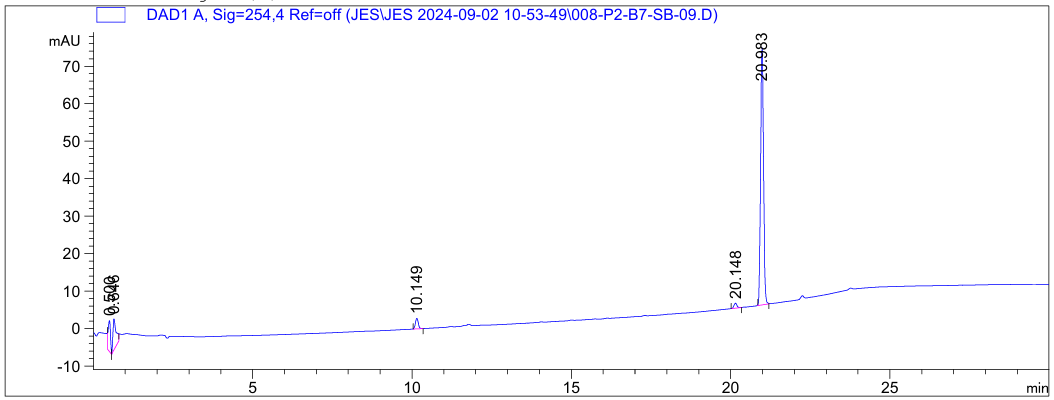


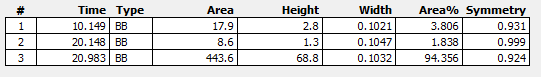


**1.3.10. SB-10 (99.99%)**: HR-MS [M]^+^: *m/z* calcd for C_19_H_21_O_7_ 361.1287, found 361.1277


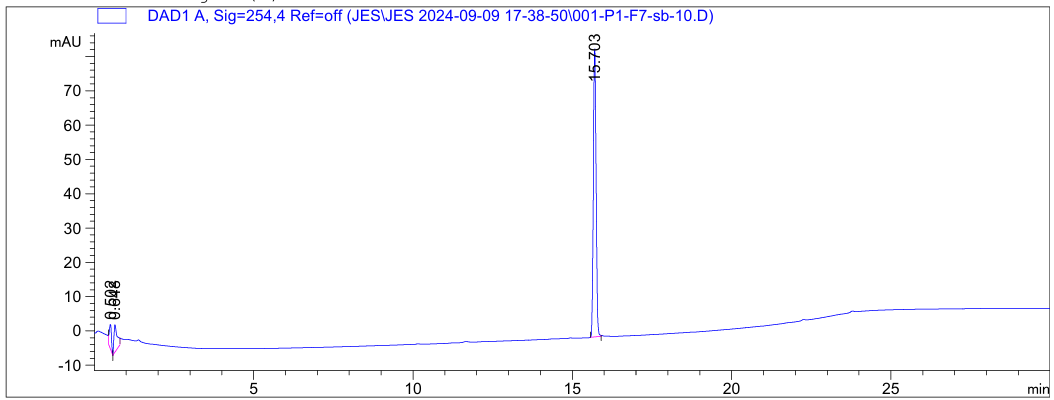


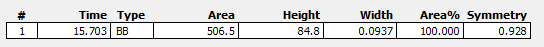


**1.3.11. SB-11 (Purity 95.659%)**: HR-MS [M]^+^: *m/z* calcd for C_18_H_19_O_7_ 347.1131, found 347.1122


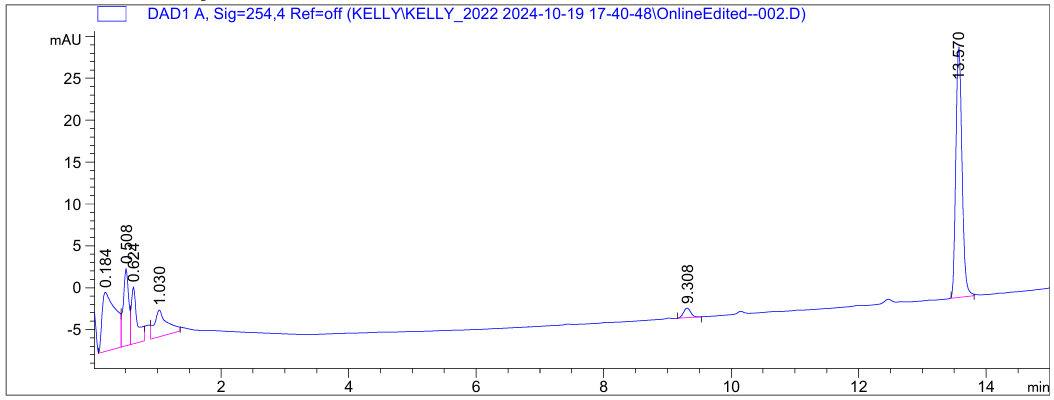


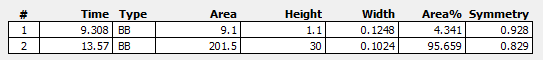


**1.3.12. SB-12 (Purity 99.99%)**: HR-MS [M]^+^: *m/z* calcd for C_25_H_24_O_7_ 437.1600, found 437.1588


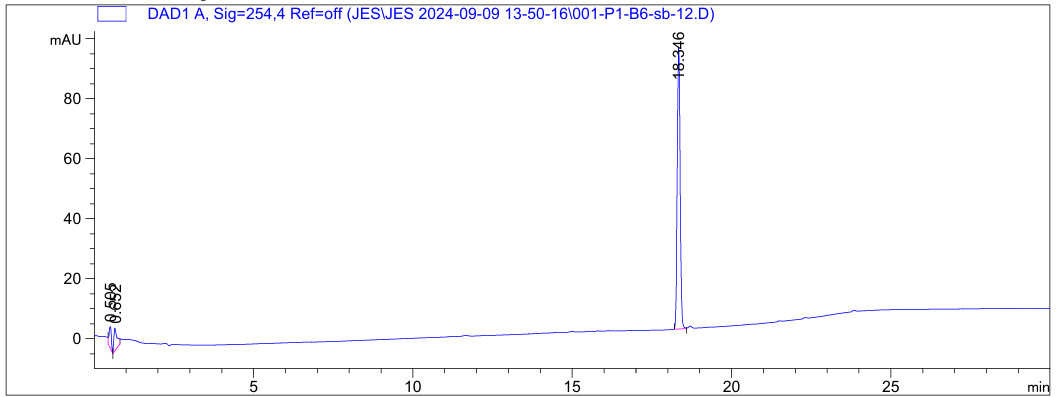


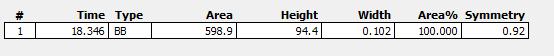


**1.3.13. SB-13 (Purity 97.700%)**: HR-MS [M]^+^: *m/z* calcd for C_29_H_33_O_7_ 493.2226, found 493.2213


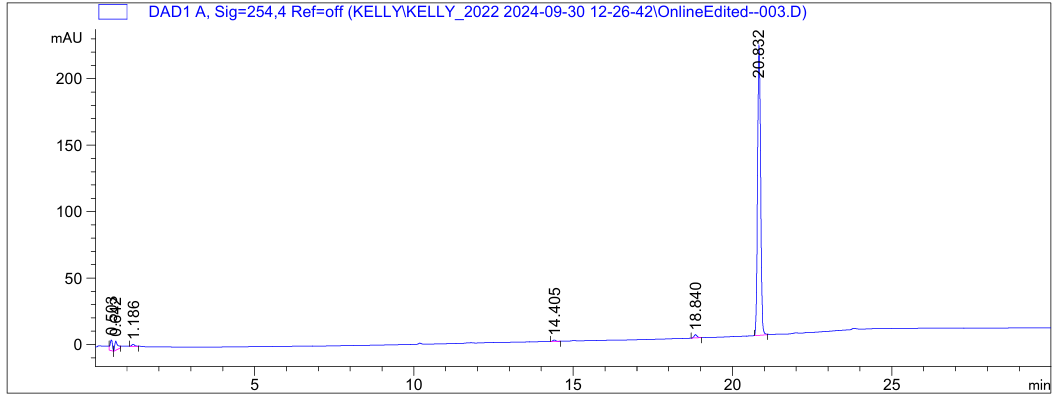


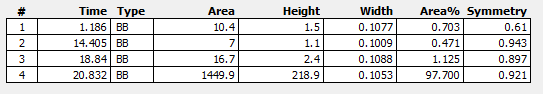


**1.3.14. SB-14 (Purity 93.559%)**: HR-MS [M]^+^: *m/z* calcd for C_35_H_37_O_7_ 569.2539, found 569.2524


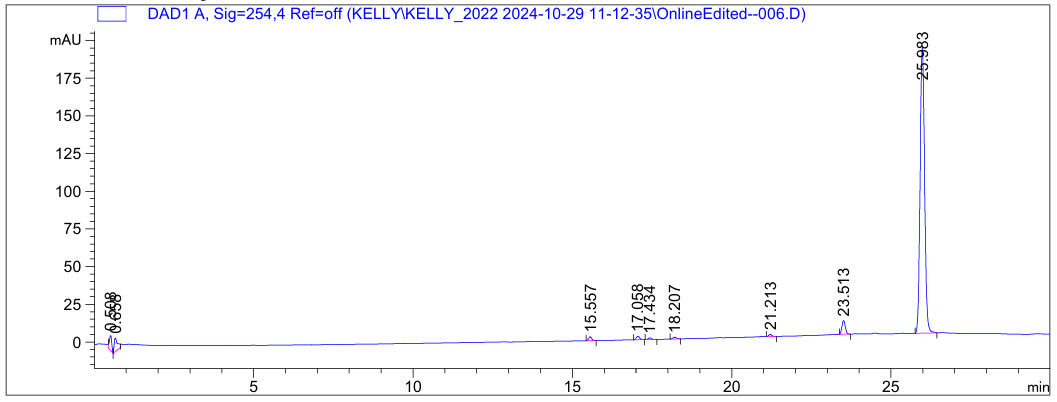


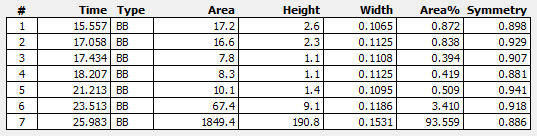


**1.3.15. SB-15 (Purity 96.351%)**: HR-MS [M]^+^: *m/z* calcd for C_34_H_35_O_7_ 555.2383, found 555.2367


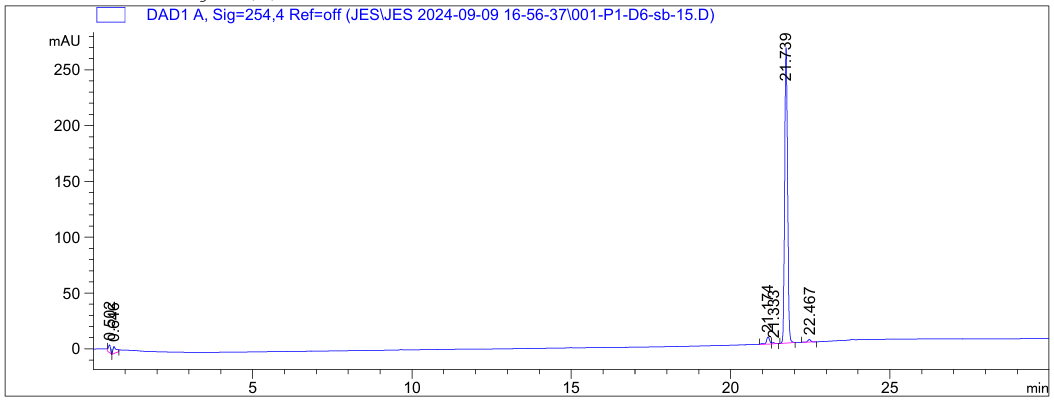


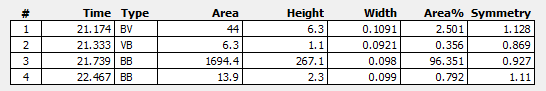


**2. Synthesis of linker-conjugated compounds**

**Scheme 12.**Structure of linker-conjugated compounds

**2.1. General procedure for the synthesis of linker-conjugated diffractaic acid analogs**

**Scheme 13.**Synthetic Scheme of DF-L analogs

To a solution of 3-hydroxy-4-(methoxycarbonyl)-2,5-dimethylphenyl 2,4-dimethoxy-3,6-dimethylbenzoate (1 eq.) in DMF were added K_2_CO_3_ (1.5 eq.) and alkyl halide (2 eq.) at the room temperature under an argon atmosphere. After being stirred at 50^0^C for 10 h, the reaction mixture was filtered through a pad of celite. The filtrate was diluted with EtOAc and acidified with 3 M HCl. The organic layer was separated, and the aqueous layer was extracted twice with EtOAc. The combined organic layers were washed with brine twice; saturated aq. sodium bicarbonate and brine dried with MgSO_4_ and filtrated. The filtrate was concentrated in a vacuum, and the resulting residue was purified by flash column chromatography on silica gel (eluted with hexane/EtOAc ) to afford linker-conjugated diffractaic acid analogs.

**2.1.1. SB-05-33 compound**

**^1^**H-NMR (400 MHz, CDCl_3_) *δ* 6.81 (s, 1H), 6.52 (s, 1H), 4.09-4.05 (m, 4H), 3.91 (s, 3H), 3.84 (s, 3H), 3.73-3.61 (m, 4H), 3.43 (s, 3H), 2.44 (s, 3H), 2.31-2.29 (m, 3H), 2.21 (s, 3H), 2.17 (s, 3H), 2.03 (s, 3H).

**2.1.2. DF-L-01 compound**

^1^H-NMR (400 MHz, CDCl_3_) δ 6.84 (s, 1H), 6.54 (s, 1H), 6.37 (s, 1H), 4.03 (t, J = 4.8 Hz, 2H), 3.92 (s, 3H), 3.86 (s, 3H), 3.83 (s, 3H), 3.59 (q, J = 5.0 Hz, 2H), 2.46 (s, 3H), 2.32 (s, 3H), 2.19 (s, 3H), 2.16 (s, 3H), 2.04 (s, 3H)

**2.1.3. DF-L-02 compound**

^1^H-NMR (400 MHz, CDCl_3_) δ 6.88 (s, 1H), 6.53 (s, 1H), 4.43 (s, 2H), 3.88 (s, 3H), 3.85 (s, 3H), 3.82 (s, 3H), 3.58-3.63 (m, 4H), 3.52 (t, J = 5.0 Hz, 2H), 3.40 (q, J = 5.2 Hz, 2H), 2.45 (s, 3H), 2.31 (s, 3H), 2.17 (s, 3H), 2.15 (s, 3H), 1.92 (s, 3H)

**2.1.4. DF-L-03 compound**

^1^H-NMR (400 MHz, CDCl_3_) δ 6.88 (s, 1H), 6.53 (s, 1H), 4.43 (s, 2H), 3.88 (s, 3H), 3.85 (s, 3H), 3.82 (s, 3H), 3.50-3.53 (m, 2H), 3.46 (t, J = 5.7 Hz, 2H), 2.45 (s, 3H), 2.32 (s, 3H), 2.18 (s, 3H), 2.15 (s, 3H), 2.01 (s, 3H)

**2.1.5. DF-L-04 compound**

^1^H-NMR (400 MHz, CDCl_3_) ) δ 6.89 (s, 1H), 6.54 (s, 1H), 5.81 (s, 1H), 4.42 (s, 2H), 3.89 (s, 3H), 3.87 (s, 3H), 3.83 (s, 3H), 3.40 (q, J = 6.6 Hz, 2H), 3.31 (q, J = 6.3 Hz, 2H), 2.46 (s, 3H), 2.33 (s, 3H), 2.18 (s, 3H), 2.16 (s, 3H), 1.97 (s, 3H), 1.61-1.70 (m, 4H)

**2.1.6. DF-L-05 compound**

^1^H-NMR (400 MHz, CDCl_3_) δ 6.88 (s, 1H), 6.54 (s, 1H), 5.63(s, 1H), 4.41 (t, J = 16.5 Hz, 2H), 3.89 (s, 3H), 3.86 (s, 3H), 3.83 (s, 3H), 3.35-3.42 (m, 2H), 3.25 (q, J = 6.6 Hz, 2H), 2.46 (s, 3H), 2.33 (s, 3H), 2.19 (s,3H), 2.16 (s, 3H), 1.98 (s, 3H), 1.39 (m, 8H)

**2.1.7. DF-L-06 compound**

^1^H-NMR (400 MHz, CDCl_3_) δ 6.83 (s, 1H), 6.53 (s, 1H), 5.11(s, 1H), 4.04-4.09 (m, 2H), 3.93 (s, 3H), 3.86 (s, 3H), 3.83 (s, 3H), 3.75 (t, J = 4.3 Hz, 2H), 3.65 (t, J = 4.8 Hz, 2H), 3.33 (q, J = 5.0 Hz, 2H), 2.96 (s, 3H), 2.46 (s, 3H), 2.30 (s, 3H), 2.21 (s, 3H), 2.15 (s, 3H)

**2.1.8. DF-L-07 compound**

^1^H-NMR (400 MHz, CDCl_3_) δ 6.83 (s, 2H), 4.09 (t, J = 4.3 Hz, 2H), 3.92 (s, 3H), 3.84 (s, 3H), 3.83 (s, 3H), 3.78 (d, J = 4.1 Hz, 2H), 3.69 (d, J = 4.6 Hz, 2H), 3.66 (d, J = 4.6 Hz, 2H), 3.58 (t, J = 4.8 Hz, 2H), 3.47 (t, J = 5.5 Hz, 2H), 2.46 (s, 3H), 2.31 (s, 3H), 2.28 (s, 3H), 2.22 (s, 3H), 1.98 (s, 3H)

**2.1.9. DF-L-08 compound**

^1^H-NMR (400 MHz, CDCl_3_) δ 6.83 (s, 1H), 6.53 (s, 1H), 3.97 (t, J = 5.5 Hz, 2H), 3.90 (s, 3H), 3.86 (s, 3H), 3.83 (s, 3H), 3.46 (q, J = 5.8 Hz, 2H), 2.46 (s, 3H), 2.30 (s, 3H), 2.18 (s, 3H), 2.15 (s, 3H), 2.03 (s, 3H), 1.91 (t, J = 5.9 Hz, 2H)

**Scheme 14.**Synthetic Scheme of  DF-L-01 & 08 immobilized with affigel-10;(i) tert-butyl (2-bromoethyl)carbamate(DF-L-01 Amine), tert-butyl (3-bromopropyl)carbamate(DF-L-08 Amine), K_2_CO_3_, DMF, 50^o^C, 10h. (ii) TFA, DCM, rt, 12h. (iii) Affigel-10, DMSO, rt,4h

**2.2. General procedure for the synthesis of linker-conjugated Affigel beads**

To a solution of 3-hydroxy-4-(methoxycarbonyl)-2,5-dimethylphenyl 2,4-dimethoxy-3,6-dimethylbenzoate (1eq.) in DMF were added K_2_CO_3_ (1.5 eq.) and bromo carbamate (2 eq.) at the room temperature under an argon atmosphere. After being stirred at 50 ^0^C for 10 h, the reaction mixture was filtered through a pad of celite. The filtrate was diluted with EtOAc and acidified with 3 M HCl. The organic layer was separated, and the aqueous layer was extracted twice with EtOAc. The combined organic layers were washed with brine twice; saturated aq. sodium bicarbonate and brine dried with MgSO_4_ and filtrated. The filtrate was concentrated in a vacuum, and the resulting residue was purified by flash column chromatography on silica gel (eluted with hexane/EtOAc) to DF-L Boc-Amine.

De-Boc Procedure ; To a solution of Boc-Amine (1eq.) dissolved into an anhydrous DCM was slowly added TFA at 0 ^o^C Transfer the reaction mixture at room temperature and stirred for an 2 h. after completion of reaction evaporate the solvents and dried by high vacuum.

**2.2.1. DF-L-01 Boc-Amine.**

^1^H-NMR (400 MHz, CDCl_3_) *δ* 6.89 (s, 1H), 6.54 (s, 1H), 5.81 (s, 1H), 3.89-3.86 (m, 6H), 3.83 (s, 3H), 3.60 (q, J = 6.2 Hz, 2H), 2.46 (s, 3H), 2.33 (s, 2H), 2.18-2.16 (m, 6H), 1.97 (d, J = 14.6 Hz, 3H), 1.4 (s, 9H).

**2.2.2. DF-L-08 Boc-Amine.**

^1^H-NMR (400 MHz, CDCl_3_) *δ* 6.89 (s, 1H), 6.50 (s, 1H), 5.80 (s, 1H), 3.87-3.82 (m, 6H), 3.89 (s, 3H), 3.60 (q, J = 6.2 Hz, 2H), 2.46 (s, 3H), 2.30 (m, 4H), 2.20-2.18 (m, 6H), 1.98 (d, J = 14.6 Hz, 3H), 1.5 (s, 9H).

**2.3. General Synthesis procedure for DF-L-01 & DF-L-08 immobilized Affigel-10**

Affigel-10 was transferred into a 3 mL cartridge with a polyethylene frit. The supernatant solvent was drained, and the affi-gel (10 μmol) was washed with DMSO. A solution of the free-amine linker version of the DF-L-01, DF-L-08-amine (10 μmol) in DMSO and DIEA (0.250 mL) was added to the gel. The cartridge was shaken well for 4 h at RT. The resulting slurry was drained, and the gel was washed with DMSO. The loading level (90%) was determined by analyzing the eluent mixed with an internal standard by LCMS and comparing the result to the initial reaction mixture. A solution of ethanolamine in DMSO and DIEA was added to the reaction cartridge and shaken well for 3 h at RT. The resulting slurry was drained, and the gel was washed with DMSO, water, and 2% sodium azide in water. The affi-gel product was stored in a 2% sodium azide solution in water at 4 ° C.

**3. Reporter assay**

HEK293T was plated in a 24-well plate, left to attach, and then transfected with 100 ng of AP-1, STAT, Gli, CSL, TOPFLASH (with 100 ng β-catenin), NF-kB, Hes-1 and MMP9 reporter plasmids with 2 ng of Renilla-luc (pRL-TK) plasmid. MMP9 reporter assays were tested by stimulating Gli1-Flag, Gli2-Flag, ΔEN1-Myc, and MYC-flag plasmids. Transfections were performed using the X-treme GENE 9 DNA transfection reagent (Roche, Werk Penzberg, Germany). After 24 hours transfection, cells were treated with compounds or DMSO and incubated for 24 h at 37 °C under 5% CO_2_. The Dual-Luciferase reporter assay system (Promega, Madison, WI, USA) was used.

**4. Subcellular fractionation; isolation of mitochondria**

Cytosolic and mitochondrial fractions were isolated using the Mitochondria/Cytosol Fractionation Kit (Thermo Fisher Scientific) for treated cells according to the manufacturer’s instructions. After collecting the mitochondria/cytosol fraction from cells, 25 µg of cytosolic and mitochondrial proteins were separated by SDS-PAGE gel electrophoresis. Antibodies against HK2, VDAC1 and COX4 were from Cell Signaling Technology.

**5. Molecular docking simulation**

These crystal structures of the VDAC1, PHB2, MMP-9 were found in the PDB database (PDB ID: 2JK4, 6IQE, 1GKC) to investigate the interaction mechanism between the two receptors and ligand binding sites, and it was decided to use this crystal structure under the guidance of references ^1^. The molecular docking study of the SB4 and SB5 are obtained using CB-Dock (<http://cao.labshare.cn/cb-dock/>) ^2^. Downloaded docking poses were visualized in 2D and 3D with BIOVIA Discovery Studio 2021. Docking scores calculated by CB-Dock were used.

**6. Quantitative reverse transcription-quantitative polymerase chain reaction (qRT-PCR)**

Reverse transcription and RNA preparation were carried out in accordance with previously described ^3^. qRT-PCR reactions and analyses were performed using CFX (Bio-Rad, Hercules, USA). The list of primers used in this research is shown in Supplementary Table S3.

**7. Measurement of ROS Generation**

CRC cells were seeded in a 6-well plate at the density of 2 × 10^5^ cells/well for overnight and treated with DMSO or compounds for 12 h in 2% FBS media. After that, cells were incubated with DCFH-DA (10 µM) in medium without FBS for 30 min at 37 °C and washed three times with DMEM. ROS generation was determined by fluorescence microscopy (K1-Fluo Confocal Laser Scanning Microscope, Nanoscope Systems, Daejeon, Republic of Korea).

**8. Caspase 3/7 activity**

Caspase 3/7 activity was determined with a Caspase-Glo 3/7 assay kit (Promega). CRC cells were seeded in 96 well plates, 10000 per well cells, with media containing 2% FBS. Treatments were applied after overnight incubation, incubated for 48 hours. After treatment, Caspase-Glo reagent was added to each well and incubated in the dark for 1 hour. Fluorescence intensity was recorded with the GloMax® Microplate Reader.

**9. Bioinformatics mining**

The online database GEPIA (Gene Expression Profiling Interactive Analysis, <http://gepia.cancerpku.cn/index.html.>) was used to analyze the RNA sequencing expression data related to our project based on The Cancer Genome Atlas (TCGA) and/or the Genotype-Tissue Expression (GTEx) projects ^4^. GEPIA performs survival analyses, expression level of subtype of COAD cancer vs normal tissue, COAD different cancer stages. The p value < 0.05 was noted statistically significant. Human Protein Atlas cancer databases (<https://www.proteinatlas.org/>) were mined to predict the target gene expression on different cell line. Cellosaurus (a knowledge resource on cell lines, <https://www.cellosaurus.org/index.html>) were used for getting information about the cell line harboring different genetic mutations. STRING (Search-Tool-for-the-Retrieval-of-Interacting-Genes/Proteins, https://string-db.org/) database was used for the functional analysis of interactions ^5^. Physicochemical, pharmacokinetic, and ADME predictions are provided by the Swiss ADME web tool ^6–8^.

**References**

1. Tabti K, Ahmad I, Zafar I, et al. Profiling the structural determinants of pyrrolidine derivative as gelatinases (MMP-2 and MMP-9) inhibitors using in silico approaches. *Comput Biol Chem*. 2023;104:1476-9271.

2. Liu Y, Grimm M, Dai W tao, Hou M chun, Xiao ZX, Cao Y. CB-Dock: a web server for cavity detection-guided protein–ligand blind docking. *Acta Pharmacol Sin*. 2020;41(1):138-144.

3. Varlı M, Kim SJ, Noh MG, et al. KITENIN promotes aerobic glycolysis through PKM2 induction by upregulating the c-Myc/hnRNPs axis in colorectal cancer. *Cell Biosci*. 2023;13(1):1-21.

4. Tang Z, Li C, Kang B, Gao G, Li C, Zhang Z. GEPIA: a web server for cancer and normal gene expression profiling and interactive analyses. *Nucleic Acids Res*. 2017;45(W1):W98-W102.

5. Szklarczyk D, Gable AL, Lyon D, et al. STRING v11: protein–protein association networks with increased coverage, supporting functional discovery in genome-wide experimental datasets. *Nucleic Acids Res*. 2019;47(D1):D607-D613.

6. Daina A, Michielin O, Zoete V. SwissADME: a free web tool to evaluate pharmacokinetics, drug-likeness and medicinal chemistry friendliness of small molecules. *Scientific Reports 2017 7:1*. 2017;7(1):1-13.

7. Daina A, Michielin O, Zoete V. ILOGP: A simple, robust, and efficient description of n-octanol/water partition coefficient for drug design using the GB/SA approach. *J Chem Inf Model*. 2014;54(12):3284-3301.

8. Daina A, Zoete V. A BOILED-Egg To Predict Gastrointestinal Absorption and Brain Penetration of Small Molecules. *ChemMedChem*. 2016;11(11):1117-1121.

**Supplementary Figures**

**
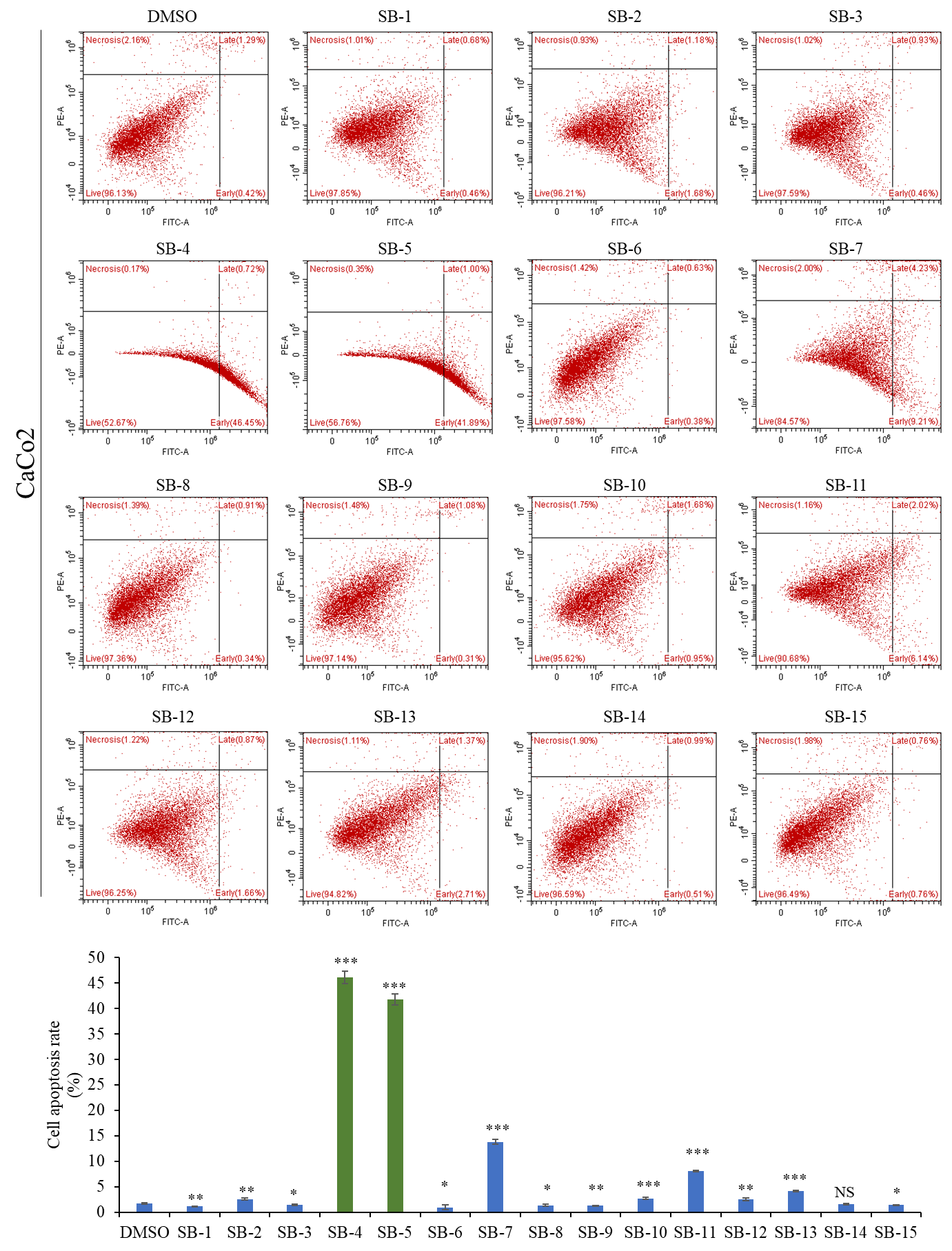
**

**Supplementary Figure S1. Apoptosis test of SB compounds on CaCo2.** Apoptotic cell populations using a CytoFLEX Flow Cytometer, cells were stained with Annexin V-FITC/PI. Quantification of the percentage of total apoptotic cells treated with the indicated compounds at 10 μM concentration for 48 hours. Data are presented as the mean ± standard deviation, n=3. **p* < 0.05; ***p* < 0.01; ****p* < 0.001; NS, no significant difference between compared DMSO.


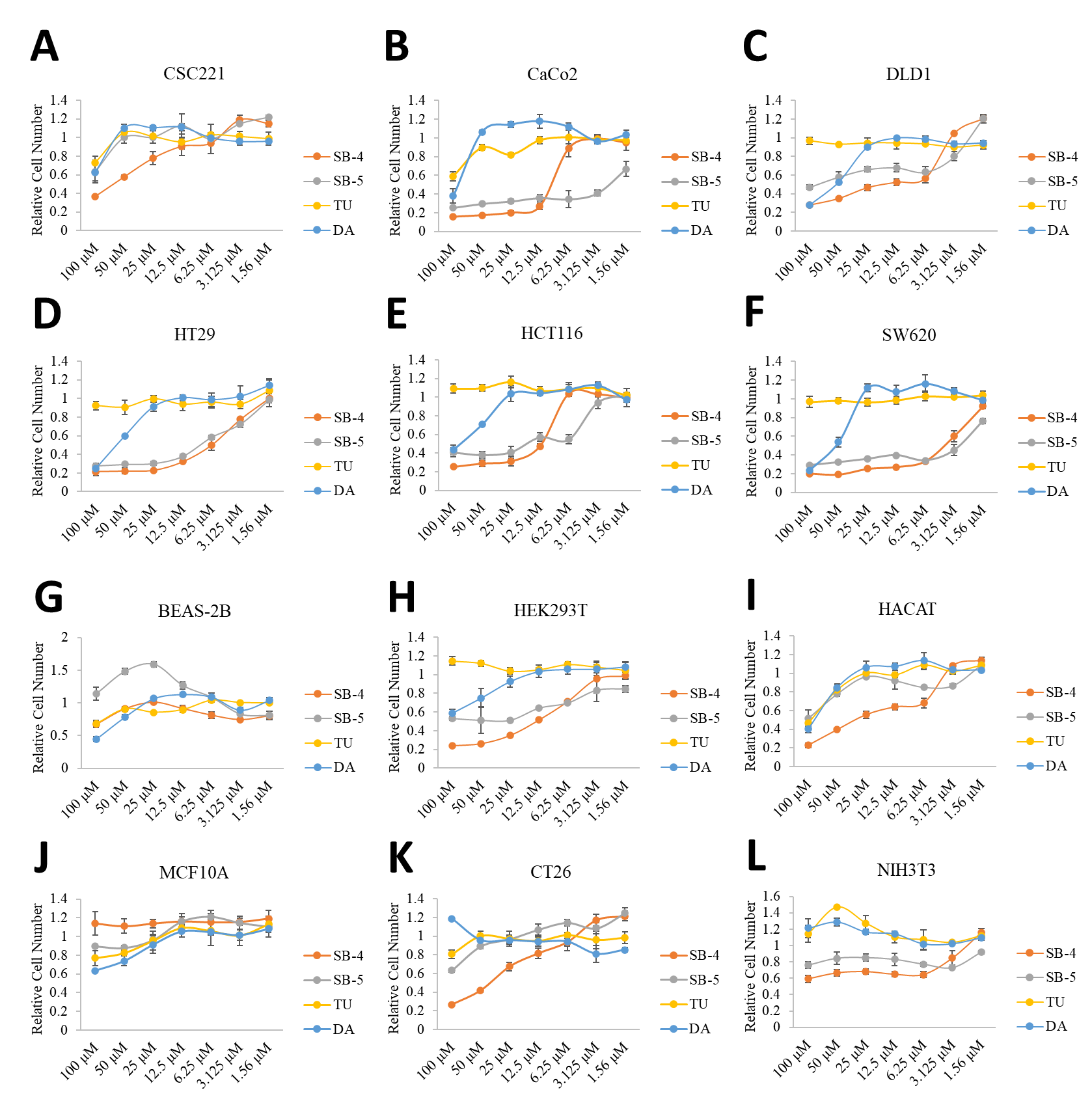


**Supplementary Figure S2. Cytotoxic screening in cancerous and non-cancerous cell lines.** CSC221, CaCo2, DLD1, HT29, HCT116, SW620, BEAS-2B, HEK293T, HaCaT, MCF10A CT26, and NIH3T3 cells were treated with compounds for 48 hours, and cell viability was measured by MTT assay. Data are presented as the mean ± standard deviation, n=3.

**
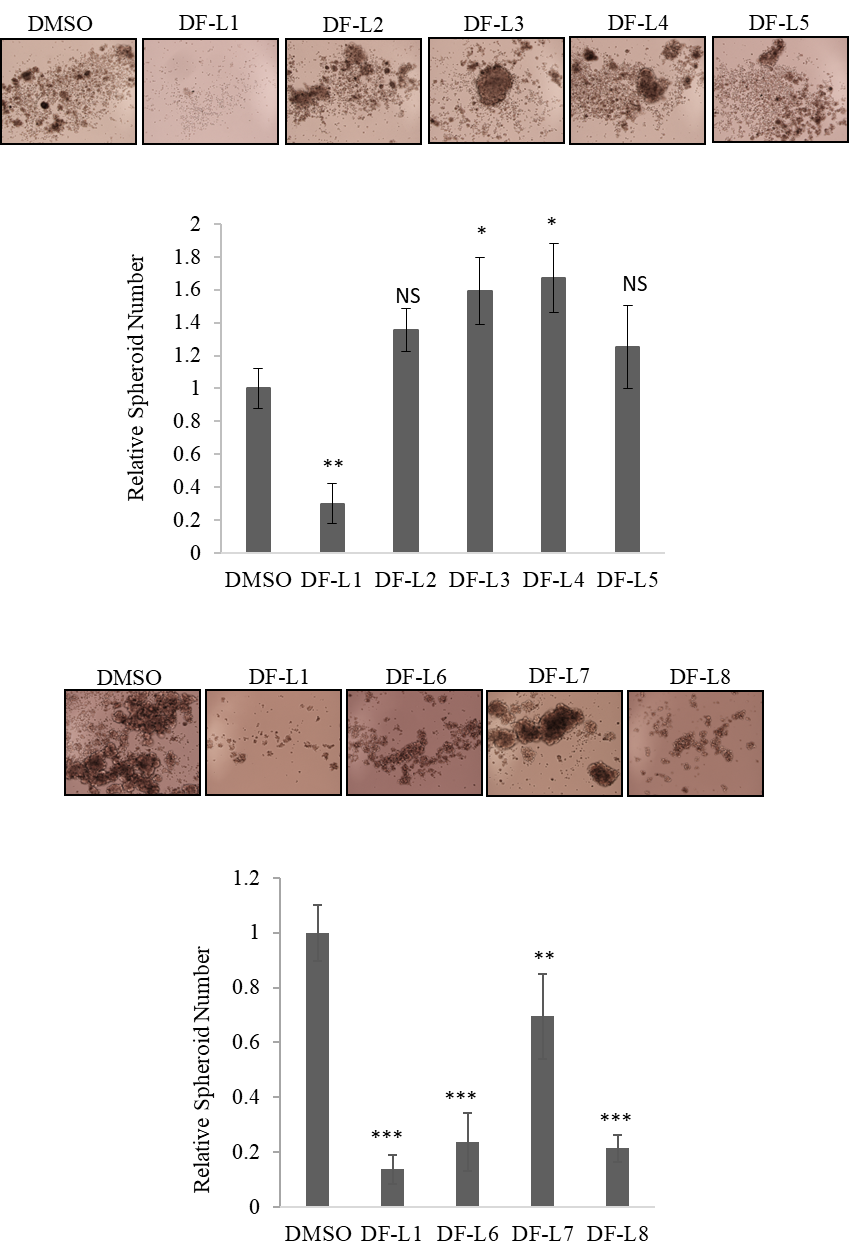
**

**Supplementary Figure S3. Synthesis of linker-conjugated compounds (DF-L1 – DF-L8) effect on CRC stemness.** Cells were exposed to the compound at the indicated concentrations (10 μM) for 14 days. The histogram represents spheroid formation, calculated as rate relative to vehicle-treated control, and represented as bar graphs. Data are presented as the mean ± standard deviation, **p* < 0.05; ***p* < 0.01; ****p* < 0.001; NS, no significant difference between compared DMSO**.**

**
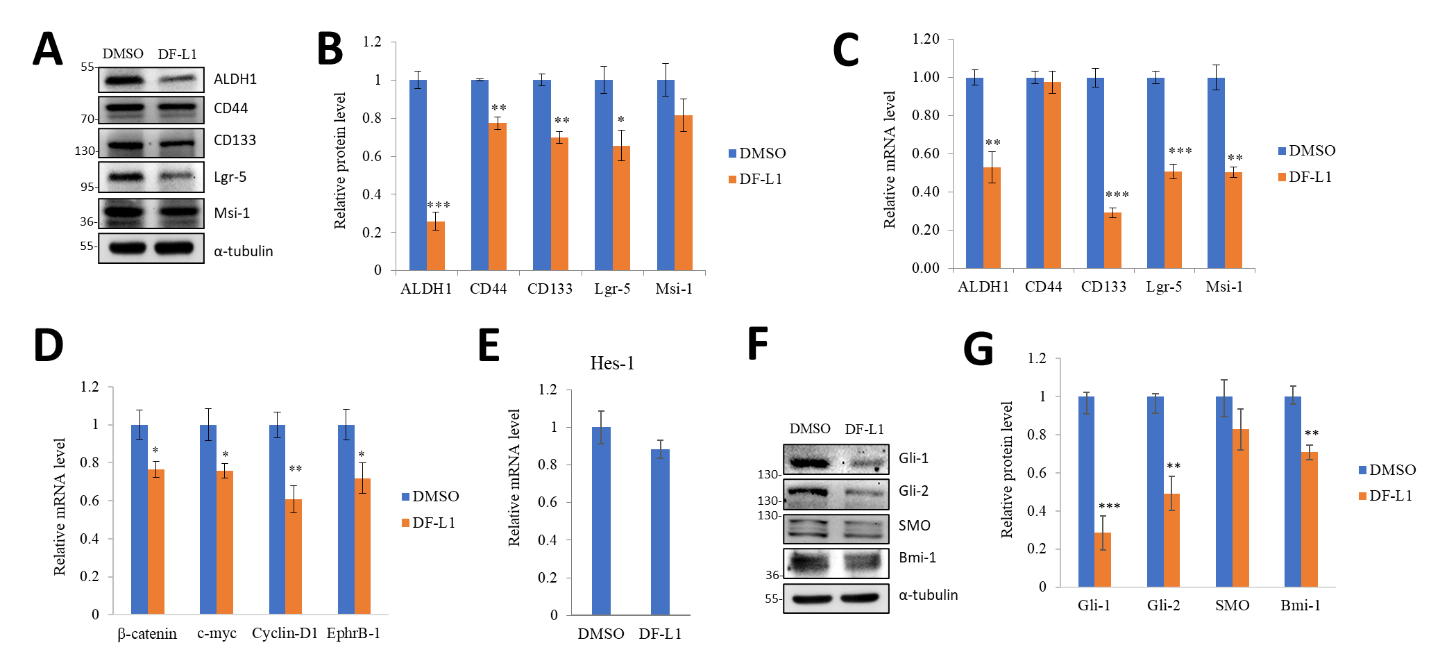
Supplementary Figure S4. Characterization of the effects of the linker compound DF-L1 on CRC stem cell inhibition.** CSC221 cells were treated for 48 h with DF-L1 (10 μM) **(A-C)** Protein level and quantitative analysis of mRNA encoding cancer stem markers aldehyde dehydrogenase-1 (ALDH1), cluster of differentiation 133 (CD133), CD44, Lgr5, Musashi-1. **(D)** mRNA level of *β-catenin, c-Myc, Cyclin-D1* and *EphrB-1*. **(E)** *Hes-1* mRNA levels in CSC221 cells treated with DF-L1 (10 μM). **(F-G)** Western blot analysis of Gli1, Gli2, SMO, and Bmi-1 protein levels in CSC221 cells treated with DF-L1 (10 μM) and incubated for 48 h. Quantitative analysis of protein expression were given in the figure. Data are presented as the mean ± standard deviation. **p* < 0.05; ***p* < 0.01; ****p* < 0.001; NS, no significant difference between compared groups.

**
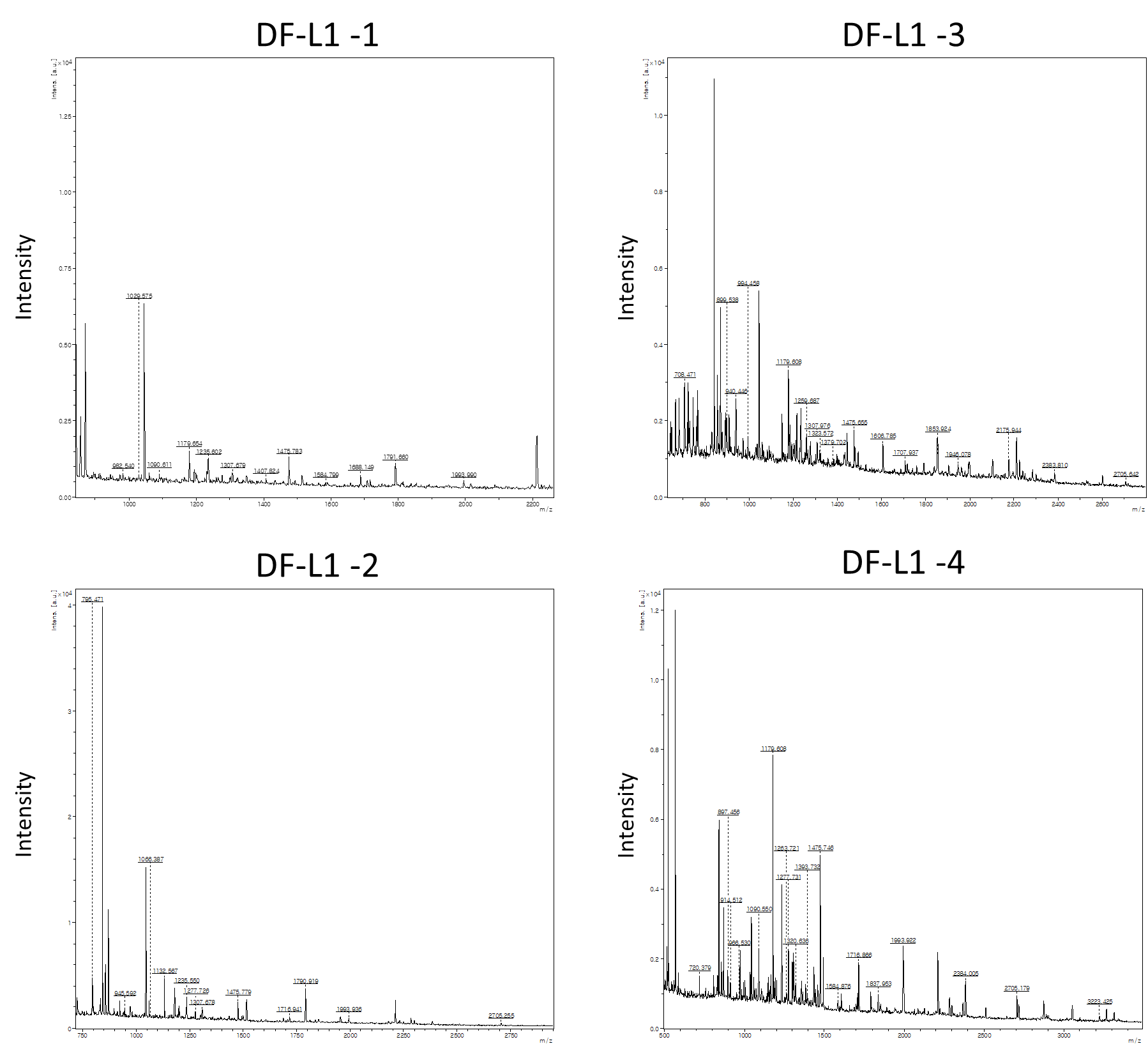
**

**Supplementary Figure S5.** **The identification of the target protein SB compounds by using DF-L-01 immobilized Affigel-10.** Indicated protein spots in the Figure 2 (DF-L1 panel) were excised to identify the proteins using peptide mass fingerprinting and subjected MALDI TOF analysis. Mass spectra data are given in the figure.

**
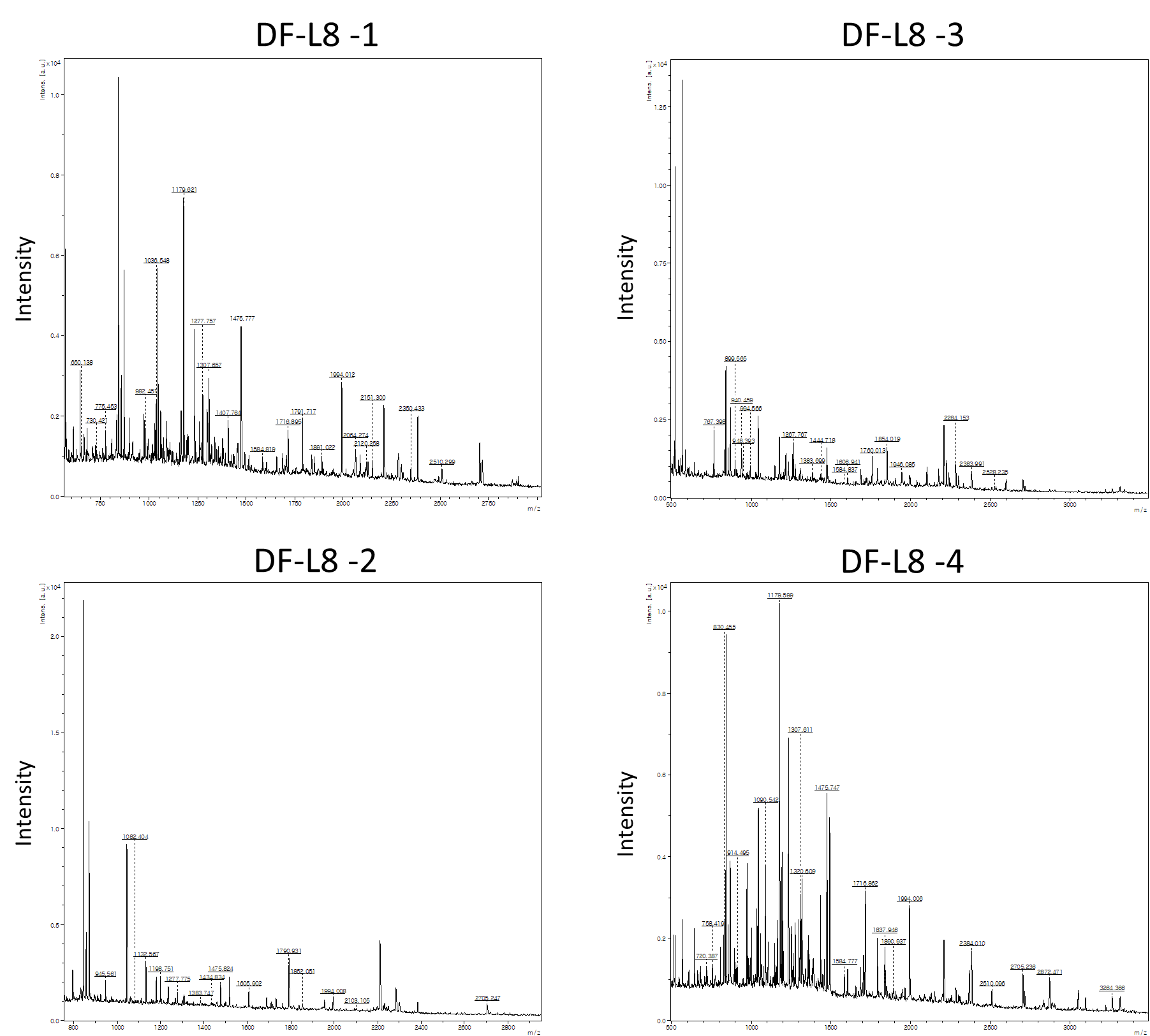
**

**Supplementary Figure S6. The identification of the target protein SB compounds by using DF-L-08 immobilized Affigel-10.** Indicated protein spots in the Figure 2 (DF-L8 panel) were excised to identify the proteins using peptide mass fingerprinting and subjected MALDI TOF analysis. Mass spectra data are given in the figure.

**
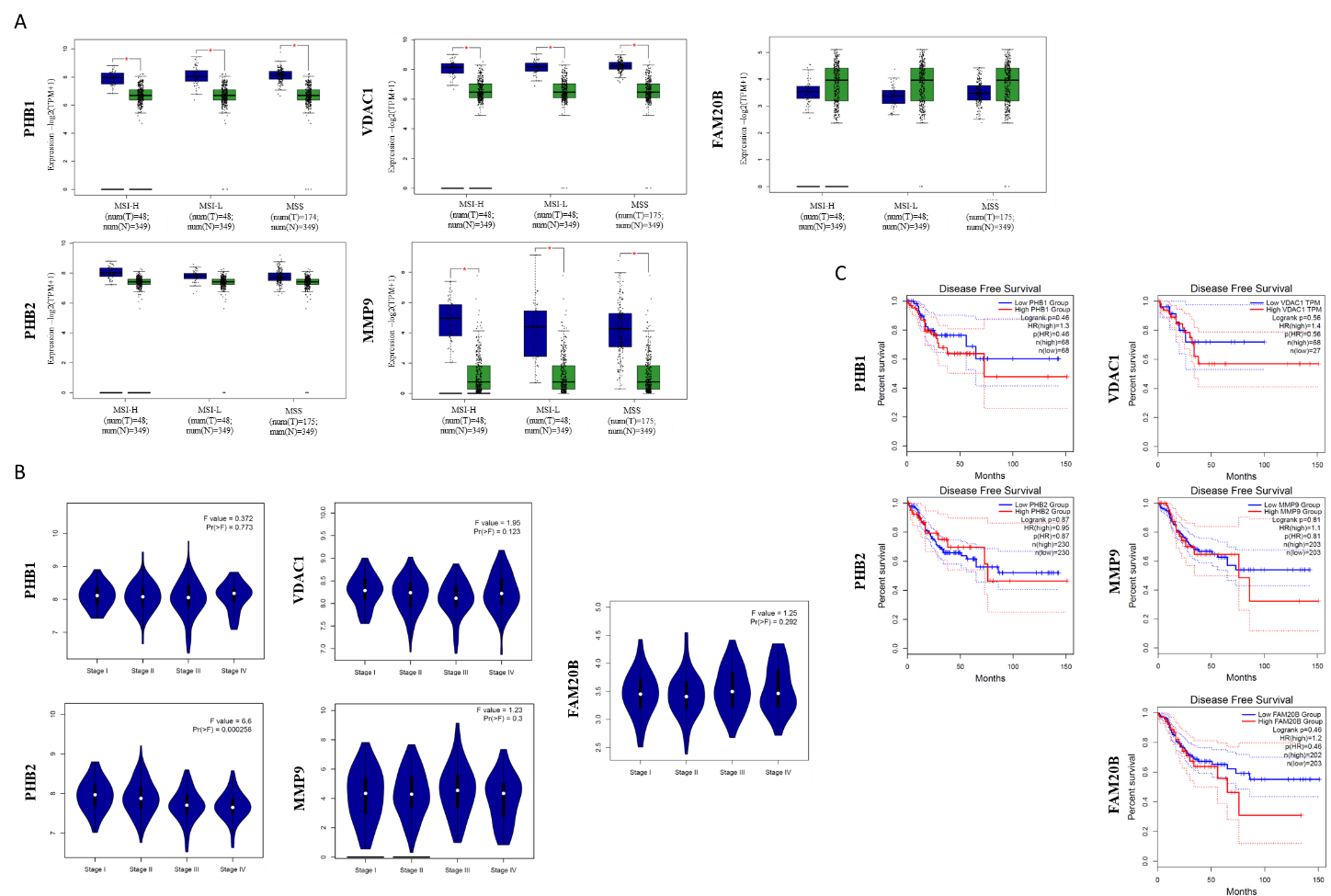
Supplementary Figure S7. Revealing the clinical significance of targets.** (A) Comparison of the expressions of *PHB1, PHB2, VDAC1, MMP9* and *FAM20B* in tumor tissues and normal tissues according to CRC subtypes using the GEPIA web tool. (B) Expression of *PHB1, PHB2, VDAC1, MMP9* and *FAM20B* in different clinical stages of CRC patients. (C) GEPIA web tool was searched for the disease-free survival of high vs low levels of SB`s compounds target gene.

**
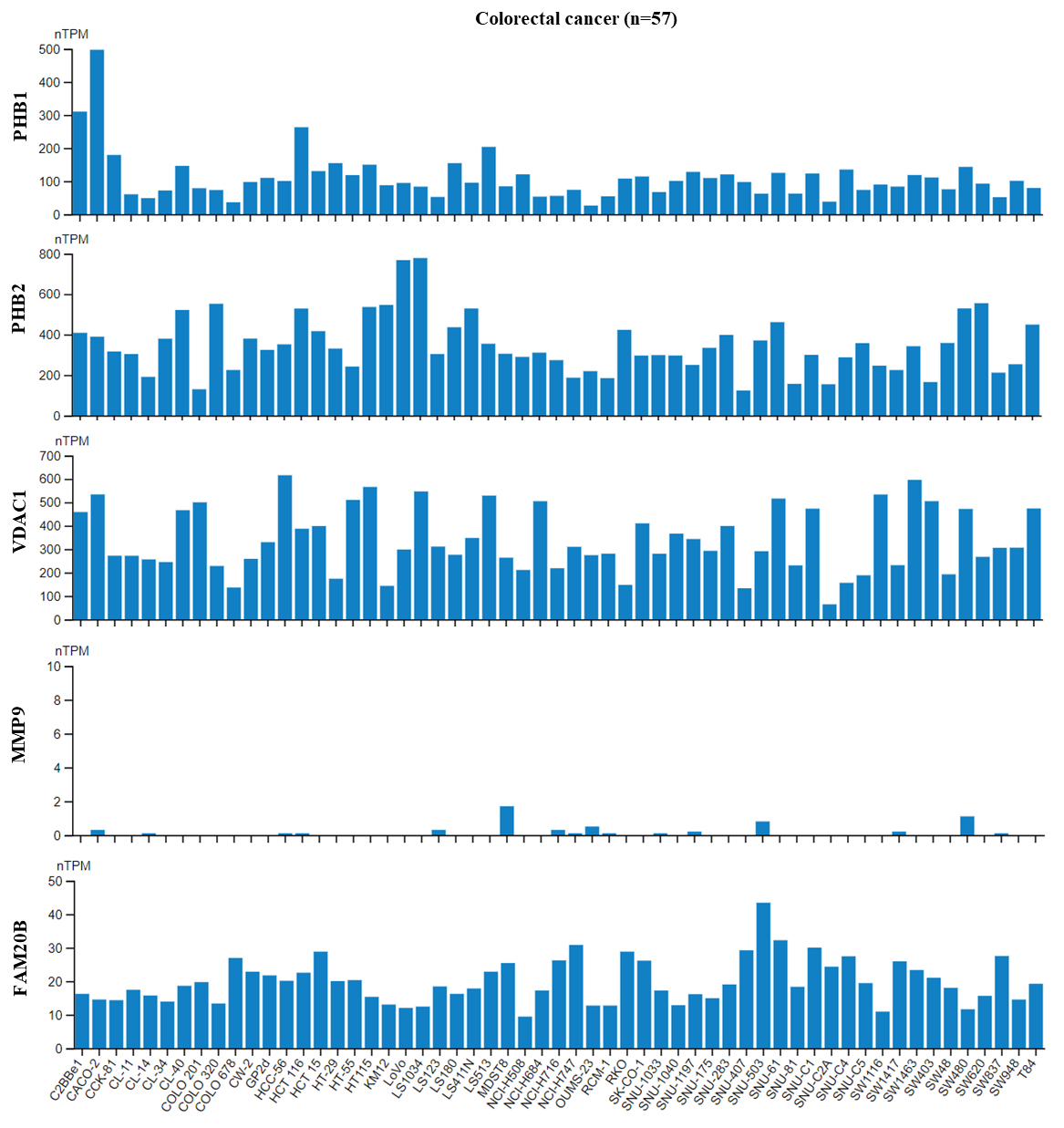
**

**Supplementary Figure S8.** ***PHB1, PHB2, VDAC1, MMP9* and *FAM20B* expression level in CRC cell lines.**

**
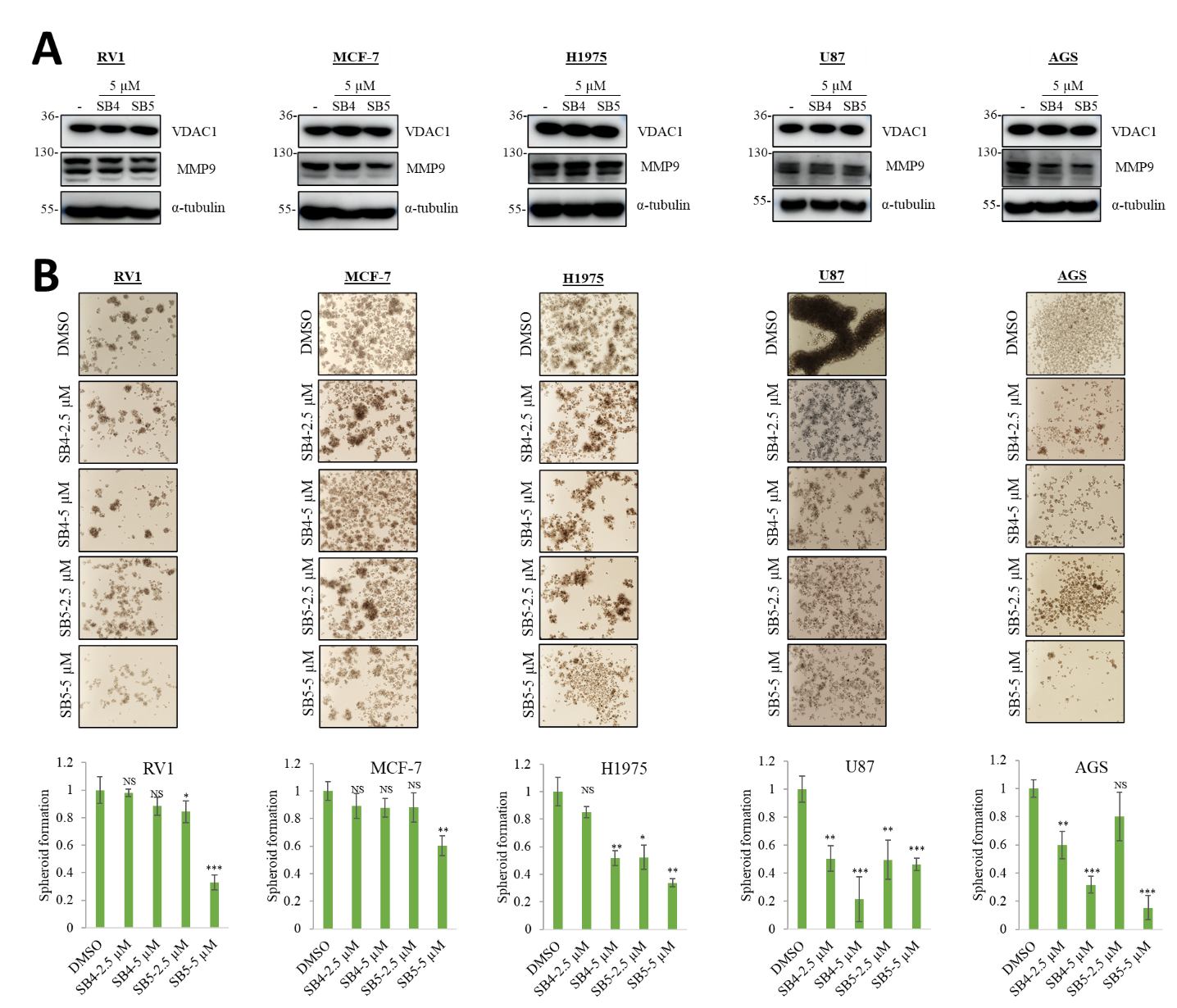
Supplementary Figure S9.** (A) VDAC1 and MMP9 immunoblots are shown. Expression of each target protein after cells were exposed SB4 and SB5 for 48 hours. (B) Cells were exposed to the compound at 2.5 and 5 μM concentration for 10-14 days. The histogram represents spheroid formation, calculated as rate relative to vehicle-treated control, and represented as bar graphs. Data are presented as the mean ± standard deviation. **p* < 0.05; ***p* < 0.01; ****p* < 0.001; NS, no significant difference between compared groups.


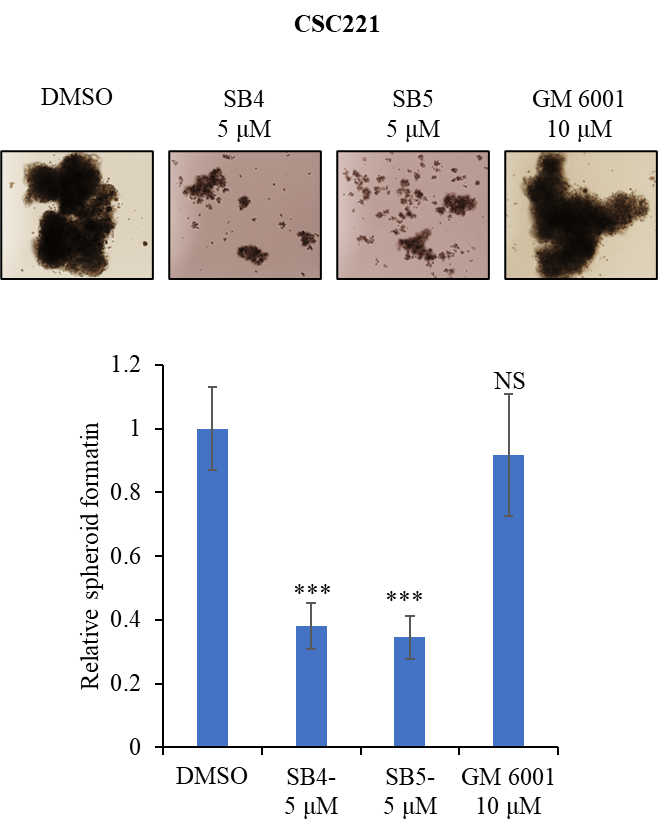


**Supplementary Figure S10.** **Comparison of the effects of novel VDAC1/PHB/MMP9 binding compounds and known MMP inhibitor on spheroid formation.** Cells were exposed to the compound at 5 μM concentration of SB4 or SB5 and 10 μM of MMP inhibitor (GM 6001, ilomastat) for 10-14 days. The histogram represents spheroid formation, calculated as rate relative to vehicle-treated control, and represented as bar graphs. **p* < 0.05; ***p* < 0.01; ****p* < 0.001; NS, no significant difference between compared groups.

**
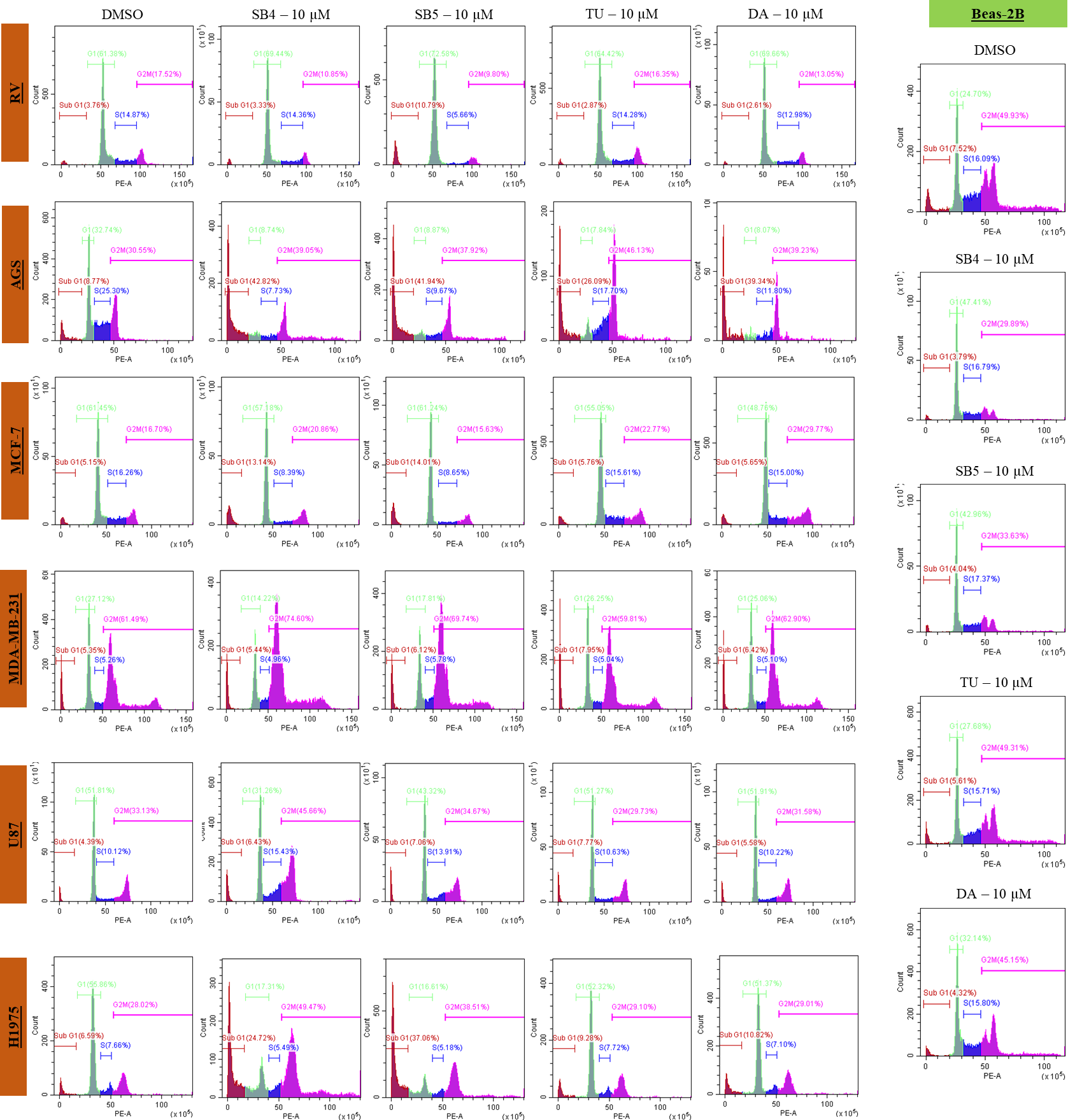
Supplementary Figure S11.** Using flow cytometry, the cell-cycle distribution was evaluated on RV, AGS, MCF-7, MDA-MB-231, U87, H1975, and BEAS-2B. Cells treated with SB4 or SB5 (10 µM) for 48 hours was evaluated.


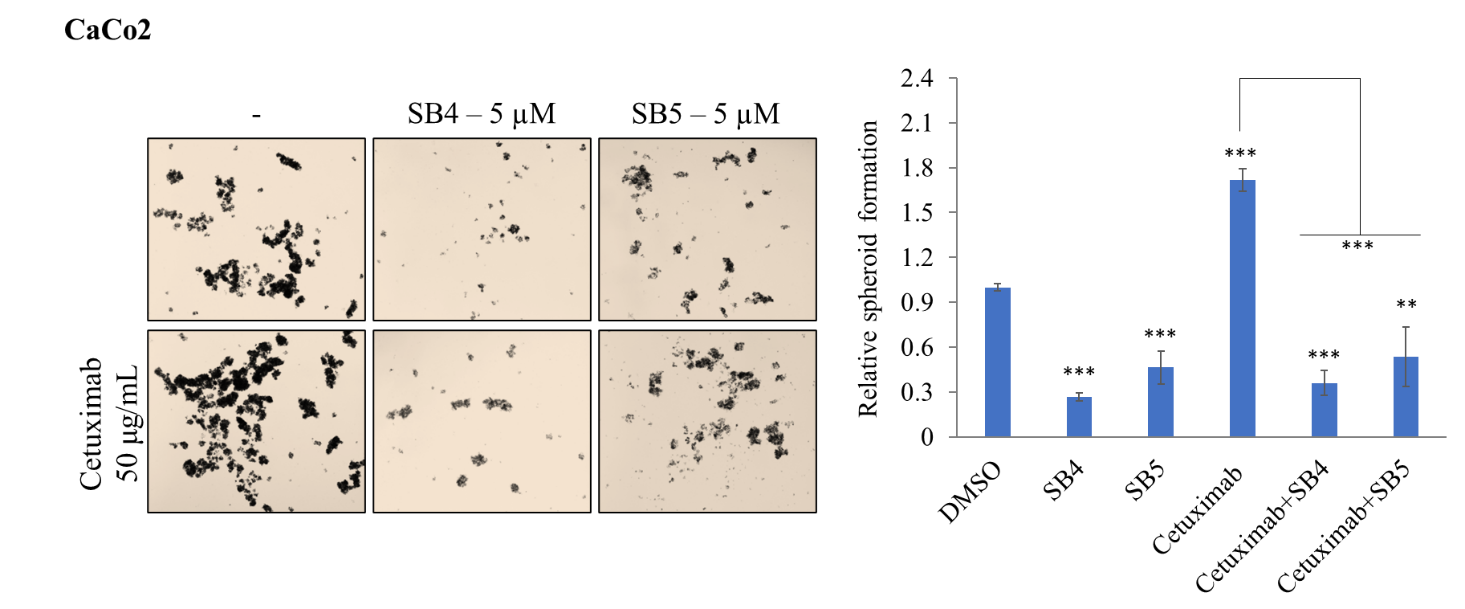
**Supplementary Figure S12. VDAC1/PHB/MMP9-binding compounds counteracts cetuximab-induced spheroid formation.** Representative images of spheroid formation by CaCo2 cells treated with cetuximab (50 μg/mL) and SB4 (5 μM) or SB5 (5 μM) for 2 weeks, and quantitative analysis of the number of spheroids formed following each treatment. Data are presented as the mean ± standard deviation, n=4. The asterisk indicates a significant difference between treatment groups, **p* < 0.05; ***p* < 0.01; ****p* < 0.001; NS, not significant.

**
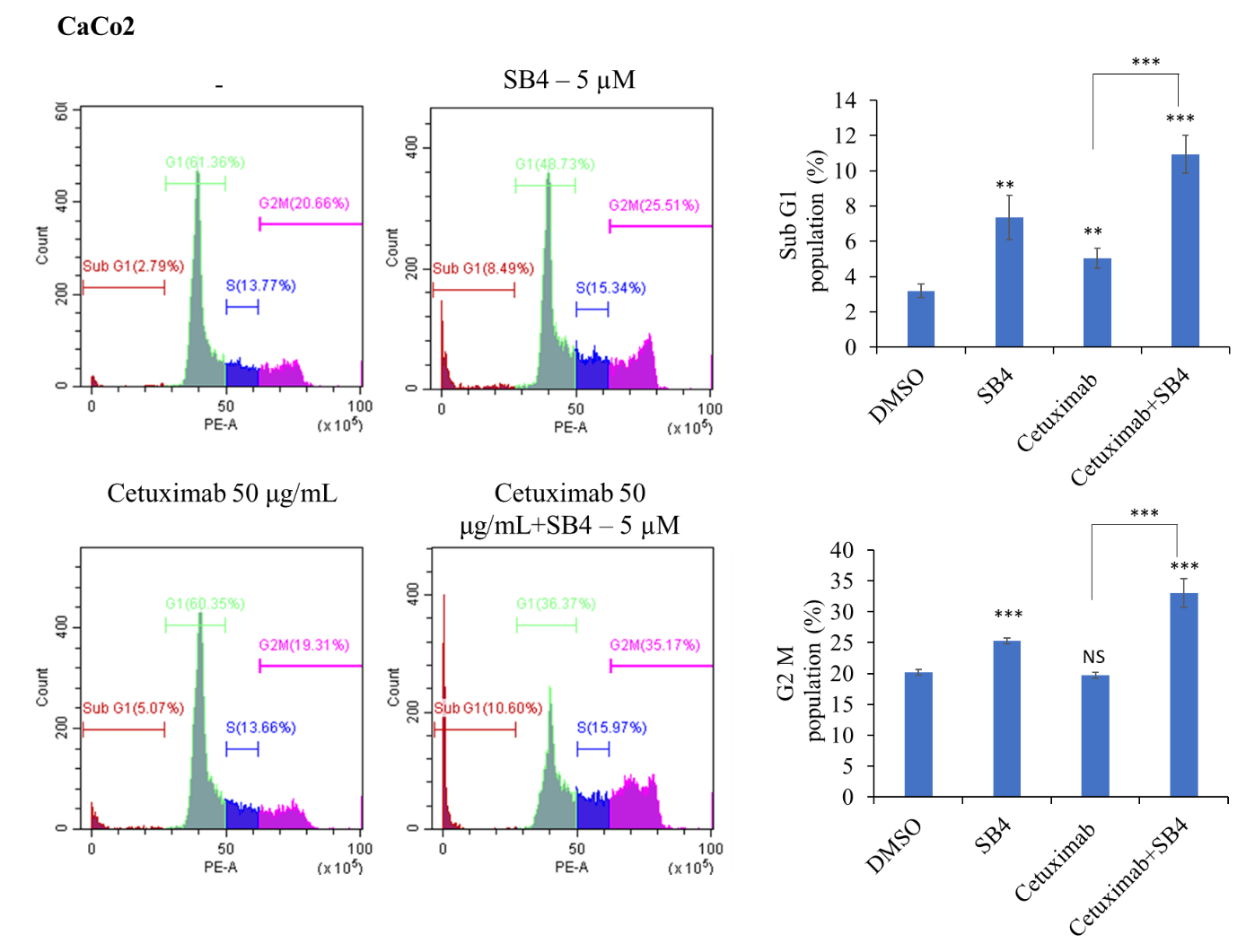
Supplementary Figure S13. SB4 synergistically modulates the cell cycle.**  Using flow cytometry, the cell cycle distribution of CaCo2 cells treated with cetuximab, SB4 at the indicated combinations for 48 hours was assessed. Quantitative measurements of sub-G1 and G2/M population are given in the graph. Data are presented as the mean ± standard deviation, n=3. The asterisk indicates a significant difference between treatment groups, **p* < 0.05; ***p* < 0.01; ****p* < 0.001; NS, not significant.


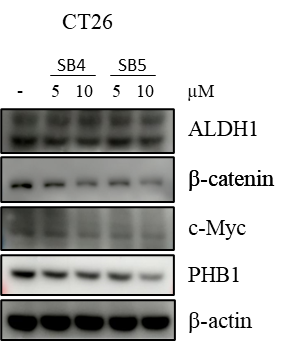


**Supplementary Figure S14.** Determination of the activity of SB4 and SB5 on murine colorectal cancer cells on several biomarker. CT26 cells were treated with SB4 and SB5 for 48 hours. After that ALDH1, β-catenin, c-Myc, PHB1 protein levels were analyzed by immunoblot assay.

**Supplementary Tables**

**Supplementary Table S1.** Mutations of the cell lines used in the study.

**
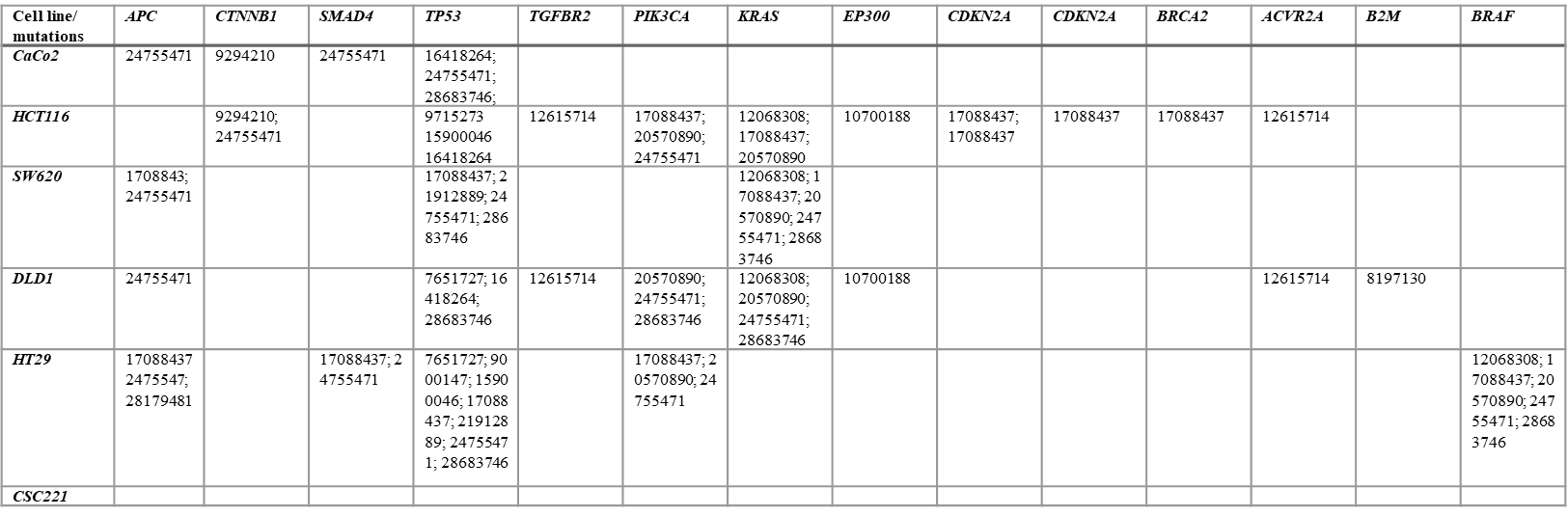
**

**Supplementary Table S2.** Antibody Information.

| **Antibody name** | **Product Information** |
| --- | --- |
| **Beta-catenin antibody** | Cell signaling, #9562 |
| **Alpha tubulin (11h101) Rabbit mAb** | Cell signaling, #2125 |
| **Goat Anti-Rabbit IgG (H+L) Peroxidase Conjugated** | Thermo scientific, 31460 |
| **Goat Anti-Mouse IgG (H+L) Peroxidase Conjugated** | Thermo scientific, NCI1430KR |
| **Phospho-STAT3 (Try705) (D3A7) XP Rabbit Antibody** | Cell signaling, #9145 |
| **Phospho-AKT (Ser473) (D9E) XP Rabbit Antibody** | Cell signaling, #4060 |
| **Phospho-mTOR (Ser2448) (D9C2) XP Rabbit Antibody** | Cell signaling, #5536 |
| **Beta-actin antibody** | Cell signaling, #4967 |
| **Hexokinase II (C64G5) Rabbit mAb** | Cell signaling, #2867 |
| **Anti-cyclin D1 Ab-3 mouse mAB (DCS-6)** | Merck, Kenilworth, NJ, USA |
| **ALDH1/2 (H8)** | sc-166362; Santa Cruz Biotechnology, Dallas, TX, USA |
| **CD133 (A3G6K) Rabbit mAb** | Cell signaling, #5860 |
| **CD44 (156-3C11) Mouse mAb #3570** | Cell signaling, #3570 |
| **Anti-GPCR GPR49 antibody (EPR3065Y), Lgr-5** | ab75850; Abcam, Cambridge, MA, USA |
| **Anti-Musashi 1 / Msi1 (EP1302** | ab52865, Abcam |
| **Gli1 (H-300)** | sc-20687; SANTA CRUZ, Dallas, TX, USA |
| **Gli2 (C-10)** | sc-271786; SANTA CRUZ |
| **Anti-Smoothened antibody** | SMO; ab72130; Abcam, Cambridge, MA, USA |
| **Anti-Bmi1 antibody** | ab38295; Abcam |
| **Bax Antibody** | Cell signaling, #2772 |
| **Bcl-xL Antibody** | Cell signaling, #2762 |
| **PARP Antibody** | Cell signaling, #9542 |
| **Caspase-3 Antibody** | Cell signaling, #9662 |
| **MMP9 Antibody** | Cell signaling, #3852 |
| **VDAC (D73D12) Rabbit mAb** | Cell signaling, #4661 |
| **PHB1 Antibody** | Cell signaling, #2426 |
| **COX IV antibody** | Cell signaling, #4844 |
| **AIF Antibody** | Cell signaling, #4642 |
| **Anti-Twist antibody** | Abcam, ab49254 |
| **PKM2 (D78A4) XP Rabbit mAB** | Cell signaling, #4053 |
| **GLUT1 (D3J3A) Rabbit mAB** | Cell signaling, #12939 |
| **Phospho-EGF Receptor (Tyr1068) Antibody** | Cell signaling, #2234 |

**Supplementary Table S3.** Primer (Forward/Reverse) sequences.

| **Gene symbol** | **Forward**  **For (5′-3′)** | **Reverse**  **Rev (5′-3′)** |
| --- | --- | --- |
| ***HK2*** | AAGGCTTCAAGGCATCTG | CCACAGGTCATCATAGTTCC |
| ***VDAC1*** | GCAAAATCCCGAGTGACCCAGA | TCCAGGCAAGATTGACAGCGGT |
| ***PHB2*** | CATCCTGTAGGTTCAGCACAAGG | CATCCTGTAGGTTCAGCACAAGG |
| ***PHB1*** | AAGCGGTGGAAGCCAAACAGGT | GCCAGTGAGTTGGCAATCAGCT |
| ***MMP9*** | TTGACAGCGACAAGAAGTGG | CAGTGAAGCGGTACATAGGG |
| ***β-actin*** | ATTGTGAACTTTGGGGGATG | GATGAGATTGGCATGGCTTT |
| ***ALDH1*** | TGTTAGCTCATGCCGACTTG | TTCTTAGCCCGCTCAACACT |
| ***CD44*** | TGCCGCTTTGCAGGTGTAT | GGCCTCCGTCCGAGAGA |
| ***CD133*** | GGACCCATTGGCATTCTC | CAGGACACAGCATAGAATAATC |
| ***Lgr-5*** | CTCTTCCTCAAACCGTCTGC | GATCGGAGGCTAAGCAACTG |
| ***Msi-1*** | ACCAAGAGATCCAGGGGTTT | TCGTTCGAGTCACCATCTTG |
| ***Hes-1*** | CTGAAGAAAGAT AGCTCGCG | ACTTCCCCAGCACACTT |
| ***EphB-1*** | TGCAAGGAGACCTTCAACCT | CGGTGTTGATTTTCATGACG |
| ***GAPDH*** | ATCACCATCTTCCAGGAGCGA | AGTTGTCATGGATGACCTTGGC |
| ***β-catenin*** | AAAATGGCAGTGCGTTTAG | TTTGAAGGCAGTCTGTCGTA |
| ***c-Myc*** | AATGAAAAGGCCCCCAAGGTAGTTATCC | GTCGTTTCCGCAACAAGTCCTCTTC |
| ***Cyclin-D1*** | CCGTCCATGCGGAAGATC | GAAGACCTCCTCCTCGCACT |
| ***CDH2*** | CTCCTATGAGTGGAACAGGAACG | TTGGATCAATGTCATAATCAAGTGCTGTA |
| ***SNAIL*** | TCCCGGGCAATTTAACAATG | TGGGAGACACATCGGTCAGA |
| ***ZEB2*** | CAAGAGGCGCAAACAAGCC | GGTTGGCAATACCGTCATCC |
| ***SLUG*** | CGAACTGGACACACATACAGTG | CTGAGGATC TCTGGTTGTGGT |
